# A 41,500 year-old decorated ivory pendant from Stajnia Cave (Poland)

**DOI:** 10.1038/s41598-021-01221-6

**Published:** 2021-11-25

**Authors:** Sahra Talamo, Wioletta Nowaczewska, Andrea Picin, Antonino Vazzana, Marcin Binkowski, Marjolein D. Bosch, Silvia Cercatillo, Marcin Diakowski, Helen Fewlass, Adrian Marciszak, Dragana Paleček, Michael P. Richards, Christina M. Ryder, Virginie Sinet-Mathiot, Geoff M. Smith, Paweł Socha, Matt Sponheimer, Krzysztof Stefaniak, Frido Welker, Hanna Winter, Andrzej Wiśniewski, Marcin Żarski, Stefano Benazzi, Adam Nadachowski, Jean-Jacques Hublin

**Affiliations:** 1 Department of Human Evolution, Max Planck Institute for Evolutionary Anthropology, Deutscher Platz 6, 04103 Leipzig, Germany; 2 Department of Chemistry G. Ciamician, University of Bologna, Via Selmi 2, 40126 Bologna, Italy; 3 Department of Human Biology, University of Wrocław, ul. Przybyszewskiego 63, 51-148 Wrocław, Poland; 4 Department of Cultural Heritage, University of Bologna, Via degli Ariani 1, 48121 Ravenna, Italy; 5 X-Ray Microtomography Lab, Department of Biomedical Computer Systems, Institute of Computer Science, Faculty of Computer and Materials Science, University of Silesia, Będzińska 39, 41-200 Sosnowiec, Poland; 6 Vienna Institute for Archaeological Science, University of Vienna, Franz-Klein-Gasse 1, 1190 Vienna, Austria; 7 Turkana Basin Institute Ltd, Turkana, Kenya; 8 Turkana Basin Institute, Stony Brook University, N-507 Social and Behavioural Sciences, NY 11794-4364 Stony Brook, USA; 9 Department of Stone Age Archaeology, Institute of Archeology, University of Wrocław, Szewska 48, 50-139, Wrocław, Poland; 10 Department of Paleozoology, University of Wrocław, Sienkiewicza 21, 50-335 Wrocław, Poland; 11 Department of Archaeology, Simon Fraser University, Burnaby, BC V5A, 1S6 Canada; 12 Department of Anthropology, University of Colorado Boulder, Boulder, CO 80309 USA; 13 Centre for the Exploration of the Deep Human Journey, University of the Witwatersrand, Johannesburg, Gauteng South Africa; 14 Evolutionary Genomics Section, Globe Institute, University of Copenhagen, Copenhagen, Denmark; 15 Polish Geological Institute-National Research Institute, Rakowiecka 4, 00-975 Warsaw, Poland; 16 Institute of Systematics and Evolution of Animals, Polish Academy of Sciences, Sławkowska 17, 016 Kraków, Poland; 17 Collège de France, 11 Place Marcellin Berthelot, 75005 Paris, France

**Keywords:** Anthropology, Archaeology, Cultural evolution, Evolution

## Abstract

Evidence of mobiliary art and body augmentation are associated with the cultural innovations introduced by *Homo sapiens* at the beginning of the Upper Paleolithic. Here, we report the discovery of the oldest known human-modified punctate ornament, a decorated ivory pendant from the Paleolithic layers at Stajnia Cave in Poland. We describe the features of this unique piece, as well as the stratigraphic context and the details of its chronometric dating. The Stajnia Cave plate is a personal 'jewellery' object that was created 41,500 calendar years ago (directly radiocarbon dated). It is the oldest known of its kind in Eurasia and it establishes a new starting date for a tradition directly connected to the spread of modern *Homo sapiens* in Europe.

## Introduction

The emergence of decoration and adornment of the human body is considered one of the earliest manifestations of symbolic behavior, marking the beginning of ethnolinguistic identity and social complexity in human evolution^1,2^. Timing when and where personal ornaments appeared in the archaeological record are important for reconstructing the trajectories of abstract thinking of archaic humans and understanding how figurative representations varied through time^1,2^. In Europe, the oldest evidence of body adornment is documented at ~ 46 ka BP in the Initial Upper Paleolithic layers of Bacho Kiro where several carnivore teeth were worked into pendants^3,4^. A successive technical advancement is recorded in the Early Aurignacian (~ 40 ka BP) when mammoth ivory started to be manipulated for the production of pendants and mobiliary arts^5–7^. Within these novel accessories, a new type of decoration—the alignment of punctuations—emerged on some ornaments in south-western France^8^, and figurines in Swabian Jura (Germany)^9^. Thus far, most of these iconic adornments were recovered during older excavations, with less recognition of site formation histories and post-depositional disturbance. Hence, their chronological attribution is based only on the stratigraphic context rather than direct dating. Recent chronometric programs on sites in Swabian Jura^10^ yielded contradictory results corroborating the inaccurate provenience of the samples collected during previous fieldwork. This situation makes the reconstruction of the emergence of human body augmentation and the discussion concerning the epicenter of the diffusion of mobiliary art in Europe (*Kulturpumpe* model)^10^ hotly debated and far from being resolved^10–12^.

In this context, we report here the discovery and the direct date of a new ivory punctate ornament found at Stajnia Cave, in Poland. This finding plays a unique role in demonstrating the importance of the direct date of an object of Paleolithic art to understand the origin of communication, celebration, and expression of *Homo sapiens* in Europe. 

**Figure 1:**
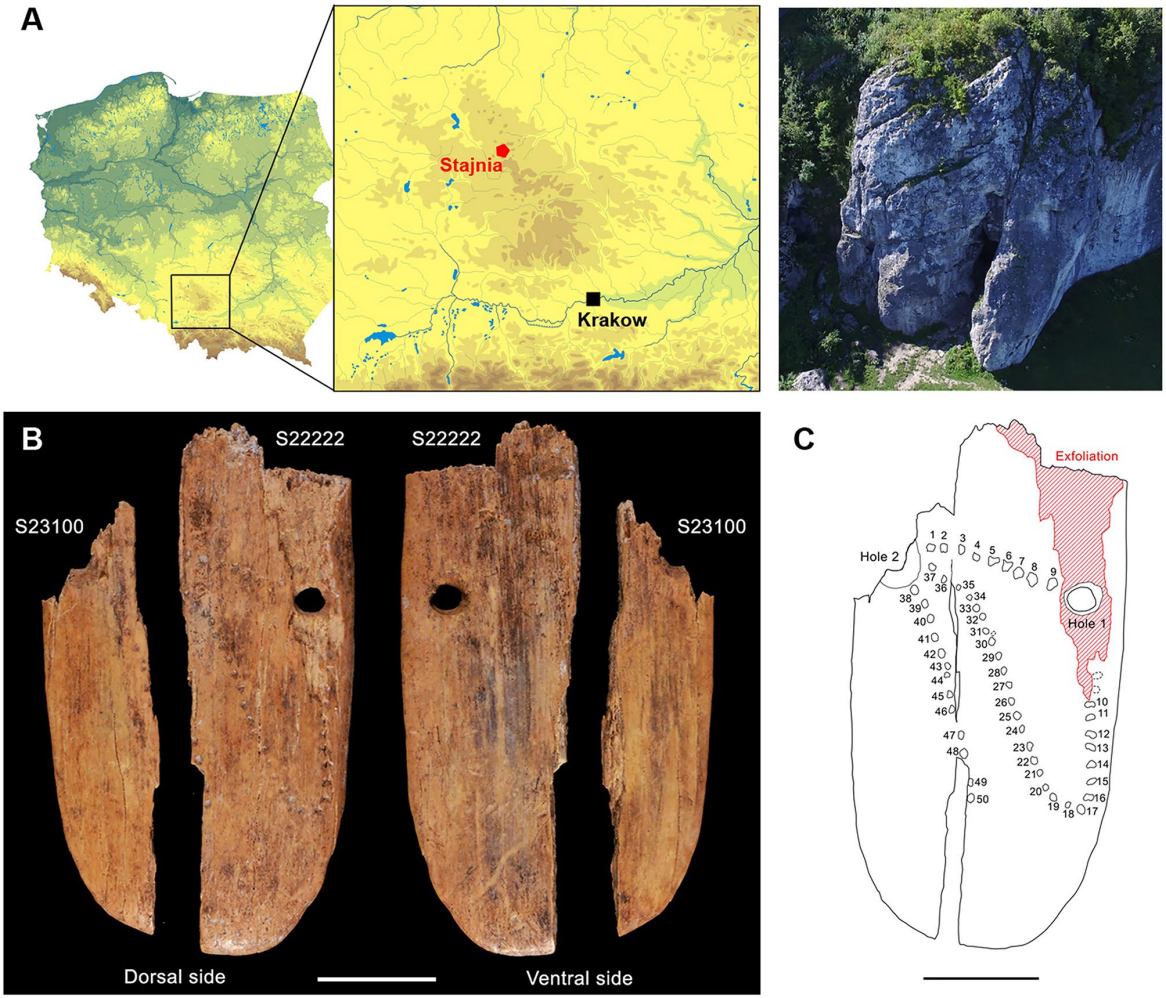
Stajnia pendant and location of the site. (**A**) Site location in southern Poland ( modified from https://pl.wikipedia.org/wiki/ Geografia_Polski#/media/Plik:Physical_map_of_Poland.png, CC BY-SA 4.0) and aerial picture of Stajnia Cave; (**B**) Dorsal and ventral views of the pendant (S23100, S22222). Scale bar is 1 cm. (**C**) Schematic representation of the pendant (dorsal view). Numbers 1 to 50 indicate clearly identifiable punctuations; dotted lines indicate possible punctuations. The red hatch indicates the exfoliated area. Scale bar is 1 cm.

**Figure 2:**
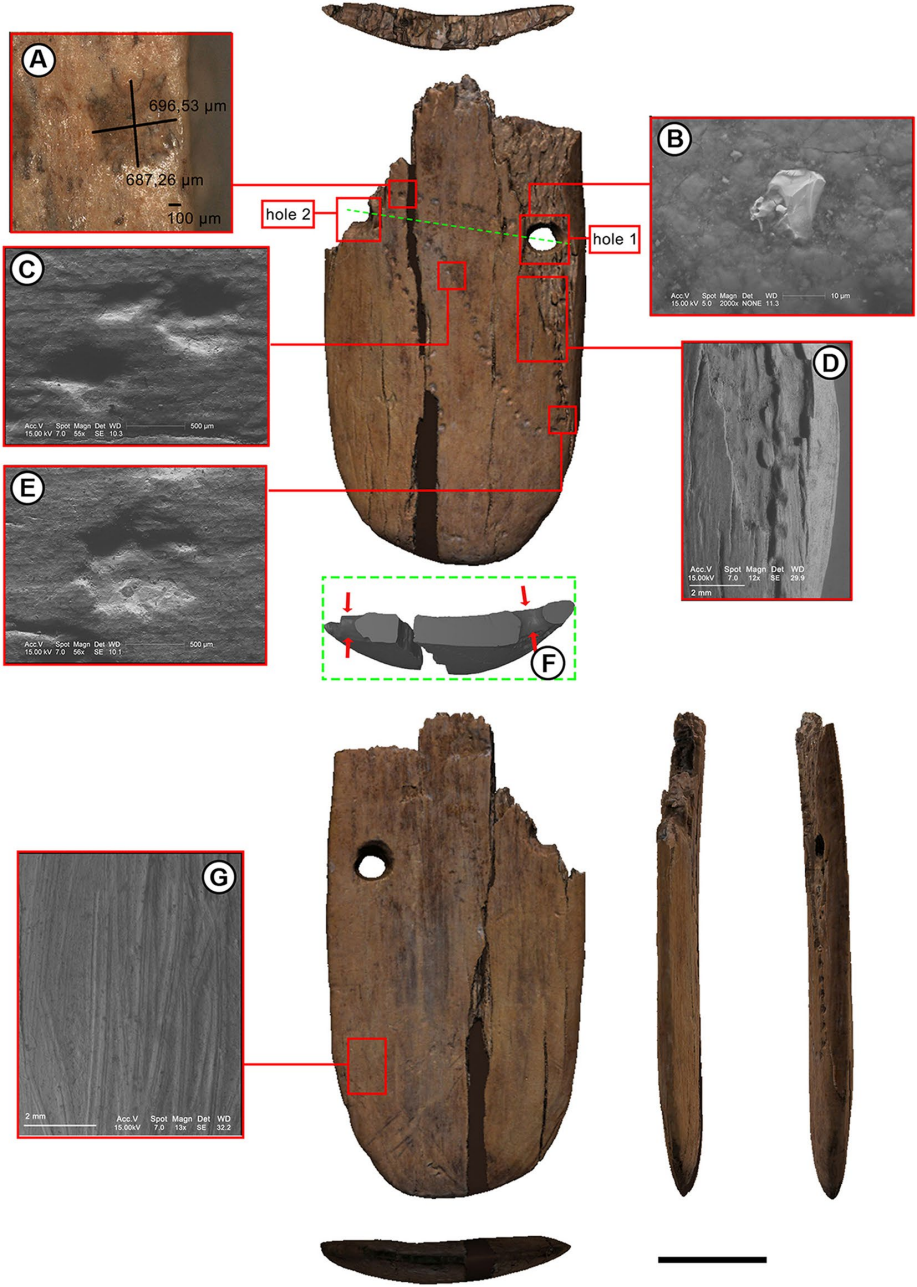
Stajnia pendant reconstruction. Views of the virtually reconstructed pendant and photomicrographs documenting the technology used for their manufacture: multiple examples of punctures (**A**,**C**–**E**) and traces of smoothing (**B**,**G**). A longitudinal section through perforations is shown in (B). Scale bar is 1 cm.

**Figure 3:**
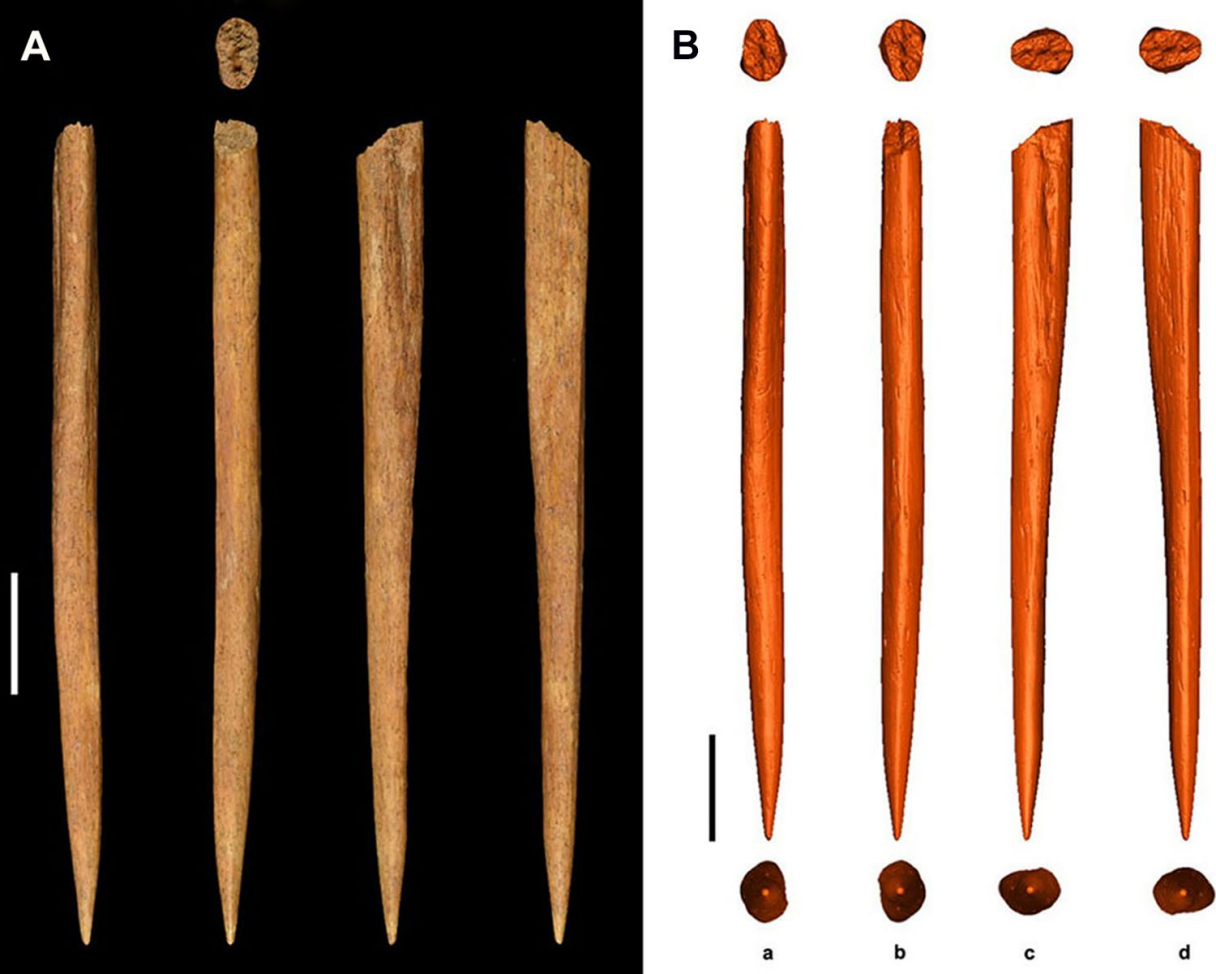
Stajnia awl. (**A**) Original picture of the awl from Stajnia Cave; (**B**) Reconstructed 3D digital models of the awl. Scale bars are 1 cm.

The Stajnia Cave is a natural shelter located on the northern side of the Kraków-Częstochowa Upland in southern Poland (50° 36′58″ N, 19°29′04″ E) (Fig. 1a). The site was investigated between 2006 and 2010 exposing a stratigraphic sequence of seven units (from G at the bottom (MIS 5c), to A (MIS 1) at the top) (Supplementary Sect. 1 and Fig. S1). During the excavations, a series of Neanderthal remains were found^13,14^ within a large collection of bones of Late Pleistocene steppe-tundra species, and Middle and Upper Paleolithic artefacts^14^ (see Supplementary Sects. 1 to 4). In 2010, two fragments of an ornate ivory pendant (S-22222 + S-23100) were discovered in layer D1 (Figs. 1b,c, and 2). In addition, an awl fragment (S-12160) was identified among the bone fragments from layer D1 (Fig. 3). A recent reassessment of the archaeological record of Stajnia Cave reveals that post-depositional frost disturbances and modern distortions displaced artefacts and human remains between layers^14^. Since most of the lithics collected in layer D1 are associated with the Central and Eastern European Micoquian and very few are classified as Upper Paleolithic (Supplementary Figs. S3 and S4), the accurate cultural attribution of the pendant and the awl required direct radiocarbon dating. In order to minimise the amount of material exposed to destructive analysis, the most recent methodological advancements in ^14^C were followed^15,16^.

## Results

### The pendant and the awl

The pendant is characterised by an oval shape with rounded margins, two drilled holes and decoration consisting of patterns of sequential punctures. The largest piece of the pendant is 4.5 cm long and 1.5 cm wide while the thickness varies between 0.36 and 0.39 cm. The reconstructed width of the complete artefact is shown in Fig. 2. There is one fully preserved perforation visible on the largest piece (hole 1 in Fig. 2) located close to the centre of the reconstructed artefact, near its upper edge. Another hole (hole 2 in Fig. 2), initially located near the opposite edge of the artefact, is partly preserved. The diameter of the fully preserved hole 1 is 2.3 mm and the original diameter of the partly preserved hole 2 was probably the same. The dorsal surface of the object is ornamented with at least 50 punctures creating an irregular looping curve (Fig. 1c). The ornamentation is partly destroyed by exfoliation which occurred close to the hole 1 (Figs. 1c, 2d. Besides this exfoliation, longitudinal cracks are also visible on the surface of the object.

Scanning electron microscopy (SEM) was conducted to verify the artefactual character of the observed features and to identify the technology used for their manufacture. The SEM analysis (Fig. 2b–e,g) indicates that the dorsal surface of the pendant does not present clear traces of intentional preparation preceding the creation of the punctures. The ventral puncture, however, presents traces of smoothing (Fig. 2g) which are linear and parallel to the longest axis of the artefact. The V-shaped cross-sections of the marks suggest the use of a flint artefact (Fig. 2b,g), and the differences in depth and width of the striations may be explained by the irregular edge of the applied stone tool^17^. Hole 1 and hole 2 were artificially manufactured by drilling from both sides which were not thinned previously, resulting in a biconical shape in cross-section (Fig. 2f). Most of the punctures are similar in terms of their outlines and cross-sections (Fig. 2c,e), which makes it highly probable that they all were made with the same tool—possibly in a relatively short time^18^. Punctures located directly below the fully preserved hole 1 display a slightly different morphology with less defined edges (Fig. 2a). The possibility that these punctures were made at a different time than the others cannot be excluded, however, gradual tool wear or a changed position of the tool are more parsimonious.

The maximum length of the awl is 68.33 mm (Fig. 3). Several wear facets are visible along the awl surface, and the basal cross-sections (5.8 × 3.4 mm) is flattened (Fig. 3). On the bottom side, there is a smoothed surface with round pronounced edges and flattening spike. The top side is more concave, and towards the tip, an extremely smooth facet is responsible for further refining. The lateral sides of the spike are rounded and polished. At c. 38.18 mm from the spike, the awl becomes basally thicker. Clear evidence of bone working is shown at the bottom facet, which has sharp edges towards both sides and the round spike show evidence of wear signs, indicating that an extensive use before discarding (Fig. 3).

Zooarchaeology by mass spectrometry (ZooMS) analysis reveals the pendant to be made from mammoth ivory and the awl from a horse bone (Supplementary Sect. 5).

### The dating

Bones and ivory are the most suitable and well-established osseous materials to attempt radiocarbon dating^15,19,20^. The presence of collagen in the pendant (R-EVA 2651) and awl (R-EVA 2650) were tested using the near-infrared (NIR) analysis before sampling for radiocarbon dating. The results indicate that both specimens are well preserved and predicted yields 5.30 ± 1.52% (Pendant) and 8.04 ± 1.43% (Awl) weight collagen (Supplementary Fig. S6), which align closely with the collagen yields obtained following extraction (Table 1).

**Table 1:** The results of AMS radiocarbon dating and OSL from Stajnia.

MPI lab code	Level	Square	Submitter no	Species	Start mass mg	mg of collagen	Collagen %	C:N	Lab code	^14^C Age BP	Err 1σ	Cal BP 68.3% From-To		Cal BP 95.4% From-To		References
	B			*Saiga tatarica*					Poz-28891	13,500	50	16,360	16,180	16,470	16,080	^38^
	B			–			–	–	OSL-GdTL-1126	8,950	–					^51^
R-EVA 2475	C18	9F	S-5570	Mammoth tusk fragment	567,9	17,3	3,0	3,1	ETH-110248.1.1	> 50,000						This paper
R-EVA 779*	C18	10D	S-12507	UNG-reindeer or Ibex	600	15,1	2,5	3,1	MAMS-19870	40,400	420					This paper
	C18			Bear				3,6	Poz-61719	20,930	140	25,550	25,050	25,680	24,910	^53^
	C18			Bear				3.5	GdA-3894	21,900	90	26,280	26,000	26,370	25,930	^52^
R-EVA 729*	C19	5F	S-11613	UNG-reindeer or Ibex	794	42,8	5,4	3,2	MAMS-19849	33,450	350	38,930	37,680	39,290	37,180	This paper
R-EVA 739*	C19	5E	S-13694	*Rangifer tarandus*	600	28,2	4,7	3,3	MAMS-19851	36,080	460	41,550	40,740	41,970	40,370	This paper
R-EVA 768	C19	7D	S-11340	UNG-reindeer or Ibex-Bos/Bison or Horse	949	114,5	12,1	3,2	MAMS-19864	37,750	310	42,320	42,060	42,440	41,900	This paper
	D1			–			–	–	OSL-GdTL-1127	45,900	–					^51^
	D1	7F	S-23101b	Mammoth			6,9	3,1	OxA-24944	44,600	2100	49,850	45,060	…	44,420	^51^
R-EVA 793 = S-EVA 27827	D1	11D	S-12182	UNG-reindeer or Ibex	530,2	70,2	13,2	3,2	MAMS-19879	44,590	690	47,610	46,130	48,470	45,630	^14^
R-EVA 750	D1	7F	S-24106	UNG Bos/Bison or Horse	569,7	21,3	3,7	3,1	MAMS-19857	45,020	1380	49,000	45,890	…	45,020	This paper
R-EVA 2651 Pendant	D1	7F	S-22222	Woolly mammoth	359,2	17,6	4,9	3,3	MAMS-35153	36,600	300	41,730	41,340	41,900	41,210	This paper
									ETH-99043.1.1	36,563	229					
R-EVA 2650 Awl	D1	5E	S-12160	Equidae	343,9	27,5	8,0	3,2	MAMS-35152 1	37,360	330	42,270	42,070	42,360	41,960	This paper
									ETH-99042.1	37,903	267					
R-EVA 742	D1	6D	S-11609	Bos/Bison	663,2	66,8	10,1	3,2	MAMS-19853	45,300	1410	49,420	46,060	…	45,100	This paper
R-EVA 735	D1	6E	S-24737	UNG Bos/Bison or Horse	849,3	68,3	8,0	3,3	MAMS-19850	> 49,000						This paper
R-EVA 2470	D1	5E	S-16187	Mammoth tusk fragment	814,4	44	5,4	3,1	ETH-110246.1.1	> 50,000						This paper
	D1			Cave bear tooth					Poz-28892	> 49,000						^52^
R-EVA 740*	D1	5D	S-24390	UNG Bos/Bison or Horse	654,6	46,8	7,1	3,3	MAMS-19852	> 49,000						This paper
R-EVA 766*	D1	7D	S-9547	UNG-reindeer or Ibex	852,3	47	5,5	3,2	MAMS-19863	> 49,000						This paper
R-EVA-2469	D2	5E	S-17162	Mammoth tusk fragment	606,3	25,2	4,2	3,1	ETH-110247.1.1	> 50,000						This paper
R-EVA 789 = S-EVA 27823	D2	11D	S-12305	UNG-woolly rhinoceros, woolly mammoth	632	74,2	11,7	3,2	MAMS-19878	> 49,000						^14^
	D2			-					U/Th W1400 + W1417	52,900	1900					^52^
R-EVA 751	D2	7F	S-23855	UNG-reindeer or Ibex	552,3	13,1	2,4	3,1	MAMS-19858	> 49,000						This paper
R-EVA 780 = S-EVA 27814	D3	11F	S-12722	Bos/Bison	678,6	75,3	11,1	3,1	MAMS-19871	> 49,000						^14^
R-EVA 778 = S-EVA 27812	D3	11F	S-11572	UNG-reindeer or Ibex	595,6	99,1	16,6	3,2	MAMS-19869	> 49,000						^14^
R-EVA 743 = S-EVA 27777	E	6F	S-24262	UNG Bos/Bison or Horse	842,1	85,3	10,1	3,1	MAMS-19856	> 49,000						^14^

For 20 samples, stable isotopic analysis was evaluated at MPI-EVA, Leipzig (Lab Code S-EVA), using a ThermoFinnigan Flash EA coupled to a Delta V isotope ratio mass spectrometer. The bones with human modifications are indicated by an asterisk in the MPI Lab Code. Results are rounded to the nearest 10 years.

Collagen was extracted from both specimens at the Max Planck Institute for Evolutionary Anthropology (MPI-EVA) in Leipzig, Germany. The collagen from the pendant and the awl was radiocarbon dated twice with an Accelerator Mass Spectrometer (AMS) at two different radiocarbon laboratories (MAMS and ETH) in order to obtain very precise ^14^C dates for calibration with the recently updated IntCal20 calibration curve^21,22^ (Table 1). The combined ^14^C age for the pendant (S-22222) is 36,577 ± 183 ^14^C BP (obtained using the R_Combine command in OxCal 4.4.2^23^), and the combined ^14^C age of the awl (S-12160) is 37,701 ± 208 ^14^C BP, which correspond respectively to calibrated ranges of 41,730–41,340 cal BP and 42,270–42,070 cal BP at 68.3% probability (Table 1, and Supplementary Table S5). From the 20 animal samples pretreated at the MPI-EVA, 11 are older than 49,000 years BP, one from layer E, two from layer D3, three from layer D2, four from layer D1, and one from layer C18. In layer D1, five more samples result in finite ages from 45,300 ± 1410 to 36,577 ± 183 BP, including the pendant and the awl samples. Three dates from layer C19 ranges from 37,750 ± 310 to 33,450 ± 350 BP and one from the top of layer C18 gives a very old age compared with the C19 layer below (MAMS-19870: 40,400 ± 420 BP) (Table 1). Mammoth ivory tusk fragments from layers D2, D1 and C19 are older than 50,000 years, whereas another ivory fragment from D1 was previously dated 44,600 ± 2,100 BP (OxA-24944) (Table 1).

We then constructed a Bayesian chronological model using the software OxCal 4.4^23^ and the new IntCal20 curve^21^ to refine the calibrations of the radiocarbon dates of Stajnia Cave. The calibrated dates (un-modelled in Table 1) and the modelled ages obtained are shown in Supplementary Table S5 and Fig. S7. We did not include dates > 49,000 BP in the model. As is evident from Supplementary Fig. S7, the lowermost layers of the cave (layers E, D3 and D2) extend beyond the range of the radiocarbon method. Five further dates in layer D1 and one date in layer C18 are also > 49,000 BP, even though these layers contain Upper Paleolithic artefacts. This demonstrates the poor agreement between the high-resolution ^14^C dates and the poor resolution of the stratigraphy at the site, resulting in a model agreement index of 34.5% with four outliers (higher than 20%) out of 14 modelled samples. This situation implies that the awl and the pendant (32% and 21% outlier probability respectively), found in layer D1, have likely moved between layers and probably originate from layer C19 rather than layer D1. This hypothesis is corroborated by the radiocarbon age of two bones from layer C19 that have similar chronological ranges to the awl and pendant (Table 1). The sample R-EVA 739 (MAMS-19851: 36,080 ± 460 BP) also shows anthropogenic modifications suggesting a close association between the human settlement of the cave and the ivory pendant.

## Discussion

**Figure 4:**
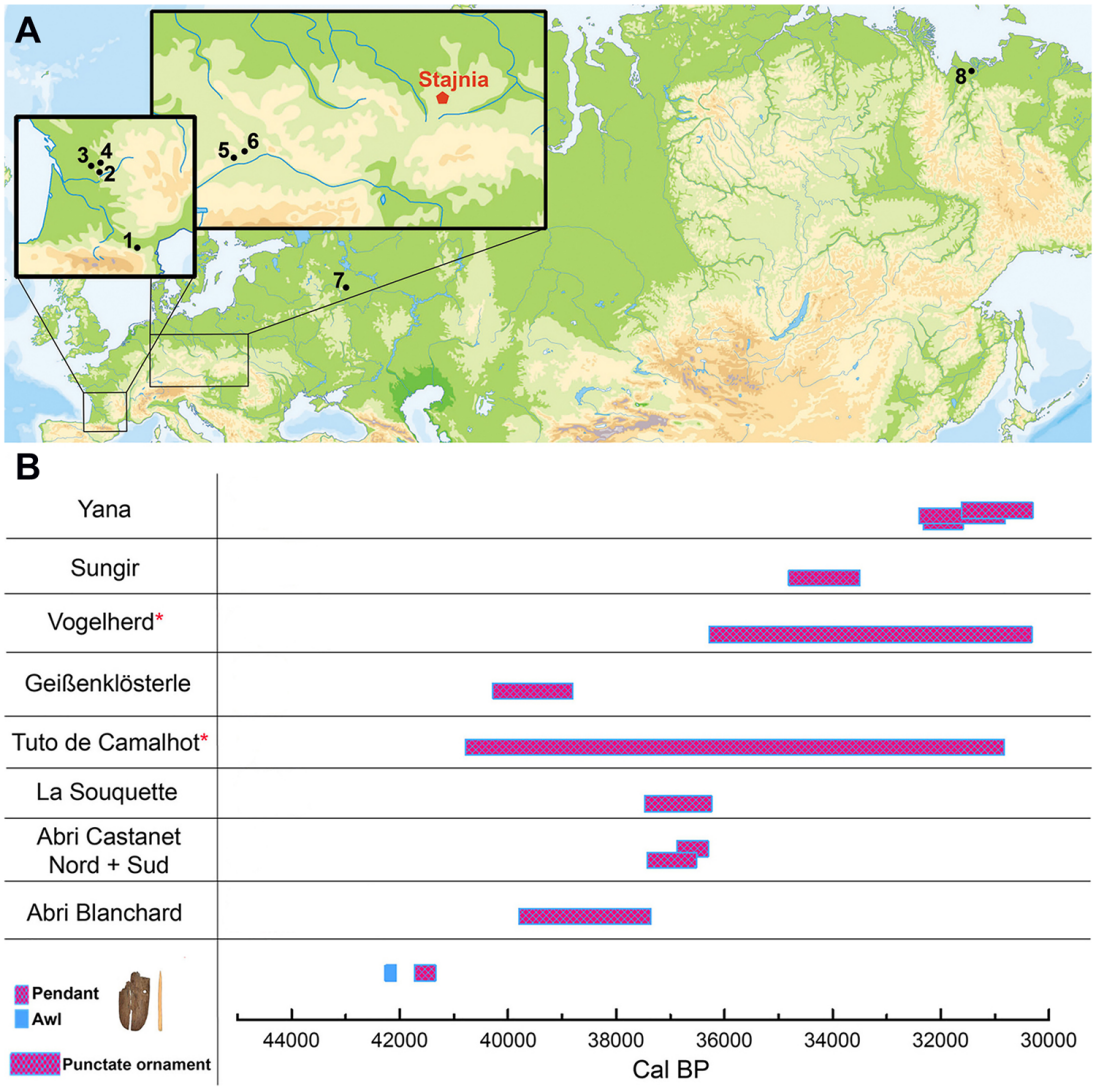
Map of the geographical distribution of the sites where punctuated ornaments and objects have been found. (**A**) Map of the geographical distribution of the sites where punctuated ornaments and objects with punctate decoration have been found in Aurignacian and Early Upper Palaeolithic contexts (1 Tuto de Camalhot, 2 Abri Blanchard / Abri Castanet, 3 Abri la Souquette, 4 Abri Lartet, 5 Geißenklösterle, 6 Vogelherd, 7 Sungir, 8 Yana); (**B**) Chronological comparison of Stajnia pendant and awl (calibrated ranges) with other artistic punctate ornaments found in Upper Palaeolithic sites (modelled ranges). The horizontal bars show the calibrated ranges of direct dates of the awl in blue and of the pendant in pink cross-hatched. From the other sites, the age range of the layers where punctate ornaments have been found are pink cross-hatched bars and are the modelled date ranges produced using the 'date' command in OxCal (See Table S14). The red asterisk close to the name of the sites indicates a 'hypothetical' boundary imposed by the Bayesian model due to a very low agreement index for Vogelherd and just two samples out of context for Tuto de Camalhot. All the bars represent 68.3% probability.

The direct radiocarbon date makes the Stajnia ornate pendant (41,730–41,340 cal BP (68.3%)) the earliest punctate ivory object known to date to the Early Upper Palaeolithic record in Eurasia (Fig. 4b, Table 1). Although the Aurignacian settlement at Stajnia Cave was ephemeral (Supplementary Sect. 4), the direct radiocarbon dates on the pendant and the awl establish that the dispersal of these elaborate and highly manufactured objects, as forms of cultural innovation with highly symbolic values by *Homo sapiens*, was established by around 42,000 cal BP. The radiocarbon dating on other ivory fragments reveals the transport on-site of mammoth tusks since the Middle Paleolithic (Table 1), but only during the Early Aurignacian, this raw material was worked for the production of mobiliary art.

We consider the possibility that the age of the pendant itself is much older than the decoration carved upon it to be unlikely given the experimental and chronological data presented here. The direct ages of the two precious objects correspond to the chronological range of layer C19 suggesting a short-term occupation at the site during the Aurignacian rather than a chronological coincidence.

Although permafrost may allow perfect preservation of mammoth tusks in open-air sites for millennia, these conditions are absent during MIS 3 and MIS 2 in southern Poland^24^. This evidence implies that over thousands of years the mammoth tusk was likely subjected to taphonomic processes causing progressive deterioration of the ivory. As shown in our replicative experiment (see Supplementary Sect. 8), using a subfossil and desiccated tusk fragment in middle/poor condition would have been unworkable for shaping and decorating an ornament aslike the one found in Stajnia. Therefore, we assume that the shaping and punctate decoration was made on a mammoth tusk in fresh condition corroborating the age of ~ 41,500 cal BP.

Determining precisely when the punctate ornaments emerged in Eurasia required comparison with the other archaeological sites where this artistic pattern was found (Fig. 4). At Geißenklösterle Cave (Germany), punctuations were identified in horizon IIb (an ivory anthropomorph shows a regular punctate decoration on the backside) ranging between 40,280–38,800 cal BP (68.3%) (new modelled calibrated ranges with IntCal20 in Supplementary Sect. 7, and in Supplementary Tables S6, S7 and S14). In France, the use of the punctate motif emerged during the Early Aurignacian at Tuto de Camalhot (40,790–30,830 cal BP (68.3%), new modelled calibrated ranges with IntCal20 in Supplementary Sect. 7, and in Supplementary Tables S11 and S14) and only during a later phase in several sites located in the Castel-Merle Valley^18^ ranging between 39,800 and 36,240 cal BP (68.3%) (new modelled calibrated ranges with IntCal20 in Supplementary Sect. 7, and Supplementary Tables S8-S10 and S14). However, our model output reveals a low agreement index and poor stratigraphic integrity for Vogelherd Cave. At Tuto de Camalhot Cave, the boundaries obtained from the Bayesian model should be considered 'hypothetical' because they are based on two bones without any stratigraphic information. Further east, patterns of sequential punctures on ivory pendants were made during the EUP at the open-air sites of Sungir^25^ in Russia (34,810–33,500 cal BP (68.3%), new modelled calibrated ranges with IntCal20 in Supplementary Sect. 7, and Supplementary Tables S12 and S14), and at Yana^26^ in the Siberian Arctic (32,400–30,820 cal BP (68.3%), new modelled calibrated ranges with IntCal20 in Supplementary Sect. 7, and Supplementary Tables S13 and S14). This evidence reveals a broad geographical distribution of punctate graphic representation (Fig. 4a), and it shows that in Eurasia, the punctate decoration of the pendant at Stajnia Cave predates other instances of this type of ornamentation activity by 2000 years (Fig. 4b and Supplementary Table S14).

A deeper examination of the beginning of the diffusion of mobiliary art and body augmentation in Eurasia shows some chronological uncertainties (Supplementary Sect. 7). While at Sungir, the direct dates on the buried individuals^25^ give a precise indication of the age of the ivory beads, at Yana post-depositional processes (e.g., colluviation, solifluction, or ice drift)^26^ could have displaced some pendants from their original position. In Europe, apart from Geißenklösterle, all the personal ornaments were discovered during excavations carried out in the late 19^th^ and the early twentieth century and are associated only indirectly with the Early or Recent Aurignacian (SI Sect. 7). At Geißenklösterle, the chronology is well established for the different Aurignacian levels^27^ (new ranges with IntCal20 in Supplementary Sect. 7 and Supplementary Table S6). In contrast, the low chronological resolution of the other Early Upper Paleolithic sites impedes a clear understanding of the diachronic development of Aurignacian artistic expression. This situation is mainly due to the poorly constrained ^14^C dating resolution caused by questionable stratigraphic contexts at the sites^10^ (Supplementary Sect. 7). In the light of the Stajnia pendant, the model that the Swabian Jura was the centre of the diffusion of artistic innovations (*Kulturpumpe* hypothesis)^10^ needs further examination.

### Summary and conclusion

The punctate decorative motif is one of the artistic innovations that developed during the Early Aurignacian^1,28^ in Europe and the EUP in the Russian Plains^26,29^. Thus far, these marks on mobile objects have been interpreted as hunting tallies, arithmetic counting systems, or lunar notation^18^, whereas others have suggested aesthetic purposes^7^. The looping curve represented on the Stajnia pendant is similar to the engraved patterns of the Blanchard plaque^18^. Whether these marks indicate cyclic notations or kill scores remain an open question, although the resemblance with the lunar analemma is striking. In other personal ornament and ivory objects, the use of the punctate pattern is easier to identify as the makers tried to imitate and transfer natural patterns in new contexts^7^. These are the reproductions of the coat of a feline and a trout at Vogelherd^5,30^, the replication of different types of shells at La Souquette, Abri Castanet, and Tuto de Camalhot^8^, or the imitation of the coat of a horse at Sungir^29^. In addition, the punctures could serve as simple decoration as seen on the backside of the anthropomorph at Geißenklösterle^10^, the perforated baton at Sungir^29^, and on ivory diadems and needles at Yana^26^. A precise cross-cultural comparison of the emergence of mobiliary art and body augmentation, especially in Europe, requires direct radiocarbon dating of some of these figurines and ornaments to solve the debated questions concerning contemporaneity and socio-cultural connections between groups of *Homo sapiens* at the onset of the Upper Palaeolithic.

Investigating Palaeolithic art using the precise ticking of the radiocarbon clock is challenging, especially when it involves the destruction of precious and unique artefacts. However, combining updated radiocarbon pretreatment^15^, NIR spectroscopy pre-screening to non-destructively quantify collagen preservation^16^ and the latest AMS instrumental advances (e.g., increasingly precise error ranges^4^), with the new IntCal20^21^ calibration curve, we can overcome previous limitations to the direct dating of small, highly precious ornaments and instead associate them directly with a radiocarbon date of centurial precision.

The age of ~ 41,500 cal BP of the decorated ivory pendant from Stajnia Cave underlines the importance of directly dating mobiliary art to solve the intriguing puzzle of the emergence of symbolic behaviour and modern cognition in human evolution.

## Materials and methods

### Radiocarbon dating

A total of 20 animal bone samples, including the pendant and the awl, were selected for radiocarbon dating. The collagen was extracted at the Department of Human Evolution, Max Planck Institute for Evolutionary Anthropology (MPI-EVA) in Leipzig (Germany) following the procedures in Talamo and Richards^19^ and Fewlass, et al.^15^ (MPI-Code: R-EVA).

The outer surface of the samples are first cleaned by a shot blaster and then 500 mg of the whole bones and c. 350 mg of the pendant and the awl were sampled. The samples are then decalcified in 0.5 M HCl until no CO_2_ effervescence is observed. 0.1 M NaOH is added for 30 min to remove humics. The NaOH step is followed by a final 0.5 M HCl step for 15 min. The resulting solid is gelatinised following Longin^31^ at pH 3 in a heater block at 75 °C for 20 h. The gelatin is then filtered in an Eeze-Filter™ (Elkay Laboratory Products (UK) Ltd.) to remove small (> 80 μm) particles. The gelatin is then ultrafiltered with Sartorius “VivaspinTurbo” ultrafilters (30 kDa MWCO)^32^. Prior to use, the filter is cleaned to remove carbon containing humectants^33^. The samples are lyophilised for 48 h. To supervise possible contamination introduced during the pretreatment stage, a pretreated ^14^C-free bone sample was used, kindly provided by the Oxford Radiocarbon Accelerator Unit (ORAU). Prior to sending the samples to the Mannheim facility for AMS dating (laboratory code MAMS)^34^, the collagen yield, C:N ratios, together with isotopic values are evaluated in order to understand the preservation of the collagen.

All the samples pretreated at the MPI-EVA passed the evaluation criteria (bones with > 1% weight collagen and C:N ratios in the range 2.9–3.6^35^) for good quality collagen (Table 1). The collagen of the pendant and the awl was split into two parts, one was sent to Mannheim AMS and the second one to the ETH Zürich (laboratory code, ETH), where the collagen extracts were graphitised using the AGE III^36^ and dated using the MICADAS^34,37^. The AMS measurements of the collagen backgrounds which were used in the age correction of all samples were highly reproducible within and between each magazine (~ 500 mg bone extractions: 2016 mean F14 C = 0.00168, s.d. = 0.00018; 2018 mean F14 C = 0.00220, s.d. = 0.00025). Due to the high reproducibility of the background measurements, extended measurement time, high rate of transmission and the use of the R_Combined of two separate dates, both the pendant and the awl, in Oxcal, we were able to reach exceptional levels of precision. An additional 1‰ was added to the error calculation of the samples, as per standard practice.

### Archaeological methods

The excavation was laid out using a 1 × 1 m grid system. The sedimentary sequence was excavated according to the natural stratigraphy. The position of the archaeological finds was recorded using a 3D coordinates system (see^38,39^). The excavated sediments were sieved using 2 mm and 4 mm mesh screens. The floated materials were separated for the recovery of micromammals, shattered bone fragments, lithic chips, and charcoals.

### Stajnia pendant analyses

Organic materials such as antler, bone and ivory can be distinguished by their micromorphological structure. In worked and especially polished objects, raw material identification is not always straightforward. Raw material identification of the Stajnia pendant was carried out by evaluating the broken edges and the exfoliated surface of the object around one of the perforations where the internal structure of the organic material was exposed. Mammoth tusk consists of a series of cones that are sequentially formed in the pulp cavity. These cones are made up of stacked dentine plates that, on macroscopic inspection, appear as milk-white homogeneous fibrous bands (e.g.^40,41^). Within these bands, microscopic canals 2 µm in diameter radiate outward from the pulp cavity^42^. These canals or dentinal tubules, in turn, are surrounded by collagen fibrils that coil up along the tubules^41^. The different orientations of the stacked radially distributed layers form the genus-specific distinctive patterns called 'Schreger lines' (see^42^ and references therein), which can be observed in transverse sections of larger tusk fragments. In this study, the material identification was based on the examination of the morphological features such as dentinal tubules and microlaminae that were visible on the broken edges of the object as well as on the exfoliated surface near one of the perforations (Fig. 2). The Stajnia pendant was analysed microscopically with a stereoscopic Olympus SZX9 microscope (magnification 6,3–57 ×) and metallographic microscope Nikon ECLIPSE LV100 (magnification 50–500 ×) at the Laboratory for Archaeological Conservation and Archaeometry Institute of Archaeology Wrocław University. The high-magnitude photographs were made with Environmental Scanning Electron Microscope Philips XL 30 ESEM/TMP at the Laboratory Scanning Microscopy (SEM)—Department of Geochemistry, Mineralogy and Petrology University of Silesia in Sosnowiec. The SEM analysis was used to examine the structure (including the analysis of the topography) of the surface of the object.

#### Virtual restoration of the Stajnia pendant

High-resolution µCT images of the two plaque fragments (S22222 and S23100) were obtained with an X-ray micro-computed tomography (XMT) scanner using the following scan parameters: voltage equal to 100 kV, currently equal to 0.062 mA, 1.0 mm Al filter, the reconstructed volume contains 1500 × 1500 × 1600 voxels. The data were segmented, and a three-dimentional isosurface of the external surface of the finds was created using Avizo Lite 2019.1 software (Thermo Fisher Scientific, Waltham, Massachusetts, USA)^43,44^. The 3D digital models obtained were then uploaded in Geomagic Design X (3D Systems, Rock Hill, South Carolina, USA) to carry out the optimisation of the surfaces (this process consists of cleaning and correcting defects to create fully closed surfaces)^45^. Subsequently, we proceeded with the virtual restoration of the Stajnia plaque. First, we proceeded with the interactive alignment of the two parts of the plaque, using the recognisable contact points as a reference. After obtaining an optimal alignment, the two fragments were joined, and the integration of the missing parts which formed cavities between the two original finds was carried out. Lastly, the photographic texture was applied using MeshLab 2020.03 software^46^.

#### NIR spectroscopy

Bone/ivory samples were scanned using a fiber-optic reflectance probe attached to a LabSpec 4 NIR spectrometer (Malvern Panalytical®) with a spectral range of 350 nm to 2500 nm. A Savitzky-Golay transformation (derivative order = 2; polynomial order = 3; smoothing points = 31) was performed to correct for additive and multiplicative effects in the spectral data using Unscrambler X software (Camo Analytics®). Partial least squares regression of data (wavelengths 1685–1740 nm and 2000–2300 nm) from specimens with known collagen yields was used to create a model predicting collagen content^16^. The resulting 3-factor model was used to predict % collagen in the unknown specimens. Because the model suggested collagen preservation in the specimens was very good (> 5% collagen yield) for samples of this antiquity, we were able to minimise the destruction of samples for subsequent analysis.

#### ZooMS

Zooarchaeology by mass spectrometry (ZooMS) analyses tissues rich in collagen type I and uses protein amino acid sequence variation to provide a taxonomic identification^47^. Both samples R-EVA 2650 (the awl) and R-EVA 2651 (the pendant) were analysed following ZooMS protocols which have been previously described in detail^47–49^. Collagen extracted for the radiocarbon dating process was used for ZooMS analysis. Each collagen sample was incubated into 100 µl of 50 mM of ammonium bicarbonate (Ambic) at 65 °C for 1 h, and 50 µl of the resulting supernatant was digested using trypsin (Promega) at 37 °C overnight. Samples were subsequently acidified using 1µL of 20% TFA, and peptide extracts were cleaned on C18 ZipTips (Thermo Scientific).

Each sample was spotted in triplicate on a MALDI Bruker plate with the addition of α-Cyano-4-hydroxycinnamic acid matrix. MALDI-TOF–MS analysis was conducted at the Fraunhofer IZI (Leipzig, Germany), using an autoflex speed LRF MALDI-TOF (Bruker) in reflector mode, positive polarity, matrix suppression up to 590 Da and collected in the mass-to-range 700–3500 m/z.

Triplicates were then merged for each sample, and taxonomic identifications were made through peptide marker mass identification in comparison to a database of peptide marker series for medium to larger sized mammalian species^48,50,51^.

## Supplementary Information

Supplementary Information.
