# Author Correction: A 41,500 year-old decorated ivory pendant from Stajnia Cave (Poland)

**DOI:** 10.1038/s41598-022-06348-8

**Published:** 2022-02-01

**Authors:** Sahra Talamo, Wioletta Nowaczewska, Andrea Picin, Antonino Vazzana, Marcin Binkowski, Marjolein D. Bosch, Silvia Cercatillo, Marcin Diakowski, Helen Fewlass, Adrian Marciszak, Dragana Paleček, Michael P. Richards, Christina M. Ryder, Virginie Sinet-Mathiot, Geoff M. Smith, Paweł Socha, Matt Sponheimer, Krzysztof Stefaniak, Frido Welker, Hanna Winter, Andrzej Wiśniewski, Marcin Żarski, Stefano Benazzi, Adam Nadachowski, Jean-Jacques Hublin

**Affiliations:** 1 Department of Human Evolution, Max Planck Institute for Evolutionary Anthropology, Deutscher Platz 6, 04103 Leipzig, Germany; 2 Department of Chemistry G. Ciamician, University of Bologna, Via Selmi 2, 40126 Bologna, Italy; 3 Department of Human Biology, University of Wrocław, ul. Przybyszewskiego 63, 51-148 Wrocław, Poland; 4 Department of Cultural Heritage, University of Bologna, Via degli Ariani 1, 48121 Ravenna, Italy; 5 X-Ray Microtomography Lab, Department of Biomedical Computer Systems, Institute of Computer Science, Faculty of Computer and Materials Science, University of Silesia, Będzińska 39, 41-200 Sosnowiec, Poland; 6 Vienna Institute for Archaeological Science, University of Vienna, Franz-Klein-Gasse 1, 1190 Vienna, Austria; 7 Turkana Basin Institute Ltd, Turkana, Kenya; 8 Turkana Basin Institute, Stony Brook University, N-507 Social and Behavioural Sciences, NY 11794-4364 Stony Brook, USA; 9 Department of Stone Age Archaeology, Institute of Archeology, University of Wrocław, Szewska 48, 50-139, Wrocław, Poland; 10 Department of Paleozoology, University of Wrocław, Sienkiewicza 21, 50-335 Wrocław, Poland; 11 Department of Archaeology, Simon Fraser University, Burnaby, BC V5A, 1S6 Canada; 12 Department of Anthropology, University of Colorado Boulder, Boulder, CO 80309 USA; 13 Centre for the Exploration of the Deep Human Journey, University of the Witwatersrand, Johannesburg, Gauteng South Africa; 14 Evolutionary Genomics Section, Globe Institute, University of Copenhagen, Copenhagen, Denmark; 15 Polish Geological Institute-National Research Institute, Rakowiecka 4, 00-975 Warsaw, Poland; 16 Institute of Systematics and Evolution of Animals, Polish Academy of Sciences, Sławkowska 17, 016 Kraków, Poland; 17 Collège de France, 11 Place Marcellin Berthelot, 75005 Paris, France

Correction to: *Scientific Reports* 10.1038/s41598-021-01221-6, published online 25 November 2021

The original version of this Article contained errors in the author list where Marjolein D. Bosch was omitted from the author list, and Mikołaj Urbanowski was incorrectly listed as an author of the original Article, and has subsequently been removed.

The Author contributions section now reads:

“S.T. W.N. and A.N. conceived the project; S.T., W.N., A.P., M.B., S.C., M.D., H.F., A.M., M.D. B., D.P., M.P.R., C.M.R., V.S-M., G.M.S., P.S., M.S., K.S., A.V., F.W., H.W., A.W., M.Z., S.B., A.N., J-J. H., performed research; S.T., A.P., W.N., M.B., M.D.B., S.C., M.D., H.F., A.M., D.P., M.P.R., C.M.R., V.S-M., G.M.S., P.S., M.S., K.S., A.V., F.W., H.W., A.W., M.Z., S.B., A.N., J-J. H. analysed all archaeological data; S.T. and A.P. wrote the paper with the collaboration of all the co-authors.”

The original Article and its accompanying Supplementary Information file have been corrected.

