# Supplementary material for: A 41,500 year-old decorated ivory pendant from Stajnia Cave (Poland)
Source: Sci Rep. 2021 Nov 25;11:22078. doi: 10.1038/s41598-021-01221-6 (PMC8626500; doi:10.1038/s41598-021-01221-6)
Supplement: Supplementary file 1 — Supplementary Information. [file 41598_2021_1221_MOESM1_ESM.docx]

**Supplementary Information for**

**A 41,500 year old decorated ivory pendant from Stajnia Cave (Poland)**

Sahra Talamo, Wioletta Nowaczewska, Andrea Picin, Antonino Vazzana, Marcin Binkowski, Marjolein D. Bosch, Silvia Cercatillo, Marcin Diakowski, Helen Fewlass, Adrian Marciszak, Dragana Paleček, Michael P. Richards, Christina M. Ryder, Virginie Sinet-Mathiot, Geoff M. Smith, Paweł Socha, Matt Sponheimer, Krzysztof Stefaniak, Frido Welker, Hanna Winter, Andrzej Wiśniewski, Marcin Żarski, Stefano Benazzi, Adam Nadachowski, Jean-Jacques Hublin

Corresponding author: **Sahra Talamo,** Department of Human Evolution, Max Planck Institute for Evolutionary Anthropology, Deutscher Platz 6, D-04103 Leipzig, Germany; Department of Chemistry G. Ciamician, University of Bologna, Via Selmi 2, 40126 Bologna, Italy.

**Email**: [sahra.talamo@unibo.it](mailto:sahra.talamo@unibo.it)

**This PDF file includes:**

Supplementary text 1 to 8

Figures S1 to S15

Tables S1 to S15

SI References

**Section 1 – Stratigraphic background of Stajnia Cave**

*Marcin Żarski*

Stajnia Cave (50°36’58"N, 19°29’04"E) is located on the northern side of the Mirów Elevation at an altitude of 359 m a.s.l. between the villages of Mirów and Bobolice in the Kraków-Częstochowa Upland (Poland). The cave is a rocky elevation on the Upper Oxfordian massive limestone with a narrow morphology (length of ~23 m, width of ~2-4 m, and height ~6 m)^1^ (Fig. S1). Archaeological fieldwork at the site was carried out between 2007 and 2010, and the excavation covered an extension of ~16 m^2^ in the rear of the cave. In 2008 and 2009, geophysical analysis by resistivity-tomography indicated that the thickness of sediments at the centre of the cave might reach ~6-7 m^1^.

Stratigraphy

The sedimentary sequence of Stajnia Cave is composed of 15 lithostratigraphic layers dated from the Early Glacial (MIS 5c) to the Holocene and represented by cave loams containing limestone, rubble, sand, silt, and clay^1^ (Fig. S1). The stratigraphy is complicated due to post-depositional frost disturbances, partial sediment sinking, and modern distortions (Fig. S1). At the top is unit A, composed of a Holocene humus horizon with limestone rubble mostly removed during late Medieval times. In some areas of the cave, the humus horizon is heavily reworked and classified as unit SH. Below unit A lies unit B, composed of two layers (B6 and B7). Layer B6 is characterized by yellow sandy cave loams with a great amount of limestone rubble and sandy lenses. The gravel fraction is dominant among the other fractions (sand (26.1%, the average value for the layer), clay (19.8%) and silt (6.1%)). Below is layer B7 described as yellow, silty-sandy cave loam with limestone rubble and sandy lenses (levels: 120 cm – 65 cm;). The silty fraction (67%) is more abundant than sand (20.1%), clay (8.1%) and gravel (4.8%).

The next unit is unit C which includes several layers. On top is layer C6 (levels: 140 cm - 70 cm) composed of yellow sandy cave loams with rubble. This layer is found generally below layer A and, in some areas, below layer B6. The granulometry of layer C6 is fairly uniform in vertical profile and consists of sand (36.9%), gravel (25.9%), clay (19.7%) and silt (17.5%). In this layer, slightly rounded rubble (82.5%), sharp-edged rubble (17.5%), and single pieces of rubble with pits and manganese-irony inclusions on its surface are also observed. Below is layer C7 located close to the cave wall (levels: 130 cm - 85 cm). This layer is discontinuous and consists of brown-yellow cave loams, including fractions of sand (more than 40%), silt (more than 20%), clay (about 20%) and gravel (over 10%). The content of the humus in this layer is 0.74%. Layer C18 lies on complex D and is discontinuous (levels: 130 cm – 95 cm). Layer C18 consists of light-grey, silty-sandy cave loams with limestone rubble. The silty fraction (45.1%) is prevalent over other types of fractions (including sandy (34.9%), clay (10.5%) and gravel (9.5%)) characteristic of this layer. The slightly-rounded limestone rubble with a high content of the sharp-edged rubble is prevalent in this layer. The discontinuous layer C19 lies on layer D1 (levels: 120 cm – 105 cm). The granulometry composition consists of grey cave loams with red streaks and limestone rubble. The following types of fractions are distinguished: sand (about 39.1%), silt (21%), clay (17.8%) and gravel (23.4%). Slightly-rounded rubble with pits of chemical etching and sharp-edged rubble with different surfaces can be observed in this layer.

Unit D includes four layers (D1, D2b, D2 and D3) characterized by the highest content of organic matter including numerous faunal and lithic remains. The average thickness of this unit is about 50 cm. During the excavation, four Neanderthal teeth were discovered in layer D1, and another three in layer D2. The granulometry of layer D1 consists of light-brown, sandy-clayey-silty cave loams with rubble (levels: 180 cm – 95 cm). Some differences in the proportions of the granulometric composition in each level have been observed. The sandy fraction (38.7%) is dominant among the other distinguished types of fractions (clay (17.6%), silty (16.1%) and gravel (16.3%)). Slightly rounded rubble (60%) with an admixture of the rounded rubble (13%), with magnesium-phosphorus precipitates in some areas and a slightly higher content of sharp-edged rubble (27%), prevails in this layer. The local limestones (from 43.8% to 63.8% of the content) are prevalent in the mineral composition of the sandy fraction. The layer D1 contains quartz (from 25.5% to 48.6%) and faunal remains (from 1.5% to 17.8%). The content of humus in this layer is high (1.2%). Layer D2b was probably formed during the deposition of layer D2 and has a discontinuous structure. In terms of granulometry, layer D2b consists of light-brown sandy cave loams with limestone rubble (levels: 190 cm – 175 cm). This layer includes the following types of fractions: sand (from 71% to 49%), silt (from 9% to 18%), clay (from 17% to 21%) and gravel (from 3% to 9%). Among limestone rubble, the slightly-rounded rubble (79%) is prevalent over both rounded rubble (10%) and sharp-edged rubble (11%). Corrosive pits and magnesium-phosphorus precipitates on the surface of the rubble have also been observed.

Below layer D1 is located layer D2 composed of light-brown sandy-clayey cave loams with rubble (levels: 220 cm - 155 cm). The granulometric analyses were not conducted for this layer. The content of the humus in that layer is high (1.3%). The D2b structure is observed in the ceiling of layer D2 and its geological characteristic is similar to that established for layer D2.

Layer D3 is of moderate thickness (about 30 cm) and is located at the base of layer D2 and above layer E1 - described at some places as layer E (levels: 210 cm – 190 cm). Layer D3 consists of dark-brown, sandy-clayey cave loams with an admixture of fine limestone rubble. The sandy fraction is prevalent (its average percentage of the content of this layer is 38.2%) over the other types of fractions (fraction clay - 24.9 % and silty - 23.1%).

Unit E underlying complex D consists of two layers: E1 (archaeologically sterile) and E2 (with a few Late Middle Palaeolithic flint artefacts). The presence of a great amount of large limestone rubble is characteristic of these layers (levels: from 130 cm to 250 cm). Layer E2 underlies layer E1. In some places, where both layers (E1, E2) are not distinguished, they were described as layer E. Layer E1 consists of a great amount of limestone rubble and its thickness is about 50 cm (levels: 225 cm – 110 cm). The results of the granulometry analysis indicated that 71.3% of this layer consists of rubble. In terms of granulometry, layer E1 is composed of light-grey, sandy-silty cave loams with a significant amount of limestone rubble. Within this layer, the percentages of the different types of fraction changes significantly (clay fraction (10% - 25%), silty fraction (7% - 44%) and sandy fraction (14% - 75%)). The slightly-rounded rubble (88%) with an admixture of the rounded and sharp-edged rubble is prevalent whereas rubble with corrosive pits and magnesium-phosphorus precipitates on the surface is also present. The dominance of the limestones (more than 55%) over quartzes (more than 30%) in the mineral content of the sandy fraction of layer E1 has been observed. The content of humus in that layer is not high - 0.89%. In terms of granulometry, layer E2 consists of brown-grey (at some places black) silty cave loams with significant rubble. It includes the following types of fractions: silty and clay (50%), sandy (from 23% to 37.7%) and gravel (from 5% to 8.8%). Dominance of the limestones (more than 60%) over quartzes (more than 30%) in the mineral content of this layer has been observed.

Below unit E is layer F (levels: from 250 cm to 130 cm) composed of light-grey to brown sandy cave loams. The sandy fraction (more than 40% of the layer content) is prevalent over the two other types: silty (about 30%) and clay (about 15%). Numerous black calcium-phosphate concretions among the limestone rubble are also documented. The rubble from this layer is characterized by its rounded edges. The content of humus is low (0.43% and 0.53%).

At the bottom is layer G (levels: from 250 cm to 130 cm). Due to the discontinuities of the upper layers in some areas close to the walls of the cave, layer G is also located under layers E1, E2 and D1. This layer consists of orange-brown sandy cave loam with a small amount of limestone rubble. The sandy fraction is dominant over silt and clay, and limestone prevails over quartzes in the mineral content.

Geological analyses

The average percentage of the clay fraction (0.01-0.001 mm) in layers distinguished in Stajnia Cave varies from 18-19%. The lowest values of the clay fraction are recorded in layers B6 and C18 – 8.1 and 10.5% respectively. The highest values are recorded in layers D3 - 24.9% and G -19.1%. The clay fraction arises mainly as a result of chemical weathering, which depends mainly on humidity and temperature. At higher temperatures, there is a larger amount of chemical weathering. Differences in values of the clay fraction are probably linked with small temperature variations. Layers B7 and C18 may have been deposited in colder temperatures than others. The percentage of the silty fraction (0.1 - 0.01 mm) in each layer varies from 6.1% in layer B6 and 13.8% in layer D2b to 67% and 45.1% in layers B7 and C18 and 34.5% in layer E2. In all other layers, the average is more than 30%. A large supply of dust was deposited in periglacial conditions during the cold climate. It correlates with greater values of silt fraction in these layers. The smallest fraction of silt content is observed in layers C6, D2 and D2b, and is connected with ground vegetation cover and a warmer climate. The percentage of the sandy fraction (2-0.1 mm) between layers varies from 20.1%-20.6% up to 60.1% in layer D2b. A greater proportion of the sandy fraction may suggest a slightly wetter environment and water transport. Sand can also be transported by C processes.

Analyses of the morphology of rubble limestone layers were carried out in layers C6, C18, D1, D2b, E1, and F2 in two fractions: 10 mm - 20 mm and 20 mm. Slightly rounded rubble prevails in the studied samples of limestone. Layers D1, D2b and E1 have an admixture of fine rounded limestone rubble of about 10%. This indicates a slightly warmer climate during the accumulation of these layers compared to the rest. The highest percentage of sharp-edged limestone rubble is observed in layer C18 - above 40%. The limestone rubble from these layers is covered with precipitations of phosphorus and iron and manganese oxides. Manganese is a result of weathering of dark minerals such as amphiboles, pyroxenes and biotite in humid and warm climatic conditions. In layer F2, limestone rubble is characterized by a porous surface indicating chemical weathering. In the mineral-petrographic composition, local limestone rocks dominate as a product of weathering, ranging from 70% in layer G to 54.6% in layer D2. The content of quartz grains ranges from 28% in layer G to 34.8% in layer D2. Quartz comes from outside the cave and was transported by water or aeolian processes. The admixture of crystalline rocks does not exceed 2%. Manganese-iron concretions are very scarce with the exception of layer E1- 3%. A significant admixture of animal remains is found in layers D2 and E1 (8.7% and 3%).

In all layers except layer G, rounded matt and half-matt (RM and EM/RM) quartz grains dominate (70%), indicating strong aeolian processes in dry and cold climatic conditions. In layer G, aeolian quartz grains only made up 30%. In this layer (52.1%) polished grains characteristic of fluvial sedimentation predominate, which accumulate in warm climates. The content of polished, intermediately-rounded, glossy quartz grains reaches 20% in the rest of the layers. The content of broken grains (C) is about 10% in all layers. These grains are characteristic of mechanical weathering in a cold climate (frost processes). Based on studies of the quartz grain surfaces, it can be concluded that the sediments in the investigated layers were accumulated in a cold and dry climate.

The content of transparent (allochtonic) minerals in the heavy fraction is 28.3% in layer G, increasing to 67.3% and 58.8% in layers D2 and D2b. Heavy minerals mainly come from the outside as a result of weathering of sediments. Layers D2 to G are marked by a decreasing content of transparent minerals, which may be associated with the processes of chemical weathering in warmer climates. The decline of transparent heavy minerals is noted in layers C7 and B7. The content of transparent mineral in layer C6 is 78.4%, indicating deposition from outside the cave in a cold climate.

The composition of heavy minerals is predominated by non-transparent minerals, mainly carbonates (ca. 40%) in situ resulting from the dissolution of limestone rocks. The increasing content of carbonates are from Layer D3 to layer F may suggest wetter conditions of sedimentation. The carbonate content in layer C6 is not recorded. The contents of primary and secondary oxides are approximately 10%. Garnet is dominant among transparent minerals, as it is resistant to mechanical damage but less resistant to chemical weathering. The content of garnet in layer C6 is 44.4%. Beginning in layer D2b, garnet content declines (in layer F it is 4.8%). The decrease in garnet content in these layers indicates chemical weathering in warm climates. Low garnet content (16%) is also recorded in layer B7. In all layers, there are traces of the minerals least resistant to weathering: glauconite, biotite, pyroxene and muscovite. Amphibole, which has weak chemical weathering-resistance, represents a small percentage. It is noted that small fluctuations of amphibole content in layers may indicate small temperature fluctuations. The highest amphibole content is observed in layer C6 - 10.4%. Higher amphibole content may indicate cold climate conditions. The lowest amphibole content is observed in layer E2 - 1.6% and layers D3 and C7 - 2.3%. The lower amphibole content may indicate more intense chemical weathering in warmer climates.

Among the heavy minerals, a significant portion is very resistant to weathering. These are zirconium (2-6%), tourmaline (2-6.7%), topaz (1-6%) and staurolite (7-10%). These minerals, together with the garnet minerals, constitute the vast majority of the transparent minerals. A large amount of very weathering-resistant minerals indicates redeposition of mainly sandy sediments before accumulation in the cave.

Calcium dominates the main elements, reaching an average of 50% from a group of distinguished elements. The highest value of calcium is noted in layer B6 - 85% - and the lowest in layer E2 - 37%. Calcium dissolves easily in water. Low calcium content may correspond to drier climatic conditions. The aluminium content ranges from 6% in layers B6 to 27% in layer E2. The average aluminium content in the other layers is about 17%. Aluminium mainly originates from outside the cave. Little variation in aluminium content values between the layers indicates similar sedimentation conditions. Phosphorus accounts for a significant percentage of the group of main elements. Phosphorus most likely originates from the destruction of skeletal remains. The highest phosphorus content is observed in layers F and G - 20.6% and 19.2%. The lowest percentage of phosphorus is observed in B6 and C6, layers D3 and C7 - 2.3% and 2% respectively - and in layer C7 - 5%. The phosphorus content in layer E2 is 11% and is lower than in the E1 layer - 16%. Similar phosphorus values are recorded in layer D2. Increased phosphorus content in the sediments is probably associated with stronger chemical weathering in warmer climates. The iron content in the cave sediments in the group of selected elements ranges from 2.81% in layer B6 and 11.2% in layer C7. The content of iron in the other layers is about 8-9%. The content of Fe in cave loams is higher than in the surrounding rocks. Iron enters the cave through water and aeolian transport. The potassium content ranges from 4% in layers G and F to 9.8% in layer C6. The potassium content in other layers is 6-7%. Potassium comes from the weathering of rocks and the feldspar and mica content originates from outside the cave from Pleistocene sediments. Small fluctuations in potassium show similar sedimentation conditions represented in each layer. Sodium content is about 2% in selected layers in the cave. Only layers C2 and B6 contain 0.8% and 1.5% sodium respectively. Sodium is also formed by the weathering of feldspars. The magnesium content present in each layer is approximately 1%. The magnesium content in layer C6 is 3% and is 2.2% in layer C7. The lowest magnesium content is 0.6% in layer B6. Magnesium comes from the leaching of limestone in an environment of high humidity.

Trace elements are present in the cave sediments in minute quantities - 500-3500 ppm. Titanium is produced by the mechanical weathering of minerals, mainly iron. The highest percentages of titanium are recorded in layers C6 - 62.3%, B6 - 53.3%, C7 - 50.5% and E2 - 42.5%. The lowest percentage of titanium is present in layer F - 20.4%. The titanium content in the remaining layers is about 30%. Larger quantities of titanium may indicate increased mechanical weathering in cool climates. Manganese is the result of chemical weathering of limestone rocks in warm climates. The largest share of manganese is observed in layer F - 55% and layer G - 39%. The lowest manganese content is recorded in layers B6 - 15.8%, C6 - 13.8% and C7 - 19%, and is evidence of cold climatic conditions. The content of manganese in the remaining layers is about 30%. Barium content in all layers is similar and is approximately 10%. The content of zinc in different layers is variable and ranges from 5.3% in layer C6 and 8.6% in layer C7. The zinc content is 20% in layer B6 and 19% in layer C9. Zinc content in the other layers varies between 10% and 15%. Smaller amounts of Zn may indicate drier climate conditions and larger amounts indicate wetter climatic conditions.

The highest carbonate content is observed in layer C9 - about 12%. In layers D2, D2b, D3, and E1, carbonate content ranges from 4-6% and in layers E2, F and G it exceeds 4%. Low carbonate levels are connected with relatively moist climates. The content of organic matter is low, less than 1% in most of the cave layers. The organic matter content is only bigger than 1% in layers D1 and D2, probably as a result of more recurrent human occupations.

Laboratory analyses confirmed differences in the types of sediments and their mineral and lithological characteristics due to climatic fluctuations during the accumulation of sediments in every layer. Sediments of layer A were accumulated in the Holocene and correlate with MIS 1. Sediments in layers B6 and B7 were deposited in cool climates and could correlate with the Late Pleniglacial or the Late Glacial (MIS 2 or MIS 2/1). Sediments in layers C6, C7 and C18 were accumulated in colder climates compared with other layers and the age of these layers suggest a correlation with the Late Pleniglacial (MIS 2) (Table 1). The sediments of layer C19 were deposited in a slightly warmer, but still cool climate in the Middle Pleniglacial (MIS 3). The sediments of layer D1 were accumulated under similar climatic conditions to those of layer C19 and correlate with the Middle Pleniglacial (MIS 3). Slightly warmer climatic conditions prevailed during the accumulation of sediments in layers D2b, D2 and D3 and the age of these layers can be correlated with the Middle Pleniglacial (MIS 3). In fact, in layer D2b, the dating of two mammoth tooth fragments by U-Th series yielded ages of ~52,900 BP, and a radiocarbon date on an ungulate bone is >49,000 BP. In layer D3, two fragmented bones of a bear and an ungulate are >49,000 BP (Table 1). The sediment of layer E1 suggests a cold and harsh environment and is associated with the Early Pleniglacial (MIS 4), with a radiocarbon date on an ungulate bone from this layer >49,000 BP (Table 1). The sandy-silty loam accumulation of layer E2 reveals warmer climatic conditions possibly connected with the Odderade Interstadial (MIS 5a). Sediments in unit F could have been accumulated in slightly cooler climates during the Rederstall Stadial (MIS 5b). Sediments in unit G were accumulated in warmer climates than deposits in layer F and can be correlated with the Brørup Interstadial (MIS 5c) (see also^1^ and Table 1).

**Section 2 – Pollen Analyses**

*Hanna Winter*

The study of the pollen of Stajnia Cave was carried on the sediments from layers D2, D2b/D, D2b, D1, C19, C18, C7 and C6 (Table S1). The samples analysed yielded 8,092 pollen grains, of which 7,547 could be identified at the rank of family, genus, or species (Table S1). Laboratory sample preparation included calcium carbonate dissolution in 10% HCl. Next, the sediment was boiled in 7% KOH, and the separation of the mineral fraction was made with the use of heavy liquid (water solution of cadmium iodide and potassium iodide, density c. 2.1 g/cm3). The maceration was conducted according to the modified acetolysis procedure after Erdtman^2^. The examination revealed different frequencies and various degrees of preservations. Heavily destroyed and, thus unidentifiable grains, are present in every sample. Some pollen could be identified only at the rank of family. Poor grain preservation is typical for cave sequences, and the pollen spectra from Stajnia are relatively well preserved and rich in comparison to other caves from the Częstochowa Upland^1^.

The spectra are dominated by pollen of NAP (dwarf shrubs and herbaceous plants), represented mostly by Cichorioideae, whereas other pollen such as *Helianthemum*, *Anthemis* t., Asteraceae, Apiaceae, Poaceae, Caryophyllaceae, *Cirsium* t., Brassicaceae, *Artemisia*, Ericaceae, Cheonopodiaceae are present at a lesser percentage. The tree pollen includes *Betula* and *Pinus sylvestris* t. while pollen of other tree species occurred sporadically. Polypodiaceae spores were relatively frequent. This data indicates that the spectra from the studied archaeological layers are characterised by strong domination of NAP, which suggests an open environment^1^ (Fig. S2). This interpretation is supported by the presence of *Helianthemum* and *Artemisia*, common in nearly all samples. *Helianthemum* is typically a heliophilous genus, growing in full sunlight. *Heliannthemum nummularia* grows on dry soils, but other varieties could appear on soils with different degrees of moisture. Since the species of *Helianthemum* is entomophilous (self-pollinating), it produces a low amount of pollen and generally is underrepresented in the pollen spectra^3^.

Several pollen types such as Cichorioideae, *Cirsium* t., Brassicaceae and *Thalictrums* represent humid conditions. The grassland biotopes were a source of pollen of *Potentilla* t., *Galium* t., Apiaceae, and *Bupleurum falcatum* t. (Table S1). Steppe flora is represented by *Artemisia*, Poaceae, Chenopodiaceae, *Helianthemum nummularia* t., Asteraceae, Centaurea (Table S1). The presence of Ericaceae, *Polygonum bistorta* t., *P*. *aviculare* t., *Polemonium*, *Valeriana*, *Plantago* *media*, *Ranunculus acris* t. is an indicator of meadow-tundra vegetation. The Arctic and alpine-type flora is represented by *Saxifraga hirculus* t., *S*. *oppositifolia* t., *S*. *stellaris* t., *Polygonum bistora* t. and *Selaginella selaginoides* (Table S1).

Precise palaeoenvironmental reconstruction is difficult due to the limited value of pollen data. The high percentage of NAP pollen, caused by the Cichorioideae overrepresentation, biases the AP to NAP ratio, which could be an indicator of a forested environment (Table S1). Although open landscape species dominate in the pollen record, it is worth noting the relatively high percentage of *Betula*, whose grains are relatively heavy, suggesting the presence of trees in the direct vicinity of the cave during the deposition of most of the analysed units. The presence of *Pinus* pollen is relatively low and could have transported from a more distant source. However, some remains of pinewood were identified during the charcoal analysis.

In conclusion, palynological data from Stajnia cave reflect the local environment, precluding a broader vegetal reconstruction at the regional or micro-regional level. However, the analysis indicates that during the deposition of sediments the climatic conditions were relatively cold with a continental climate influence. The continental climate influence systematically increased starting from unit D2 and continuing in units C19 and C18, which document the most severe conditions. These data are consistent with the palynological record of trees, the shrub presence, and the geological observations (SI Section 1, see also^1^).

**Section 3 – The faunal assemblage**

*Adrian Marciszak, Paweł Socha, Krzysztof Stefaniak, Adam Nadachowski*

The detailed zooarchaeological study of the total faunal assemblage is published in^4^. It is worth noting that the larger faunal remains consist of more than 31,000 determined teeth and bones of small mammals, reptiles and amphibians. Only 13,500 bones, bone fragments and teeth belong to larger mammals and birds. At least 26 species belong to carnivores (Carnivora) and herbivores (Artiodactyla, Perissodactyla and Proboscidea) compose the larger mammal assemblage (details in the SI in^4^). Most of the identified large mammals were found in the archaeological levels of unit D and older layers of unit C. The *Mammuthus-Coelodonta* Complex is represented by nine different species and reindeer is the most abundant species. The carnivore paleocommunity from Stajnia Cave consists of 18 species (in Table S9 of Picin, et al.^4^). The bone assemblage is highly fragmented. Among the three canids present, the most frequent is *Vulpes vulpes* (red fox) whereas *Canis lupus* (wolf) and *Vulpes lagopus* (Polar fox) are documented in a lesser percentage. Bear remains are mostly assigned to *Ursus spelaeus ingressus*, mostly present in units D and C. A typical steppe species, the steppe polecat (*Mustela eversmanii*) is documented with a great number of stoat (*Mustela erminea*) and weasel (*Mustela nivalis*) remains. The avifauna is dominated by the genus *Lagopus* (*L*. *lagopus* and less frequently *L*. *muta*) as well as corvids, especially *Corvus monedula*, and the genus *Tetrao* with almost 600 bone remains.

**Section 4 – The lithic assemblage**

*Andrea Picin and Andrzej Wiśniewski*

The lithic assemblage of Stajnia Cave is composed of 545 lithic items (Tables S2 and S3). The raw material used for the knapping activities is Jurassic flint, gathered from outcrops located in the neighbourhood of the site. Macroscopic examination of the assemblage reveals differing preservation of the lithic artefacts. Several items are rounded and heavily patinated while others show fresher edges and lighter desilication patina on the surfaces. This pattern is present throughout the sequence with no clear differences between layers. Moreover, the reconstruction of the *chaîne opératoires* indicates a mixing of artefacts from different chronologies suggesting that post-depositional frost disturbances and modern distortions displaced the lithic items between different levels. Because of these taphonomic conditions, the assemblage is analysed as a whole, and the stratigraphic subdivision should be considered cautiously.

The technological analysis indicates that most of the artefacts could be attributed to the Middle Palaeolithic and, in particular, to the Central European Micoquian whereas the stone tools ascribed to the Upper Palaeolithic are few (Tables S2 and S3). The number of cortical flakes indicates that knapping activities were generally carried out outside the cave, and the artefacts were transported to the site as components of the toolkit. The examination of the core collection shows that the main strategy of reduction is centripetal using the discoid and the hierarchized centripetal methods (Table S3). Although these reduction strategies are distinctive of the Middle Palaeolithic, several examples are also found in units C (MIS 2-1), and in the dump. The examination of the lithic by-products shows the presence of centripetal flakes and artefacts associated with the maintenance of the core convexities such as core-edge removal flakes and pseudo-Levallois points (Table S2).

The use of Levallois technology is attested by one recurrent centripetal core in layer D2 whereas the other examples documented in layers D1 and D3 are heavily rounded, making the identification of the modality difficult. Some Levallois flakes are found in layers C18, D1 and D2, and were produced using the modalities recurrent unidirectional, bidirectional and centripetal (Tables S2 and S3). The remaining artefacts of the secondary operative chains are hierarchized bidirectional, simple unidirectional and polyhedral cores. These reduction sequences were aimed at the production of ordinary flakes of different sizes. A refitting between a hierarchized core and a centripetal flake is found in layer C7.

The assemblage of retouched artefacts includes scrapers and notched tools (Table S2). It is worth noting the presence of three exhausted bifacial tools, a fragment of a leaf-shape point, and few bifacial preforms (Table S2). Some flakes show the occurrence of lipped platforms, a feature associated with the use of a soft hammer and bifacial shaping. In the collection, a scraper on a cortical blank with a Quina scaled retouch and one *groszak* were also found. Moreover, a point with inverse retouch and hinge fracture near the tip stand out (Fig. S3 n°5). Although the lithic assemblage of Stajnia Cave is highly fragmented and bifacial backed knives (*Keilmesser*) are missing, the presence of bifacial tools, leaf point and *groszak* supports the association with the Central European Micoquian (see^4^).

In comparison with the Micoquian stone tools, the amount of Upper Palaeolithic artefacts is smaller and comprises only 66 lithic items, of which nearly half are broken (Tables S2 and S3). The high fragmentation of the *chaîne opératoires*, the absence of diagnostic stone tools and the post-depositional processes that displaced the artefacts between the different layers makes the cultural discrimination of the laminar and lamellar by-products difficult. The results of the radiocarbon dating indicate human occupations during the Early Aurignacian and Magdalenian (Table 1 and SI Section 7). In order to discriminate between the artefacts, the attribution to the Early Aurignacian or Magdalenian is based on the technological features of the by-products.

In the assemblage, three cores were found (Table S3). A lamellar core, discovered in layer E2, is characterized by preparation of the striking platform with two unidirectional removals, bidirectional detachments on the flaking surface, and preparation of a crest on the backside of the core (Figure S3 n° 4). The second prismatic lamellar core, found in layer D1, is probably made on a cortical flake or a chunk (Figure S3 n° 7). The striking platform is prepared with a unidirectional detachment whereas the flaking surface is narrowed by a lateral removal from the mesial-distal side. A hinged knapping accident occurred during the bladelet production and the core was discarded. The latter bladelet core was found in the dump and out of context (Figure S3 n° 6). The striking platform is prepared with two orthogonal removals whereas the flaking surface is shaped in a triangular morphology by two invasive detachments. During the production, a knapping accident removed a portion of the flaking surface leaving evident only one bladelet removal. Although in the Western Early Aurignacian lamellar cores are mostly carinated^5-7^, similar approaches to the examples of Stajnia in the shaping of the core convexities and the abrupt unidirectional bladelet production occur at the Aurignacian sites of Księcia Józefa layer II^8^ and Piekary II layer 6^9,10^. Small prismatic cores are also documented at Geißenklösterle AH III ^11^. On the other hand, the three cores are missing the careful preparation of the striking platforms (e.g. *en éperon* technique) which is common in artefacts of the late Magdalenian^12-14^.

In the assemblage, by-products attesting the management of the core convexities includes only two core-rejuvenation flakes (core tablets) found in the dump (Table S2). Ordinary blades are common whereas bladelets are few (Table S2). Unfortunately, retouched bladelets are missing, impeding a more detailed cultural attribution. After a broad comparison with other Polish Upper Palaeolithic collections, a large semi-cortical blade, a refitted Aurignacian blade, a crested blade, and three blade fragments with plain platforms are attributed to the Early Aurignacian (Fig. S3 n° 1, 3; Fig. S4 n° 1, 6, 7, 14, 15). Conversely, three blades with lipped platforms, typical of soft hammer percussion, and a bladelet fragment are associated to the Magdalenian^15^ (Fig. S3 n° 2; Fig. 4 n°4, 5, 16). The remaining artefacts are regrettably broken or undiagnostic, making their chronological placement difficult.

In the assemblage, an endscraper, a dihedral burin on a core-edge blade, and probably a dihedral burin on a blade were also found (Table S2). In Central Europe, burins occur together with endscrapers in the Early Aurignacian assemblage of Geißenklösterle AH III^11^ whereas in Poland they are found at the Aurignacian site of Kraków-Zwierzyniec^16^. From this perspective, the large dihedral burin made on a core-edge blade (Fig. S4 n° 18) is tentatively attributed to the Aurignacian although the association with the Magdalenian could not be excluded^17^. Conversely, the other burin is characterized by a curved lateral removal that could also be produced after a fracture (Fig. S4 n° 22).

Contextualizing the lithic assemblage of Stajnia Cave

In Poland, the period between 50-40 ka BP was characterized by the succession and overlapping of different cultural entities that have no comparison with the other European regions. These archaeological evidences are scattered in rather small areas ranging between the neighbourhoods of the city of Kraków, the Kraków-Częstochowa Upland, the Western Carpathian Mountains, Lublin, and Lower Silesia^18-21^. During the final Middle Palaeolithic, concurrent with the Central European Micoquian, three transitional industries are documented: the Szeletian, the Jerzmanowician and the Zwierzyniecian^22-26^. While the Szeletian and the Jerzmanowician are renewed techno-complexes, the Zwierzyniecian is generally less known in the European panorama. This industry is dated approximately 40 ka BP at Kraków-Zwierzyniec site 1, and is composed of arch-backed points that morphologically are similar to the Uluzzian lunates and Châtelperronian points^23,26^. Similar artefacts are also found at Mamutowa Cave and Obłazowa Cave^23,26^.

The beginning of the Upper Palaeolithic is also heterogeneous and divided into three main Aurignacian variants: 1) the Zwierzyniec Type - characterized by higher percentages of burins over endscrapers and retouched tools; 2) the Piekary Type - characterized by higher percentages of endscrapers over burins; 3) the Góra Puławska Type - typified by the higher amount of end-scrapers, Dufour bladelets and fewer burins^21,27^. Following this typological subdivision, Góra Puławska was associated to the Protoaurignacian (Krems-Dufour facies), and the Zwierzyniec and Piekary variants were related to the Typical Aurignacian (nowadays known as Late or Recent Aurignacian)^21,27^. Recently, the assemblage of Góra Puławska II has been ascribed to a new variant of the Evolved Aurignacian named Góra Puławska-type^28^ whereas the industries of Księcia Józefa layer II and Piekary IIa level 6 have been attributed to a non-Aurignacian UP rooted in the preceding Middle Palaeolithic record^29^. This latter interpretation disagrees with previous work at Piekary IIE where diagnostic Aurignacian cores and stone tools were documented^9^.

In the last decade, several works tried to shed light on this crowded succession of cultural entities but the investigations yielded contradictory results. The Szeletian occurred at Obłazowa Cave between 45-39 ka cal BP^30^, and Lubotyń 11 at some point between 49‐39 ka cal BP^31^. On the other hand, the chronological reassessment of layers VI-V of Nietoperzowa Cave estimates a range between 44-42 and ~38 ka cal BP for the Jerzmanowician whereas layer IV, dated ~30-31 ka cal BP, is instead associated with the Early Gravettian^32^. At Koziarnia Cave, new fieldworks point out that the Jerzmanowician is dated between 46-41 ka cal BP and the Early Gravettian occurred in two distinct events at c. 37-35.5 ka cal BP and c. 32-28.5 ka cal BP^33^. At Księcia Józefa layer II, a UP lamellar technology (non-Aurignacian) is dated c. 40 ka cal BP whereas at Piekary IIa layer 6 an undetermined UP industry is found at 32-26 ka cal BP^29^. Conversely, the beginning of the Aurignacian is mostly based on the typological description of lithic artefacts from Kraków-Zwierzyniec suggesting an age of c. 35 ka cal BP^16^ and on the direct dates of two Mladeč points from Mamutowa Cave, a typical element of the Recent Aurignacian, yielding a range between 38-35 ka cal BP^34^. These results reveal a large contemporaneity of different cultural traditions in a relatively small area, a scenario that does not occur in the neighbouring regions. This situation could be attributed to different factors: large chronological uncertainty, conventional chronometric techniques, and/or poor sample selection for radiocarbon dating.

Recent works in Stajnia Cave showed that post-depositional processes have severely affected the sedimentary sequences of the cave, moving artefacts and human remains between levels^4^. This issue could also have been common in other cave sites of the Kraków-Częstochowa Upland and the Western Carpathian Mountains because of the frequent climatic oscillations during the Late Pleistocene and the relative proximity of the Scandinavian ice sheet during the Last Glacial Maximum^35-37^. Furthermore, solifluction processes could have reworked the sediments in open-air sites, mixing lithic by-products from different chronologies. In this complicated context, the discrimination of the occupation palimpsests is problematic and the chronological determinations could have been severely biased.

The reconstruction of the technological strategies used in Stajnia Cave reveals a high fragmentation of the *chaîne opératoires* with few knapping activities carried out inside the natural shelter (Tables S2 and S3). These characteristics are typical of short-term occupations when the site is used as a logistical location during forays^38,39^. During the Middle Palaeolithic, similar mobility tactics are recognized in many Micoquian caves and open-air sites of Central Europe^40-44^. Low density finds are also documented in the Szeletian layer of Lubotyń 11^24^, in the Szeletian and Aurignacian layers of Obłazowa Cave^30,45^, in the Aurignacian of Kraków-Spadzista layer 7^46,47^, Deszczowa Cave layer VIIa^48^, Piekary IIa layer 6^9,10^ and in the Upper Palaeolithic occupation of Księcia Józefa layer II^8^. Unfortunately, most of the sites preserving Jerzmanowician and Zwierzyniecian assemblages, including Mamutowa Cave^49,50^, Nietoperzowa Cave and Koziarnia Cave^22^, were excavated during the late XIX/early XX centuries with outdated methods. Wet sieving was not always applied, and the lithic collections could have been biased by artefact selection and missing some by-product components. In this context, the lithic assemblage of Stajnia Cave unveils that the site maintained a similar settlement function both in the Middle and Upper Palaeolithic. In the lithic collection, diagnostic stone tools of the Szeletian, Jerzmanowician or Zwierzyniecian are absent. The morphology of one point with inverse retouch is puzzling but could not be associated with any transitional industries. Although the lithic items of the early Upper Palaeolithic are scanty and undiagnostic, the features and the direct radiocarbon dates of the pendant and the awl document human occupation during the Early Aurignacian. Thus far, evidence of bone and ivory artefacts have not been documented in any of other sites characterized by these transitional entities^19,51,52^. Hence, the previous proposals that *Homo sapiens* entered Poland only during the late phase of the Aurignacian^16,53^ should be reviewed. A reassessment of the lithic materials recovered during previous excavations at Mamutowa Cave and Kraków-Zwierzyniec is in progress. Thus far, the analysis of the Aurignacian assemblage of Kraków-Zwierzyniec trench 3 indicates technological characteristics of the Early and Recent Aurignacian. The features of the Zwierzyniec Type variant may only be an artificial construct of artefact mixture. Hitherto, the settlement of Poland during the Early Aurignacian could have been seasonal and for short periods, explaining why the evidence are so scattered and limited.

**Section 5 – ZooMS analysis**

*Virginie Sinet-Mathiot, Frido Welker, Geoff M. Smith*

The MALDI-TOF MS spectrum obtained from R-EVA 2650 (awl) was taxonomically identified as Equidae and the one from R-EVA 2651 (pendant) was determined as Elephantidae (Table S4, Fig. S5). In both cases, peptide marker series are similar between closely related species, which explain why ZooMS cannot be more taxonomically discriminant. Considering the archaeological context, R-EVA 2651 is most likely a tusk fragment from a woolly mammoth.

**Section 6 –** **NIR analysis**

*Christina M. Ryder, Matt Sponheimer*

A material’s near-infrared (NIR) spectrum reflects its chemical composition and physical structure^54^. Because key absorbance bands of amino acid functional groups appear within the near-infrared range, NIR analysis has proven useful for quantifying collagen content in archaeological bone^55,56^. Multivariate calibration algorithms predict collagen yield by modelling the sample’s spectral response to the collagen content of the calibration set. Since near-infrared light penetrates deeply into biological tissues^57,58^, NIR is an ideal spectroscopic technique to prescreen bone as the outer surface may be more vulnerable to diagenesis^59-61^. We use chemometric analyses using partial least squares regression from specimens of known collagen yield to characterize the unknown specimens (see^55^ for detailed methods) (Fig. S6).

**Section 7 – The** **use of Bayesian chronological modelling in Stajnia Cave and other Aurignacian archaeological sites with evidence of punctate objects**

*Sahra Talamo and Andrea Picin*

We base our discussion on models constructed for each site using Bayesian modelling software (OxCal 4.4)^62^ and the new IntCal20 curve^63^. For each model, a General t-type Outlier Model^62^ was computed to detect problematic samples with prior probabilities set at 5%. The ‘date’ command was used to establish the probability distribution functions (PDFs), e.g., start and end boundaries, as well as to calculate the duration of archaeological divisions. Normally, when outlier detection analysis is used to assess the robustness of the model, the generally accepted limit of 60% for the model Agreement Index is not considered relevant^62^. However, we think that if an Agreement Index is very low (less than 40%) caution should be used in interpreting the chronological boundaries of the site, and the results should be taken with a grain of salt.

Model codes are shown at the end of the SI as CQL codes designed for OxCal 4.4^62^.

Stajnia Cave chronology

The calibrated dates (un-modelled) and the modelled ages obtained for Stajnia Cave are shown in Table S5 and Fig. S7. We do not consider the model reliable due to poor agreement between the radiocarbon dates and the stratigraphy at the site. See main text for more information.

Geiβenklösterle Cave chronology

Geißenklösterle Cave is located in the Ach Valley about 3 km away from the village of Blaubeuren in Swabian Jura (Baden-Württemberg, Germany). The site was first explored in 1958 by G. Riek, and subsequently by E. Wagner in 1973, and by J. Hahn between 1974-1991. The last fieldworks were carried out by N. Conard between 2000 and 2002^64^. The stratigraphic sequence of the site is composed of 22 geological layers divided into several archaeological horizons^65^. Starting from the bottom, horizons AH VIII-IV are attributed to the Middle Palaeolithic. Above lies horizon AH III associated with the Early Aurignacian and divided into 4 sub-layers (AH IIIc, IIIb, IIIa, III), and horizon AH II ascribed to the Recent Aurignacian and divided into 5 sub-layers (AH IId, IIb, IIc, IIa, IIn). On top of the sequence is horizon AH I distributed in several sub-units related to the Gravettian, Magdalenian and Mesolithic. The site is renowned for the discovery of ivory beads in layer AH IIIa, ivory figurines in layers AH IIb-IIa (one of which characterized by a punctate motive), and a flute made of a swan radius bone in layer AH II^66-68^.

The Bayesian model constructed here follows the archaeological subdivisions and the radiocarbon ages published in^69,70^ (Table S6 and S14). The agreement index is 46.4% with two outliers detected (higher than 31%) in 25 dates.

We are aware of the discussion regarding various Bayesian model constructions between different scholars^71-73^. However, the authors of^72,73^ do not dispute the structure of the Bayesian model included here, but instead question the association of the dates to the diagnostic artefacts. The only way to resolve the issue is to directly date the diagnostic artefacts, as demonstrated in this paper.

Vogelherd Cave chronology

Vogelherd Cave is located in the Lone Valley, 4 km from the village of Niederstotzingen in Swabian Jura (Baden-Württemberg, Germany). The site was first excavated by G. Riek in 1931 and then explored by N. Conard between 2005-2012^64^. The sedimentary sequence is divided into four Middle Palaeolithic layers (IX-VI), followed by two Aurignacian layers (V-IV), two Magdalenian layers (III-II) and a Holocene deposit (I). The site is well-known for the Aurignacian figurines carved from ivory found in layers IV and V^74^.

The Bayesian model constructed here, following the archaeological subdivisions published in^74^, shows the low agreement between the 26 ^14^C dates available in^75,76^ and the poor stratigraphic integrity of the site, which result in a model agreement index of just 0.4% with 12 detected outliers (higher than 18%, Tables S7 and S14). This confirms the conclusions of Conard and Bolus^75^ concerning the mixture of samples between strata, concern over the outdated excavation methods used, and the young age of some conventional dates caused by the bulk sampling of many small bones of different ages. For these reasons, the boundaries obtained should be treated with caution and the bars included in Fig. 4b in the main text need to be considered as ‘hypothetical’ boundaries.

Abri Blanchard chronology

Abri Blanchard is located in the Vézère Valley in the commune of Sergeac, about 10 km from Les Eyzies-de-Tayac-Sireuil (Dordogne, France). The site was investigated by L. Didon in 1910-1911, M. Castanet in 1911-1912, and by D. Peyrony in 1924-1925. New fieldworks were carried out in the northern sector by R. White in 2011-2012^77^. Two levels were attributed to the Early and Recent Aurignacian (layers B and D). The site is renowned for the discoveries in these layers of a bifurcated and punctate pendant in ivory, a punctate plaque carved from antler, engraved blocks, basket-shaped bone and ivory beads, and pierced animal teeth^78,79^.

The Bayesian model is taken from^80^ but is updated using the IntCal20^63^ calibration curve and using the only two dates from Sector 4/5 reported in^78^, which belong to the Early Aurignacian (Table S8 and S14).

Abri de Castanet chronology

Abri de Castanet is located in the Vézère Valley about 25 m from the Abri de Blanchard. The site was investigated by D. Peyrony in 1911–1913 and 1924–1925. Fieldworks were then resumed by J. Pelegrin and R. White in 1995-1998, and by R. White in 2005-2012^81^. The Aurignacian level lies directly on top of the bedrock. The site is well known for the discovery of engraved blocks, basket-shaped bone and ivory beads, soft stone beads, and pierced animal teeth. The Bayesian models from North and South sectors are from^80^ (updated with IntCal20^63^) using the radiocarbon dates and the stratigraphic information published in^81^ (Tables S9 and S14).

Abri de la Souquette chronology

Abri de la Souquette is located in the Vézère Valley about 10 km from the village of Les Eyzies-de-Tayac-Sireuil (Dordogne, France). The rock-shelter is situated on the western slope on the opposite side to the Abri Castanet and Abri Blanchard. The site was first explored in 1902-1903 by abbé M. A. Landesque, ransacked by Costes and Letellier in 1903-1910, and in 1910 sub-leased to Otto Hauser who carried out excavations in order to selling the flint artefacts. In 1980-1982, a small area at the southern extremity of the rock-shelter was investigated by A. Roussot^82^. The stratigraphy is composed of 11 archaeological horizons, and layer 11, lying directly atop the bedrock, is attributed to Aurignacian^82^. The site is well known for abundant ivory beads and pendants, talc, and the three carved “shell” facsimiles with punctate decoration on the distal surface^82^. We built a Bayesian model to determine the duration of the Aurignacian phase (layer 11) using the radiocarbon dates and the stratigraphic information published in^83^. A little remark regarding the OxA code in Table 3 of^82^: there should be a typo error since the OxA code is the same (OxA-32198) but belongs to two different samples numbers (SQT11,Z23 201 and one to SQT11,Z23 68). We maintain the same OxA code in our model. The chronological resolution of the sequence is limited to four radiocarbon dates in good agreement between each other (Tables S10 and S14).

Tuto de Camalhot Cave chronology

Tuto de Camalhot cave is located at Saint-Jean-de-Verges in the department of Ariège in southwestern France. The site was excavated in 1927-1934 by Vezian^84^ revealing a short stratigraphic sequence composed of two Aurignacian layers (70-80, and 50) overlaid by a Noaillian Gravettian layer. The two Aurignacian layers are clearly divided inside the cave but they became undistinguishable on the terrace in front of the natural shelter^84^. Techno-typological analysis of the lithic assemblages indicates that the lower layer 70-80 belongs to the Early Aurignacian whereas the presence of carinated and *busqué* burins places the upper layer 50 in the Recent Aurignacian^5,84^. In the collection, split-based bone points and lissoirs, decorated objects and personal ornaments are found. Pendants and pierced shells are numerous and mostly associated with the upper layer 50^84^. Within this group, it worth noting an ivory pendant with striation and a regular punctate pattern^84^. The precise location of this object is not documented but following Vezian^84^ (page 118) it should belong to the Recent Aurignacian.

We built a Bayesian model to determine the duration of the Aurignacian phase of the site using the radiocarbon dates and the stratigraphic information published in^5,85^.

Although the model agreement index of the Bayesian Model is higher than 60% (Tables S11 and S14), it should be noted that the chronology is based only on two bone samples, sampled from a bag without any stratigraphic information and stored at the Museum of Ariège^5^. Thus, the boundaries obtained from the Bayesian model should be considered ‘hypothetical’ until further chronological work is carried out to contextualize the Aurignacian settlement at the site in more detail.

Sungir

The open-air site of Sungir is located near the town of Vladimir (Russia) on the left bank of the ancient Klyazma River, near its join with the Sungir stream. The site was excavated by N. Bader in several field campaigns between 1957 – 1977, and 1987 – 1995^86^. The area excavated exceed 4,500 m^2^ and comprises four main dwelling structures and abundant archaeological materials. In 1964, a single burial of an adult male (Sungir 1) and an isolated skull (Sungir 5) on an ochre spot were found. In 1969, a second burial containing two juveniles, a male (Sungir 2) and probably a female (Sungir 3), and an isolated human femur (Sungir 4) were discovered^86^. The three burials were covered in red ochre, and the corpses magnificently decorated with ivory beads, pendants and mobiliary arts^87^.

The Bayesian model constructed here, to determine the ages the burials, follows the archaeological subdivisions and the radiocarbon ages published in^88^ (Tables S12 and S14).

Yana

The open-air site complex of Yana is located on the left bank of the Yana River in the Siberian Arctic. It was discovered in 1993 and fieldworks were carried out between 2001 and 2008^89^. The site is composed of six localities distributed few hundred meter apart^89^. In three localities (Upstream Point, ASN and SP), only surface finds were discovered whereas in the others (Yana B, Yana RHS/NP and TUMS 1) in situ cultural layers were detected. These cultural horizons are thought to be roughly contemporaneous and part of the same settlement during recurrent forays along the Yana River^89,90^. Due to erosion processes, the summer water level of the river is used a baseline for determining the measurement of the stratigraphic succession^91^. The lower terrace, containing the archaeological occupation is frozen, and rises to 16-18 m above water level (a.w.l.). The bottom layer is composed of bedded sandy loam, and at 12 m a.w.l. silt replaces loam. The terrace deposit includes syngenetic ice wedges up to 2 m wide. The cultural horizon is located at about 7.5 m a.w.l.^91^. The site Yana B is located near a large accumulation of mammoth skeletal remains (site YMAM) and is characterized by lithic micro-projectiles, bone and antler tools for working ivory^92^. The site Yana RSH/NP (Northern Point) is situated at small distance on the east. The fieldworks unearthed ivory artefacts, preforms, semi-ﬁnished products, and by-products, together with micro stone tools, backed artefacts and especially quartz-crystal implements^92^. The site TUMS 1 is located at few hundred meters east from Yana RSH/NP and includes lithic items and faunal bones^91^. In these three locations, beyond numerous processed carcasses of cold adapted species, more than 300 personal ornaments and decorated object were found^89,91^.

We built a Bayesian model to determine the duration of the cultural layers in RSH/NP, RHS /B and RHS/TUMS 1 using the radiocarbon dates and the stratigraphic information published in^89-92^ (Tables S13 and S14).

**Section 8 – Experimental data on ivory tusk**

*Marcin Diakowski and Marcin Binkowski*

To investigate manufacturing technology of the decorated pendant from Stajnia Cave, a series of experiments has been undertaken (Table S15). The several variants were considered, referring to both raw material and technology, which could have influenced a mode of artefact production. In the experiments, both fresh and boiled bones of domestic cattle were used, because the cementum, from which the plate was made, has similar working properties to cortical bone^93,94^. Taking into consideration the possibility of secondary material use, a cementum from a mammoth tusk deposited in cave environment for 15,000 years was worked as well (Figs. S8-S10). The mammoth tusk used to experiment was provided by the Department of Paleozoology Wrocław University. To obtain similar technological traces of shaping and making dots observed on the plate, in the course of the experiments, a variety of tools, such as flakes, blades, burins and perforators were used. Moreover, several working techniques were applied: scraping (plate shaping) and indirect percussion, single and double directional boring (decoration). Particular dots were made with various intensity. Each experiment has been performed three times what resulted in making 500 dots.

In the course of the experiments, it turned out the working prehistoric material did not result in making similar traces as the ones observed on the plate from the Stajnia Cave. Due to the state of preservation, such as numerous cracks, exfoliation, fossilization of dorsal surface, cementum etc., working was completely ineffective as compared to fresh bones (see for comparison Figs. S11-S12 and Figs. S13-S15). In the course of scraping the ventral surface of cementum, some traces typical for this technique were observed, however large pieces of surface fell off in an uncontrolled way (Figs. S11-S12). While making a decoration, a tool tip hardly engages in the tooth surface, and the tooth structure was crushed and cracked in the hole area.


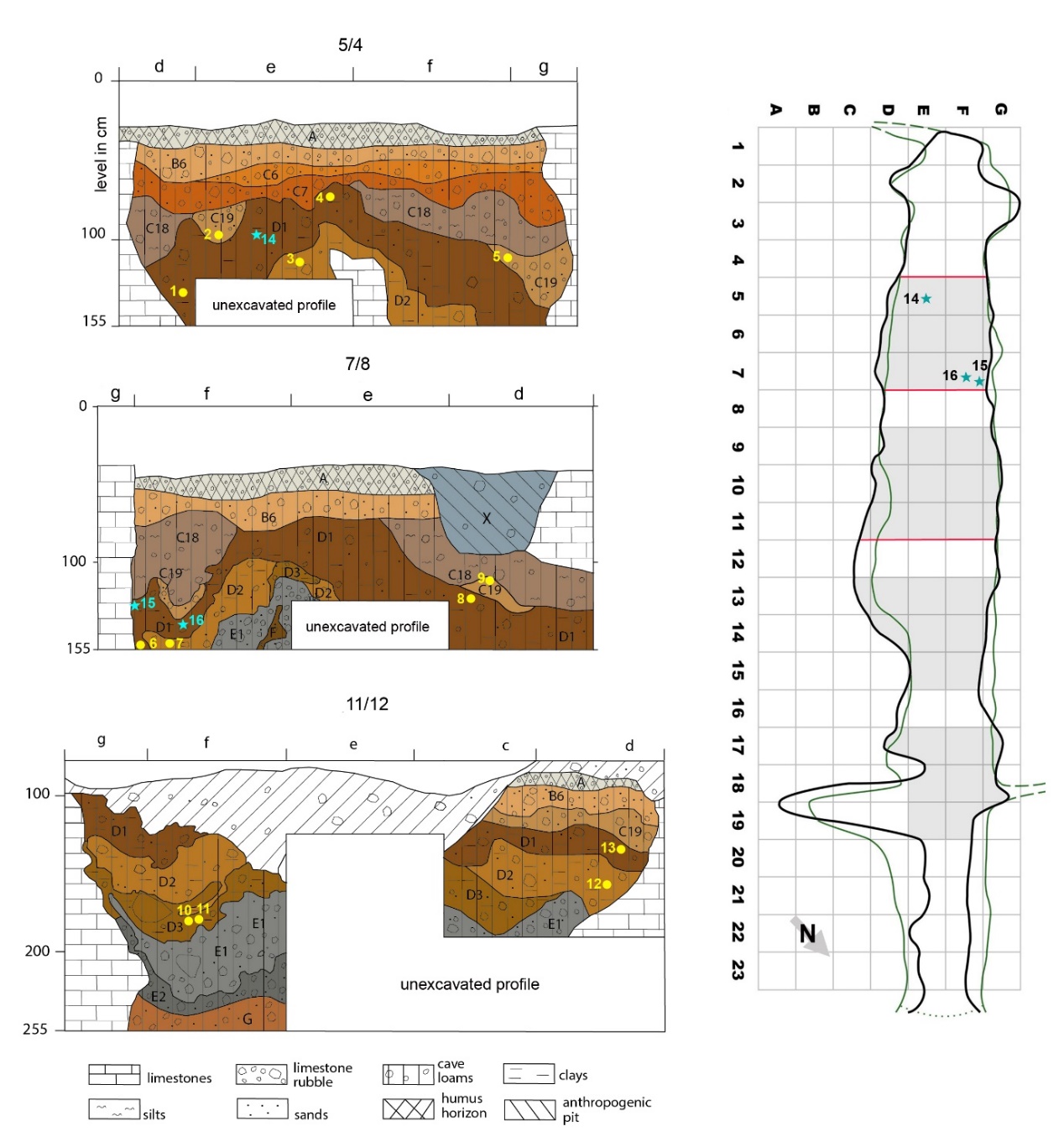


Fig. S1. Planimetry of Stajnia Cave and stratigraphy with location of the 14C AMS samples (1 - S-24390; 2 - S-13694; 3 - S-16187; 4 - S-17162; 5 - S-11613; 6 - S-24106; 7 - S-23855; 8 - S-9547; 9 - S-11340; 10 - S-11572; 11 - S-12722; 12 - S-12305; 13 - S-12182), awl (14 - S-12160), and pendant (15 - S-22222; 16 - S-23100). The figure was generated using Adobe Photoshop CS5 12.0 software.


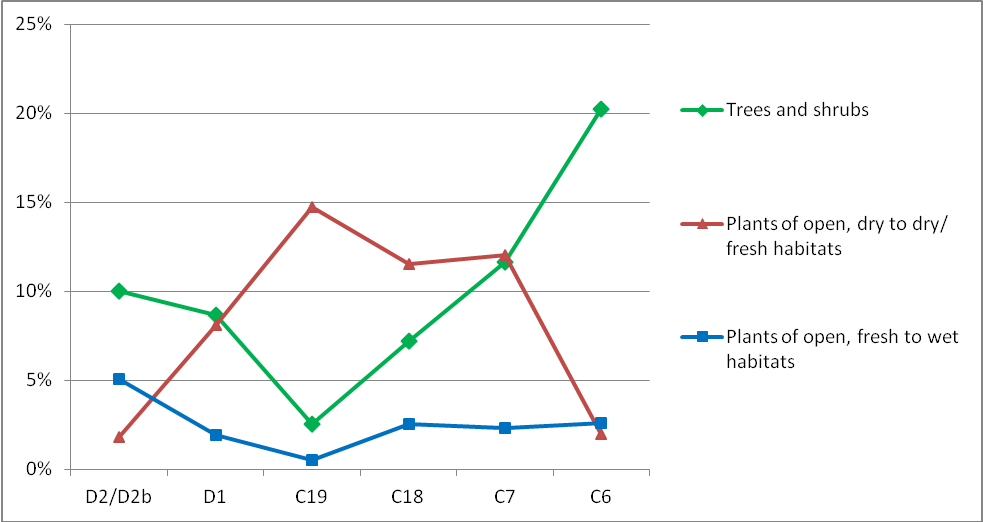


Fig. S2. The shift in the relative frequencies of plants representing different demands in terms of precipitation, juxtaposed with the relative frequencies of trees and shrubs according to the stratigraphic sequence (from D2/D2b to C6). The tendencies throughout the stratigraphic sequence of Stajnia Cave are consistent with the geo-chronological data observed.


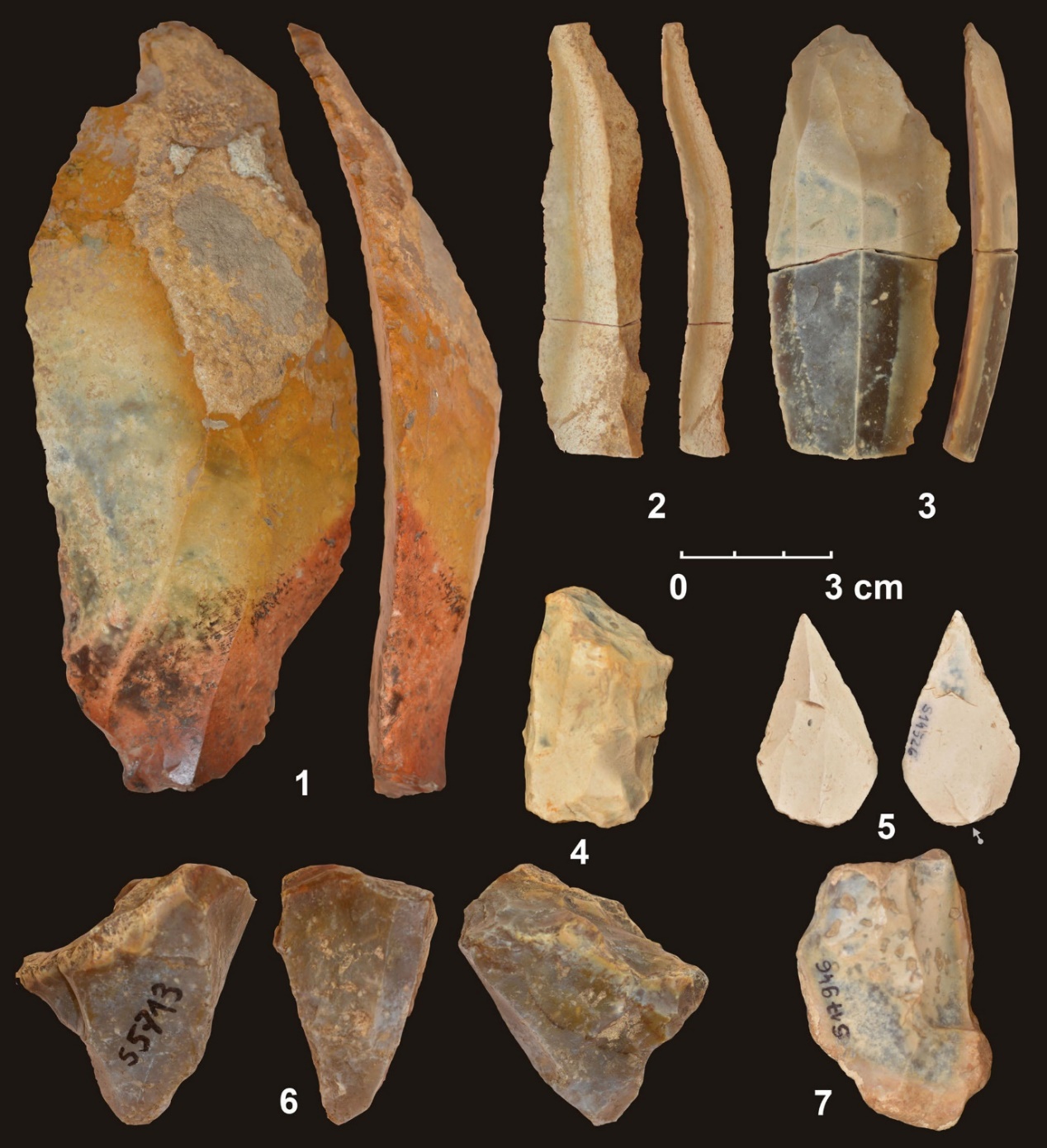


Fig. S3. Laminar artefacts and laminar cores from Stajnia Cave: 1) semi-cortical blade – Dump, 2) semi-cortical blade – layer D1, 3) Aurignacian blade – layer C, 4) bladelet core – layer E2, 5) retouched point – layer D1, 6) bladelet core – Dump, 7) core-on-flake – layer D1.


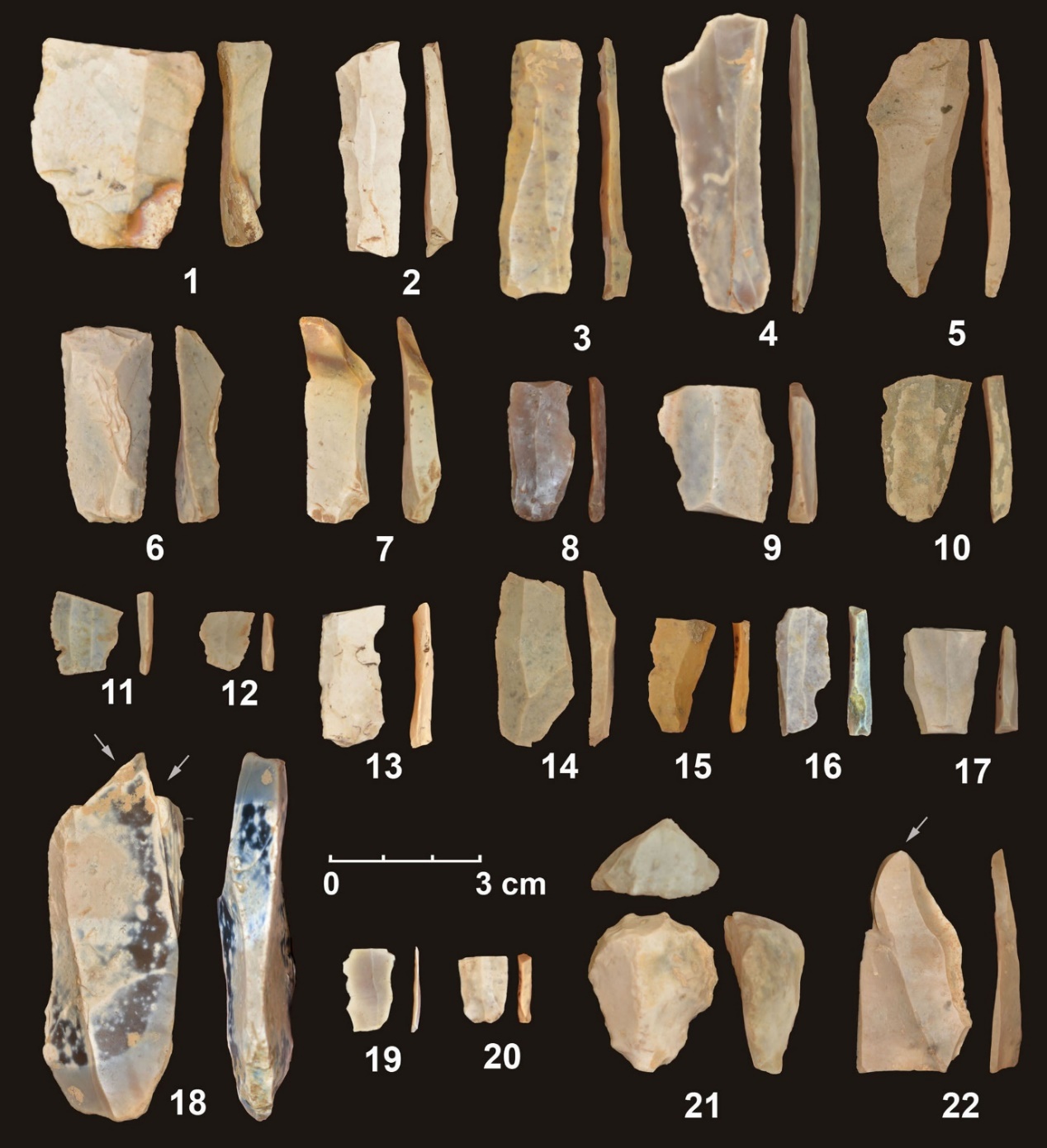


**Fig. S4.** Laminar by-products and retouched tools: 1) blade fragment – Dump, 2) blade fragment – Dump, 3) blade fragment – Dump, 4) blade – Dump, 5) blade – layer C18, 6) crested blade – layer D1, 7) blade – layer D1, 8) bladelet fragment – Dump, 9) blade fragment – layer D1, 10) blade fragment – layer C , 11) bladelet fragment – layer C, 12) bladelet fragment – layer C, 13) bladelet – Dump, 14) blade fragment – layer D1, 15) bladelet fragment – layer C, 16) bladelet fragment – Dump, 17) blade fragment – layer D1, 18) burin – layer D2; 19) bladelet fragment – Dump, 20) bladelet fragment – Dump, 21) endscraper – layer D2; 22) burin (?) – layer D1.


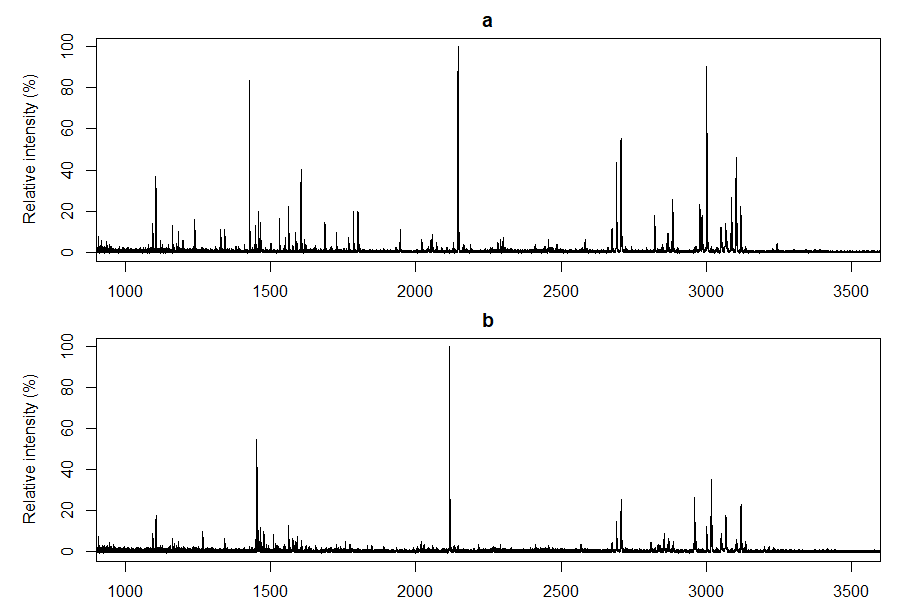


Fig. S5. MALDI-TOF MS spectra for R-EVA 2650 (a) and R-EVA 2651 (b).


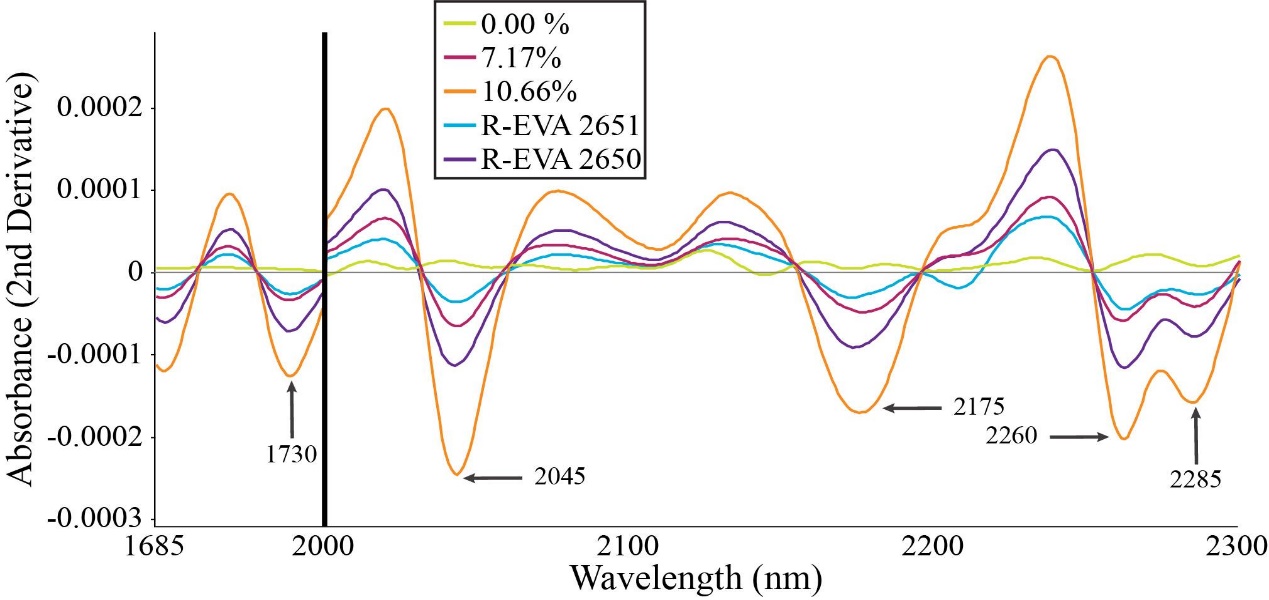


Fig. S6. NIR absorbance spectra (Savitzky-Golay second derivative; derivative order = 2; polynomial order = 3; 31 points smoothing) of R-EVA 2651 (blue), R-EVA 2650 (purple), and three archaeological specimens with collagen yields of 0.00% (green), 7.17% (magenta), and 10.66% (orange). Labelled bands show directional shifts expected with increasing collagen yield.

**
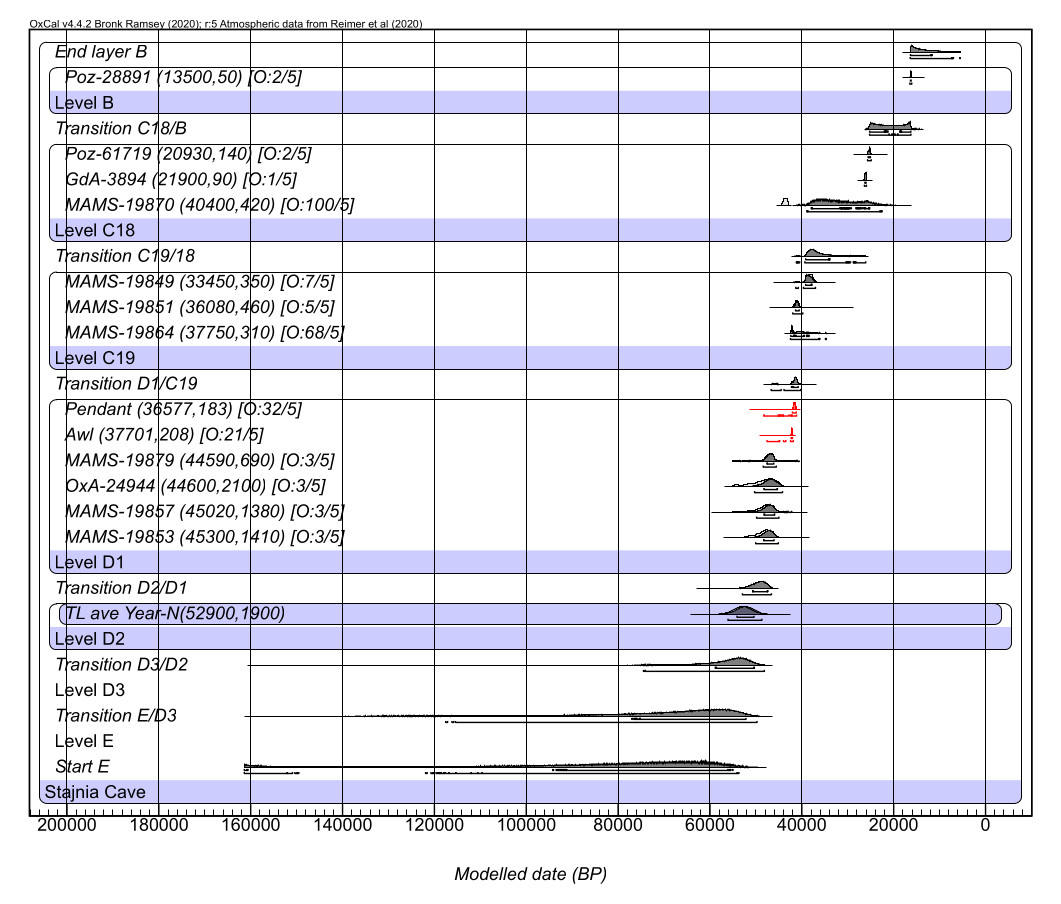
**

**Fig. S7**. Bayesian Model of Stajnia cave. Radiocarbon dates were calibrated and modelled using IntCal20^63^ in the OxCal 4.4 program^62^. Outliers prior and posterior probabilities are shown in square brackets. All the samples >49,000 BP are not included in the iterations. The pendant and the awl are in red.

**
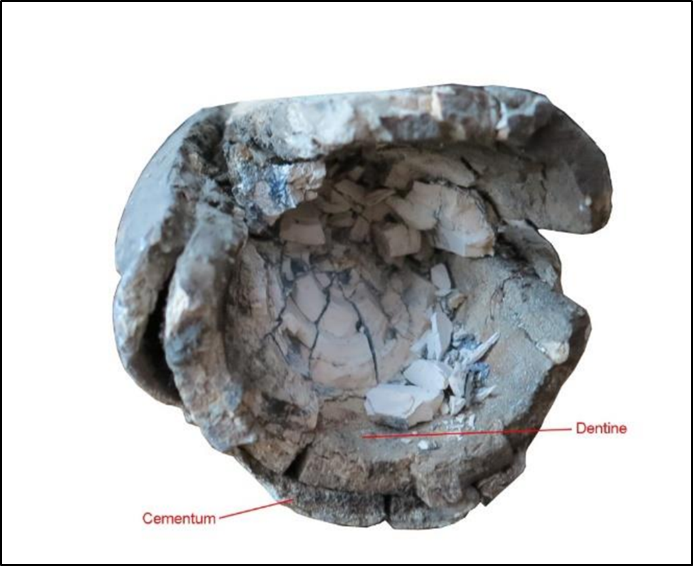
**

**Fig. S8:** The mammoth tusk used to experiment – the structural parts are described.


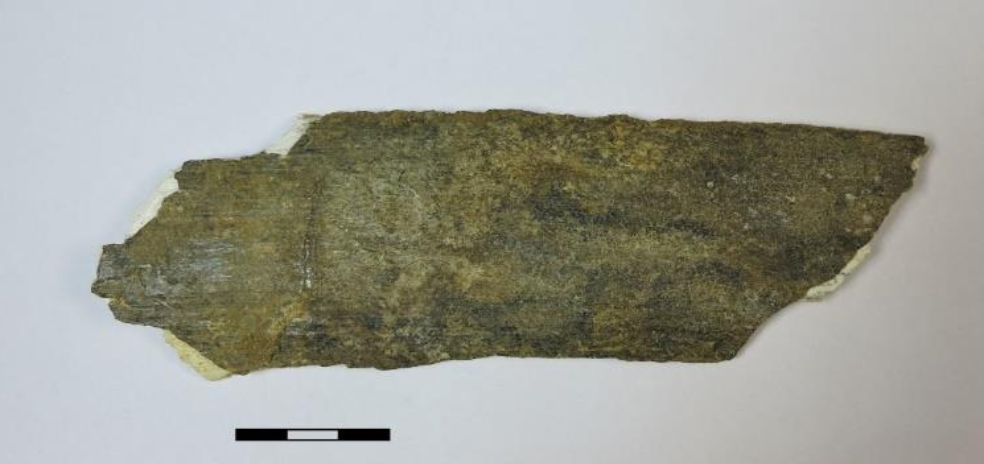


**Fig. S9:** Dorsal side of the cementum (mammoth tusk).


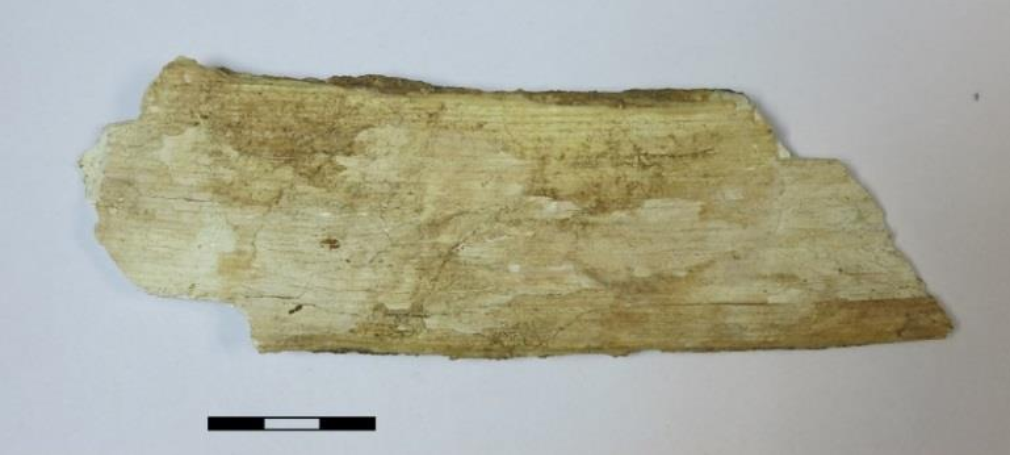


**Fig. S10**: Ventral side of the cementum (mammoth tusk)


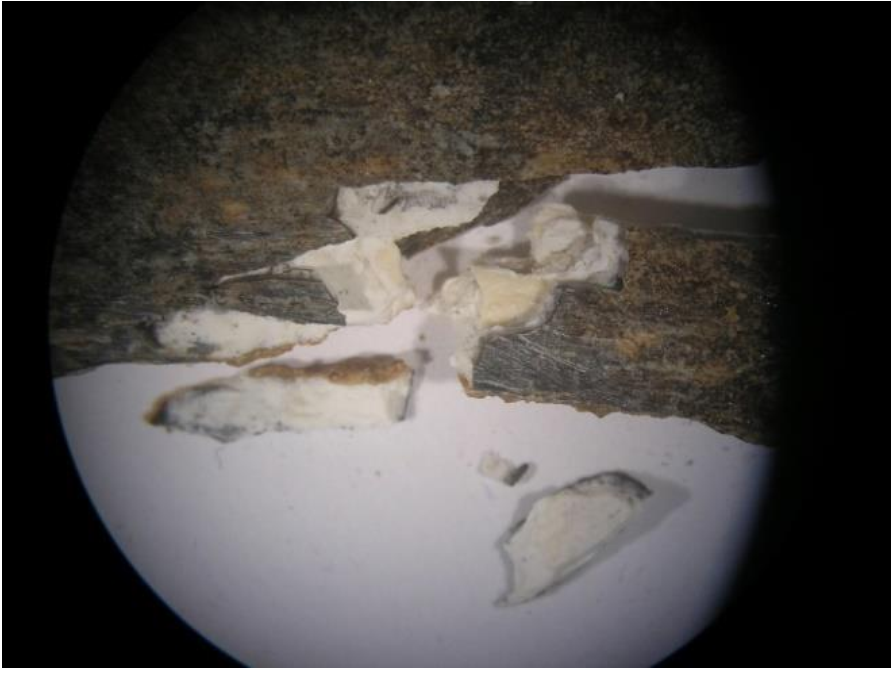


**Fig. S11:** Tusk structure was crushed and cracked in hole area. Under pressure, in some cases cementum fragments of the mammoth tusk broke down into smaller pieces.


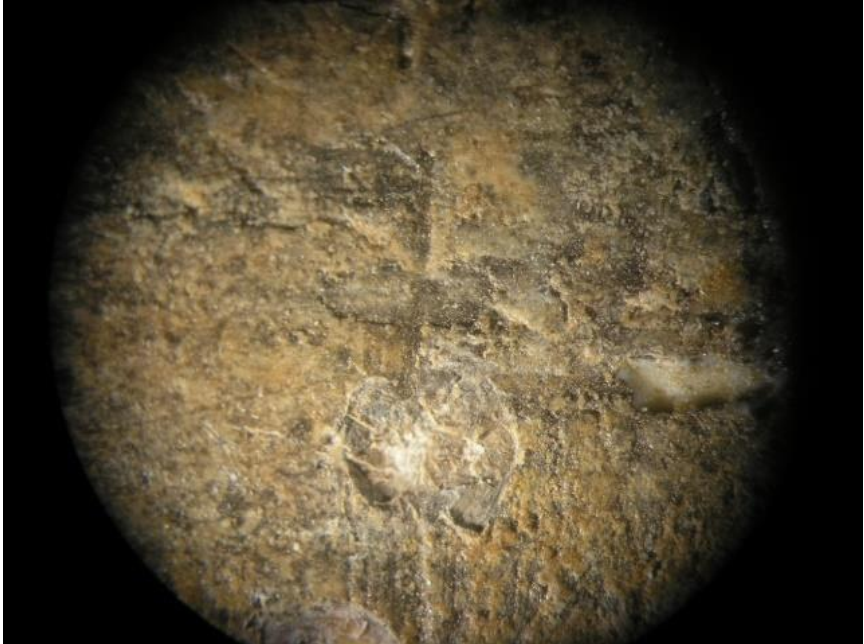


**Fig. S12:** Large pieces of surface fell off in uncontrolled way – the cementum fragments of the mammoth tusk.


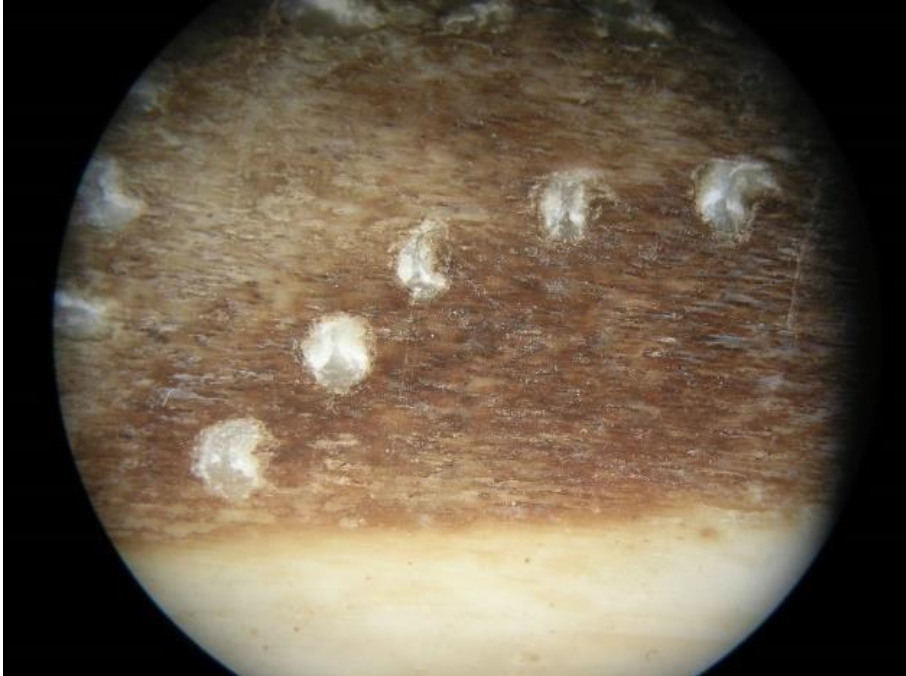


**Fig. S13:** Boring dots on the cattle humerus with use of the burin tip.


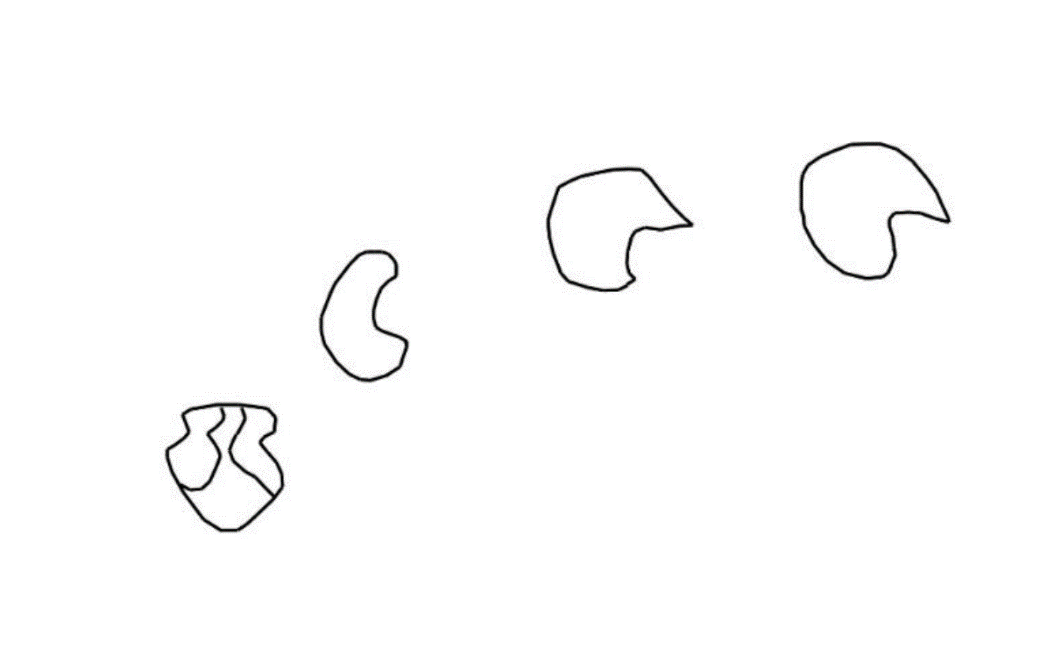


**Fig. S14:** Boring dots on cattle rib with use of the burin tip.

**
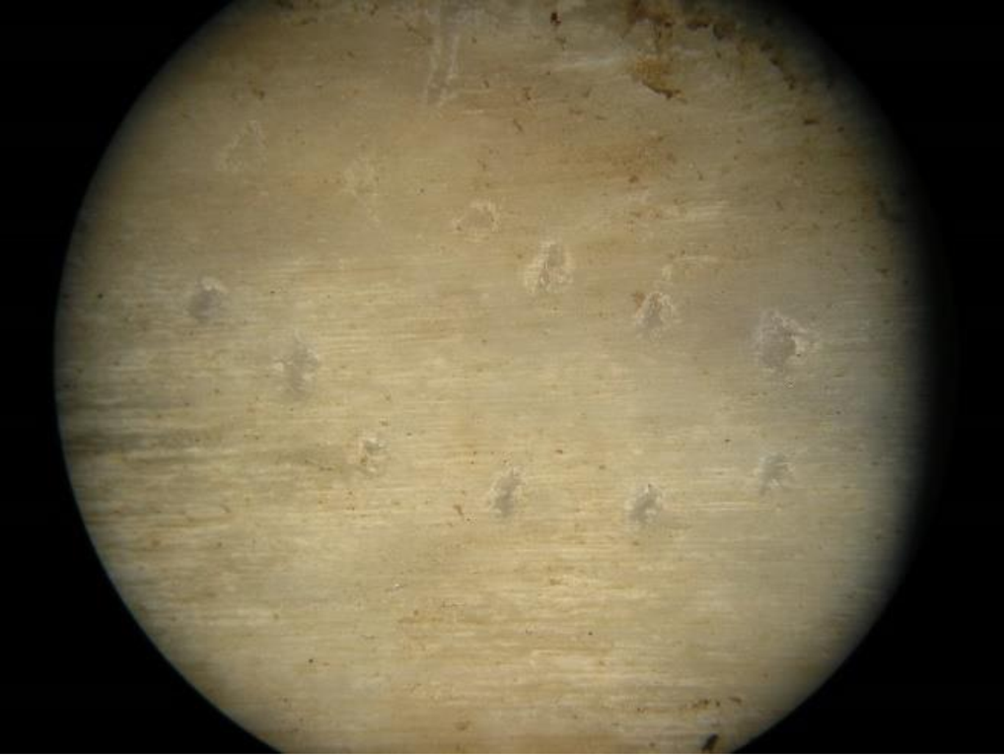
**

**Fig. S15:** Dots made by indirect percussion on the cattle humerus with use of the borer tip.

|  | **Units** | | | | | | | |
| --- | --- | --- | --- | --- | --- | --- | --- | --- |
| ***Type of plant*** | **D2** | **D2b/D** | **D2b** | **D1** | **C19** | **C18** | **C7** | **C6** |
| **Trees and shrubs AP** | 11 | 10 | 10 | 9 | 3 | 7 | 12 | 20 |
| **Dwarf shrubs** | 1 |  |  |  | 1 | 1 | 1 |  |
| **Herb plants** | 78 | 75 | 74 | 85 | 91 | 79 | 68 | 58 |
| **Bryophyta** |  |  | 1 |  |  | 1 |  |  |
| **Pteridophyta** | 1 |  |  |  | 1 |  |  | 4 |
| **Other** | 10 | 14 | 15 | 6 | 5 | 12 | 19 | 18 |
| ***Type of environment*** | **D2** | **D2b/D** | **D2b** | **D1** | **C19** | **C18** | **C7** | **C6** |
| **Eurytopic** | 68 | 68 | 68 | 75 | 76 | 65 | 54 | 54 |
| **Plants of open, dry to intermediate habitats** | 3 | 2 | 2 | 8 | 15 | 12 | 12 | 2 |
| **Plants of open, intermediate to wet habitats** | 7 | 5 | 4 | 2 | 1 | 3 | 2 | 3 |
| **All the rest** | 22 | 25 | 26 | 15 | 9 | 21 | 32 | 42 |

**Table S1**. Percentage proportions of pollen and spores related to the environment type in stratigraphic sequence.

|  | **Dump** | | **A** | | **B** | | **C4+C6** | | **C7+C9** | | **C18** | | **C19** | | **D1** | | **D2** | | **D3** | | **E1** | | **E2** | | **Total** | |
| --- | --- | --- | --- | --- | --- | --- | --- | --- | --- | --- | --- | --- | --- | --- | --- | --- | --- | --- | --- | --- | --- | --- | --- | --- | --- | --- |
|  | **N** | ***%*** | **N** | **%** | **N** | **%** | **N** | **%** | **N** | ***%*** | **N** | ***%*** | **N** | **%** | **N** | ***%*** | **N** | ***%*** | **N** | ***%*** | **N** | **%** | **N** | **%** | **N** | ***%*** |
| **Cortical flake** | 1 | *1* |  |  |  |  |  |  | 2 | *5.9* | 1 | *3.1* |  |  | 9 | *3.8* | 3 | *3.8* |  |  |  |  |  |  | 16 | *2.9* |
| **Cortical blade** | 1 | *1* | 1 | *6.7* |  |  |  |  |  |  |  |  |  |  |  |  |  |  |  |  |  |  |  |  | 2 | *0.4* |
| **Ordinary flake** | 14 | *13.6* | 2 | *13.3* |  |  | 1 | *16.7* | 5 | *14.7* | 5 | *15.6* |  |  | 25 | *10.6* | 10 | *12.5* | 2 | *20* |  |  | 1 | *20* | 65 | *11.9* |
| **Ordinary blade** | 4 | *3.9* |  |  |  |  | 1 | *16.7* |  |  | 3 | *9.4* | 1 | *50* | 5 | *2.1* |  |  |  |  |  |  |  |  | 14 | *2.6* |
| **Bladelet** | 2 | *1.9* |  |  |  |  |  |  | 2 | *5.9* |  |  |  |  |  |  |  |  |  |  |  |  |  |  | 4 | *0.7* |
| **Predetermining Lev. flake** |  |  |  |  |  |  |  |  |  |  | 1 | *3.1* |  |  | 2 | *0.8* |  |  |  |  |  |  |  |  | 3 | *0.6* |
| **Levallois rec. unidir. flake** |  |  |  |  |  |  |  |  |  |  |  |  |  |  | 3 | *1.3* |  |  |  |  |  |  |  |  | 3 | *0.6* |
| **Levallois rec. bidir. flake** | 1 | *1* |  |  |  |  |  |  |  |  |  |  |  |  | 2 | *0.8* |  |  |  |  |  |  |  |  | 3 | *0.6* |
| **Levallois rec. centr. flake** |  |  |  |  |  |  |  |  |  |  | 1 | *3.1* |  |  |  |  | 1 | *1.3* |  |  |  |  |  |  | 2 | *0.4* |
| **Levallois point** |  |  |  |  |  |  |  |  |  |  | 1 | *3.1* |  |  | 1 | *0.4* |  |  |  |  |  |  |  |  | 2 | *0.4* |
| **Levallois flake undeter.** |  |  |  |  |  |  |  |  |  |  |  |  |  |  | 2 | *0.8* | 2 | *2.5* |  |  |  |  |  |  | 4 | *0.7* |
| **Core-edge removal flake** | 3 | *2.9* |  |  |  |  |  |  |  |  | 2 | *6.3* |  |  | 5 | *2.1* | 3 | *3.8* |  |  |  |  | 1 | *20* | 14 | *2.6* |
| **Pseudo-Levallois point** |  |  |  |  |  |  |  |  |  |  |  |  |  |  | 1 | *0.4* |  |  |  |  |  |  |  |  | 1 | *0.2* |
| **Unidirectional flake** | 1 | *1* |  |  |  |  |  |  | 1 | *2.9* | 1 | *3.1* |  |  | 10 | *4.2* | 4 | *5.0* | 1 | *10* |  |  |  |  | 18 | *3.3* |
| **Centripetal flake** | 5 | *4.9* |  |  |  |  |  |  | 1 | *2.9* | 1 | *3.1* |  |  | 13 | *5.5* | 3 | *3.8* |  |  |  |  |  |  | 23 | *4.2* |
| **Orthogonal flake** |  |  |  |  |  |  |  |  |  |  |  |  |  |  | 1 | *0.4* |  |  |  |  |  |  |  |  | 1 | *0.2* |
| **Crested blade** |  |  |  |  | 1 | *25* |  |  |  |  |  |  |  |  | 1 | *0.4* |  |  |  |  |  |  |  |  | 2 | *0.4* |
| **Tablette** | 2 | *1.9* |  |  |  |  |  |  |  |  | 1 | *3.1* |  |  |  |  |  |  |  |  |  |  |  |  | 3 | *0.6* |
| **Reshaping flaking surface** | 1 | *1* |  |  |  |  |  |  | 1 | *2.9* |  |  |  |  | 1 | *0.4* | 3 | *3.8* |  |  |  |  |  |  | 6 | *1.1* |
| **Kombewa-type flake** |  |  |  |  |  |  |  |  |  |  |  |  |  |  | 2 | *0.8* |  |  |  |  |  |  |  |  | 2 | *0.4* |
| **Bifacial shaping flake** | 1 | *1* |  |  |  |  |  |  |  |  | 1 | *3.1* |  |  | 1 | *0.4* | 1 | *1.3* |  |  |  |  |  |  | 4 | *0.7* |
| **Knapping accident** | 1 | *1* |  |  |  |  |  |  | 1 | *2.9* |  |  |  |  | 1 | *0.4* | 1 | *1.3* |  |  |  |  |  |  | 4 | *0.7* |
| **Flake frag.** | 25 | *24.3* | 6 | *40* |  |  |  |  | 7 | *20.6* | 3 | *9.4* |  |  | 59 | *25.0* | 21 | *26.3* | 5 | *50* | 8 | *53.3* | 2 | *40* | 136 | *25.0* |
| **Blade frag.** | 9 | *8.7* | 1 | *6.7* | 2 | *50* |  |  | 2 | *5.9* | 1 | *3.1* |  |  | 4 | *1.7* |  |  |  |  | 2 | *13.3* |  |  | 21 | *3.9* |
| **Bladelet frag.** | 3 | *2.9* |  |  |  |  | 2 | *33.3* | 2 | *5.9* | 1 | *3.1* |  |  |  |  |  |  |  |  |  |  |  |  | 8 | *1.5* |
| **Debris** | 6 | *5.8* |  |  | 1 | *25* |  |  | 1 | *2.9* | 2 | *6.3* |  |  | 7 | *3.0* | 4 | *5.0* |  |  |  |  |  |  | 21 | *3.9* |
| **Preform of bifacial tool** | 1 | *1* |  |  |  |  |  |  |  |  | 1 | *3.1* |  |  | 5 | *2.1* |  |  |  |  | 1 | *6.7* |  |  | 8 | *1.5* |
| **Bifacial tool** |  |  |  |  |  |  |  |  |  |  |  |  |  |  | 4 | *1.7* | 1 | *1.3* |  |  |  |  |  |  | 5 | *0.9* |
| **Bifacial tool frag.** |  |  |  |  |  |  |  |  | 1 | *2.9* |  |  |  |  | 3 | *1.3* | 1 | *1.3* |  |  |  |  |  |  | 5 | *0.9* |
| **Leaf point frag.** |  |  |  |  |  |  |  |  |  |  |  |  |  |  | 1 | *0.4* |  |  |  |  |  |  |  |  | 1 | *0.2* |
| **Scrapers** |  |  | 1 | *6.7* |  |  |  |  | 1 | *2.9* | 1 | *3.1* |  |  | 12 | *5.1* | 3 | *3.8* |  |  |  |  | 1 | *20* | 19 | *3.5* |
| **Groszak** | 1 | *1* |  |  |  |  |  |  |  |  |  |  |  |  | 1 | *0.4* |  |  |  |  |  |  |  |  | 2 | *0.4* |
| **Endscraper** |  |  |  |  |  |  | 1 | *16.7* | 1 | *2.9* |  |  |  |  | 1 | *0.4* | 1 | *1.3* |  |  |  |  |  |  | 4 | *0.7* |
| **Burin** |  |  |  |  |  |  |  |  |  |  |  |  |  |  | 1 | *0.4* | 1 | *1.3* |  |  |  |  |  |  | 2 | *0.4* |
| **Notched tool** |  |  |  |  |  |  |  |  |  |  |  |  |  |  | 3 | *1.3* |  |  |  |  |  |  |  |  | 3 | *0.6* |
| **Point** |  |  |  |  |  |  |  |  | 1 | *2.9* |  |  |  |  | 2 | *0.8* |  |  |  |  |  |  |  |  | 3 | *0.6* |
| **Retouched tool frag.** | 4 | *3.9* | 1 | *6.7* |  |  | 1 | *16.7* |  |  | 2 | *6.3* |  |  | 9 | *3.8* | 2 | *2.5* |  |  | 1 | *6.7* |  |  | 20 | *3.7* |
| **Core** | 16 | *15.5* | 2 | *13.3* |  |  |  |  | 4 | *11.8* | 2 | *6.3* | 1 | *50* | 34 | *14.4* | 15 | *18.8* | 2 | *20* | 5 | *33.3* | 1 |  | 82 | *15.0* |
| **Core frag.** | 1 | *1* | 1 | *6.7* |  |  |  |  | 1 | *2.9* | 1 | *3.1* |  |  | 5 | *2.1* |  |  |  |  |  |  |  |  | 9 | *1.7* |
| **Total** | 103 | *100* | 15 | *100* | 4 |  | 6 | *100* | 34 | *100* | 32 | *100* | 2 | *100* | 236 | *100* | 80 | *100* | 10 | *10* | 17 | *113* | 6 | *100* | 545 | *100* |

**Table S2.** Number and percentage of the lithic assemblages (>2cm) of Stajnia Cave.

|  | **Dump** | | **A** | | **C7+C9** | | **C18** | | **C19** | | **D1** | | **D2** | | **E1** | | **E2** | |
| --- | --- | --- | --- | --- | --- | --- | --- | --- | --- | --- | --- | --- | --- | --- | --- | --- | --- | --- |
|  | **N** | ***%*** | **N** | ***%*** | **N** | ***%*** | **N** | ***%*** | **N** | ***%*** | **N** | ***%*** | **N** | **%** | **N** | **%** | **N** | **%** |
| **Levallois** |  |  |  |  |  |  |  |  |  |  | 1 | *2.6* | 1 | *6.7* |  |  |  |  |
| **Discoid** | 2 | *11.8* |  |  | 1 | *20* | 1 | *33.3* | 1 | *50* | 8 | *20.5* | 4 | *26.7* | 2 | *40* |  |  |
| **Hierarchized** | 6 | *35.3* |  |  | 3 | *60* | 1 | *33.3* |  |  | 11 | *28.2* | 2 | *13.3* |  |  |  |  |
| **Unidirectional** |  |  | 1 | *33.3* |  |  |  |  |  |  | 8 | *20.5* | 4 | *26.7* | 2 | *40* |  |  |
| **Orthogonal** | 1 | *5.9* |  |  |  |  |  |  |  |  |  |  | 1 | *6.7* |  |  |  |  |
| **Centripetal** |  |  |  |  |  |  |  |  |  |  |  |  | 1 | *6.7* | 1 | *20* |  |  |
| **Polyhedral** | 3 | *17.6* |  |  |  |  |  |  |  |  | 1 | *2.6* | 1 | *6.7* |  |  |  |  |
| **Laminar** | 1 | *5.9* |  |  |  |  |  |  |  |  |  |  |  |  |  |  |  |  |
| **Lamellar** | 1 | *5.9* |  |  |  |  |  |  |  |  | 1 | *2.6* |  |  |  |  | 1 | *100* |
| **Tested blocks** | 1 | *5.9* | 1 | *33.3* |  |  |  |  |  |  |  |  |  |  |  |  |  |  |
| **Core-on-flake** | 1 | *5.9* |  |  |  |  |  |  |  |  | 4 | *10.3* | 1 | *6.7* |  |  |  |  |
| **Core fragment** | 1 | *5.9* | 1 | *33.3* | 1 | *20* | 1 | *33.3* | 1 | *50* | 5 | *12.8* |  |  |  |  |  |  |
| **Total** | 17 | *100* | 3 | *100* | 5 | *100* | 3 | *100* | 1 | *100* | 39 | *100* | 15 | *100* | 5 | *100* | 1 | *100* |

**Table S3.** Number and percentage of the core assemblages of the archaeological layers of Stajnia Cave.

| **Sample** | **Barcode ID** | **A** | **B** | **C** | **D** | **E** | **F** | **G** |
| --- | --- | --- | --- | --- | --- | --- | --- | --- |
| **Peptide position*** |  | COL1ɑ2  978–990 | COL1ɑ2  484–498 | COL1ɑ2  502–519 | COL1ɑ2  793–816 | COL1ɑ1  586–618 | COL1ɑ1  508–519 | COL1ɑ2 757–789 |
| R-EVA 2650 | Equidae | 1182+1198 | 1427 | 1550 | 2145 | 2820 | 2883 | 2999 |
| R-EVA 2651 | Elephantidae | absent | 1453 | absent | 2115 | 2808 | 2853+2869 | 2999+3015 |

**Table S4.** Collagen peptide marker masses observed for samples R-EVA 2650 and R-EVA 2651**.** *Peptide position given in reference to the first position of the first G-X-Y triplet of the COL1α1 and COL1α2 protein sequences.

| **Stajnia Cave** | **Un-modelled (cal BP)** | | | | **Modelled (cal BP)** | | | | **Posterior outlier probability** |
| --- | --- | --- | --- | --- | --- | --- | --- | --- | --- |
| Indices Amodel 23.9 Aoverall 34.5 | **from** | **to** | **from** | **to** | **from** | **to** | **from** | **to** |  |
|  | **68.30%** | | **95.40%** | | **68.30%** | | **95.40%** | |  |
| End layer B |  |  |  |  | 16360 | 10680 | 23230 | 5520 |  |
| Poz-28891 (13500;50) | 16360 | 16180 | 16470 | 16080 | 16390 | 16160 | 25220 | 15290 | 14% |
| Level B |  |  |  |  |  |  |  |  |  |
| Transition C18/B |  |  |  |  | 25340 | 16260 | 25380 | 16260 |  |
| Poz-61719 (20930;140) | 25550 | 25050 | 25680 | 24910 | 25560 | 25060 | 25690 | 24900 | 1% |
| GdA-3894 (21900;90) | 26280 | 26000 | 26370 | 25930 | 26290 | 26000 | 26380 | 25910 | 1% |
| MAMS-19870 (40400;420) | 43950 | 43100 | 44310 | 42900 | 38460 | 25720 | 39080 | 24430 | 100% |
| Level C18 |  |  |  |  |  |  |  |  |  |
| Transition C19/18 |  |  |  |  | 39210 | 34330 | 41450 | 26100 |  |
| MAMS-19849 (33450;350) | 38930 | 37680 | 39290 | 37180 | 39190 | 37840 | 41560 | 37020 | 9% |
| MAMS-19851 (36080;460) | 41550 | 40740 | 41970 | 40370 | 41370 | 40580 | 41920 | 39930 | 4% |
| MAMS-19864 (37750;310) | 42320 | 42060 | 42440 | 41900 | 42390 | 38760 | 42510 | 34180 | 69% |
| Level C19 |  |  |  |  |  |  |  |  |  |
| Transition D1/C19 |  |  |  |  | 42250 | 40820 | 46920 | 40180 |  |
| R_Combined Pendant (36577;183) | 41730 | 41340 | 41900 | 41210 | 41990 | 41250 | 48060 | 41150 | 30% |
| R_Combined Awl (37701;208) | 42270 | 42070 | 42360 | 41960 | 42320 | 42030 | 47780 | 41830 | 19% |
| MAMS-19879 (44590;690) | 47610 | 46130 | 48470 | 45630 | 47570 | 46100 | 48420 | 45550 | 3% |
| OxA-24944 (44600;2100) | 49850 | 45060 | 54890 | 44420 | 48200 | 45300 | 50250 | 44180 | 3% |
| MAMS-19857 (45020;1380) | 49000 | 45890 | 51960 | 45020 | 48200 | 45940 | 49850 | 45000 | 3% |
| MAMS-19853 (45300;1410) | 49420 | 46060 | 52370 | 45100 | 48300 | 46040 | 50070 | 45120 | 3% |
| Level D1 |  |  |  |  |  |  |  |  |  |
| Transition D2/D1 |  |  |  |  | 50630 | 47430 | 52930 | 46660 |  |
| TL ave Year-N(52900;1900) | 54860 | 50940 | 56700 | 49100 | 54270 | 50480 | 56210 | 48790 |  |
| Level D2 |  |  |  |  |  |  |  |  |  |
| Transition D3/D2 |  |  |  |  | 60140 | 50270 | 79140 | 48310 |  |
| Level D3 |  |  |  |  |  |  |  |  |  |
| Transition E/D3 |  |  |  |  | 78390 | 52260 | 120080 | 50110 |  |
| Level E |  |  |  |  |  |  |  |  |  |
| Start E |  |  |  |  | 161400 | 55460 | 161400 | 54100 |  |

**Table S5.** The calibrated and modelled radiocarbon dates from Stajnia Cave both from previous studies (OSL and U/Th are included) and obtained for this paper, calibrated with IntCal20^63^ in OxCal 4.4^62^. The pendant and the awl are in red, as well as the major outliers samples.

| **Geiβenklösterle (^14^C dates and stratigraphic information published in^69,70^)** | **Un-modelled (cal BP)** | | | | **Modelled (cal BP)** | | | | **Posterior outlier probability** |
| --- | --- | --- | --- | --- | --- | --- | --- | --- | --- |
| Indices Amodel 43.9 Aoverall 46.1 | **from** | **to** | **from** | **to** | **from** | **to** | **from** | **to** |  |
|  | **68.3%** | | **95.4%** | | **68.3%** | | **95.4%** | |  |
| End I Gravettian |  |  |  |  | 30920 | 29030 | 31680 | 25940 |  |
| OxA-21740 (26420;230) | 30930 | 30400 | 31080 | 30200 | 31010 | 30470 | 31230 | 30030 | 6% |
| OxA-21660 (27960;290) | 32770 | 31520 | 32970 | 31280 | 32800 | 31520 | 33040 | 31250 | 4% |
| OxA-21739 (28600;290) | 33270 | 32210 | 33770 | 31920 | 33290 | 32200 | 33850 | 31870 | 4% |
| OxA-21661 (32900;450) | 38250 | 36740 | 39050 | 36400 | 37800 | 36510 | 38790 | 36210 | 5% |
| I Gravettian |  |  |  |  |  |  |  |  |  |
| Transition Upper Aurignacian/Gravettian |  |  |  |  | 39310 | 37750 | 39690 | 36990 |  |
| OxA-21656 IIa (33000;500) | 38500 | 36880 | 39180 | 36430 | 39520 | 38340 | 40010 | 37490 | 9% |
| OxA-21737 IIa (35700;650) | 41370 | 40160 | 41910 | 39660 | 40520 | 39550 | 41120 | 39220 | 5% |
| OxA-21724 IIb (33950;550) | 39690 | 38070 | 40350 | 37320 | 39870 | 38770 | 40360 | 37950 | 3% |
| OxA-21727 IIb (34100;550) | 39920 | 38310 | 40520 | 37520 | 39950 | 38890 | 40460 | 38090 | 2% |
| OxA-21742 IIb (34800;600) | 40590 | 39400 | 41260 | 38620 | 40220 | 39300 | 40860 | 38830 | 2% |
| OxA-21738 IIb (34900;600) | 40660 | 39490 | 41360 | 38910 | 40250 | 39350 | 40900 | 38960 | 3% |
| II Upper Aurignacian |  |  |  |  |  |  |  |  |  |
| Transition III Early or Lower Aurignacian/II Upper Aurignacian |  |  |  |  | 41000 | 39910 | 41630 | 39540 |  |
| TL mean Year-N(40200;1500) | 41750 | 38650 | 43200 | 37200 | 42000 | 40740 | 42390 | 40090 |  |
| OxA-21722 III (38900;1000) | 43330 | 42130 | 44430 | 41870 | 42470 | 41810 | 42710 | 41240 | 9% |
| OxA-21744 III (36850;750) | 42130 | 41220 | 42460 | 40620 | 42060 | 41280 | 42320 | 40790 | 2% |
| OxA-21659 III (35050;600) | 40800 | 39640 | 41370 | 39150 | 41380 | 40350 | 41960 | 39990 | 7% |
| OxA-21726 IId (34200;550) | 40050 | 38480 | 40580 | 37600 | 41850 | 40120 | 42150 | 39790 | 32% |
| OxA-21725 III (37400;800) | 42370 | 41490 | 42780 | 40980 | 42200 | 41460 | 42450 | 41040 | 2% |
| OxA-21746 IIIa (36850;800) | 42150 | 41190 | 42520 | 40530 | 42060 | 41250 | 42330 | 40740 | 2% |
| OxA-21745 IIIa (36650;750) | 42060 | 41090 | 42400 | 40440 | 42010 | 41190 | 42280 | 40670 | 2% |
| OxA-21721 IIIb (37300;800) | 42320 | 41440 | 42730 | 40910 | 42170 | 41420 | 42430 | 40990 | 2% |
| OxA-21723 IIIb (37800;900) | 42640 | 41670 | 43250 | 41010 | 42290 | 41540 | 42530 | 41100 | 2% |
| OxA-21743 IIIb (36100;700) | 41780 | 40630 | 42130 | 39940 | 41860 | 40930 | 42150 | 40410 | 2% |
| III Early or Lower Aurignacian |  |  |  |  |  |  |  |  |  |
| Transition SterileIIIc/III Early or Lower Aurignacian |  |  |  |  | 42610 | 42070 | 42890 | 41700 |  |
| OxA-21658 (38300;900) | 42880 | 41970 | 43890 | 41400 | 42790 | 42250 | 43170 | 41950 | 2% |
| OxA-21657 (39400;1100) | 43860 | 42380 | 44810 | 42020 | 42860 | 42280 | 43360 | 41980 | 2% |
| IIIc Sterile |  |  |  |  |  |  |  |  |  |
| Transition IV/IIIc Sterile |  |  |  |  | 43170 | 42370 | 43960 | 42040 |  |
| ESR mean Year-N(42700;1300) | 44040 | 41360 | 45300 | 40100 | 43740 | 42570 | 44780 | 42180 |  |
| OxA-21720 (35500;650) | 41190 | 39990 | 41790 | 39480 | 43790 | 42510 | 45010 | 42070 | 96% |
| IV Middle Palaeolithic |  |  |  |  |  |  |  |  |  |
| Transition V/IV |  |  |  |  | 44630 | 42640 | 46750 | 42170 |  |
| V |  |  |  |  |  |  |  |  |  |
| Transition VI/V |  |  |  |  | 48100 | 43790 | 51130 | 42920 |  |
| VI |  |  |  |  |  |  |  |  |  |
| Transition VII/VI |  |  |  |  | 51360 | 46330 | 54010 | 44910 |  |
| OxA-21741 (48600;3200) | 54910 | 49800 | ... | 47130 | 52860 | 47820 | 55010 | 46730 | 5% |
| VII-Middle Palaeolithic |  |  |  |  |  |  |  |  |  |
| Start VII |  |  |  |  | 55010 | 48330 | 58580 | 46090 |  |

**Table S6.** Calibrated dates and Bayesian Modelled results of all the ^14^C ages obtained at Geiβenklösterle. Posterior outlier probabilities are also shown (coloured red when dates do not fit well with the stratigraphy). ^14^C dates are calibrated with IntCal20^63^ in OxCal 4.4^62^.

| **Vogelherd (^14^C dates and stratigraphic information published in^74-76^)** | **Un-modelled (cal BP)** | | | | **Modelled (cal BP)** | | | | **Posterior**  **outlier**  **probability** |
| --- | --- | --- | --- | --- | --- | --- | --- | --- | --- |
| Indices Amodel 0.2 Aoverall 0.4 | **from** | **to** | **from** | **to** | **from** | **to** | **from** | **to** |  |
|  | **68.3%** | | **95.4%** | | **68.3%** | | **95.4%** | |  |
| End III |  |  |  |  | 30270 | 28710 | 30640 | 26470 |  |
| OxA-10197 (39700;650) | 43800 | 42570 | 44230 | 42430 | 36110 | 32230 | 36250 | 29850 | 100% |
| OxA-10195 (31680;310) | 36320 | 35660 | 36670 | 35360 | 36070 | 35450 | 36440 | 29820 | 10% |
| OxA-10198 (26110;310) | 30740 | 30090 | 31040 | 29940 | 30770 | 30110 | 31100 | 29880 | 3% |
| OxA-10196 (25780;250) | 30360 | 29830 | 30750 | 29320 | 30390 | 29860 | 30850 | 29350 | 3% |
| KIA 19542 (29620;210) | 34390 | 33990 | 34540 | 33730 | 34400 | 33980 | 34650 | 30130 | 5% |
| III Aurignacian |  |  |  |  |  |  |  |  |  |
| Transition IV/III |  |  |  |  | 36340 | 35810 | 36680 | 30190 |  |
| H 4053-3211 (30730;750) | 35900 | 34480 | 36900 | 33850 | 36420 | 35940 | 36710 | 35490 | 2% |
| GrN-6583 (23860;190) | 28240 | 27780 | 28570 | 27710 | 36440 | 35960 | 36760 | 35450 | 100% |
| GrN-6662 (27630;830) | 32940 | 31070 | 34040 | 30350 | 36440 | 35950 | 36790 | 35380 | 87% |
| PL0001342A (34100;1100) | 40330 | 37630 | 41370 | 36530 | 36470 | 36010 | 36700 | 35630 | 19% |
| PL0003139A (32180;960) | 38020 | 35450 | 39410 | 34780 | 36440 | 35970 | 36680 | 35600 | 1% |
| OxA-18456 (32030;280) | 36690 | 36110 | 37030 | 35770 | 36430 | 36030 | 36630 | 35690 | 1% |
| KIA 8957 (26160;150) | 30730 | 30160 | 30830 | 30080 | 36440 | 35960 | 36750 | 35470 | 98% |
| IV Aurignacian |  |  |  |  |  |  |  |  |  |
| Transition V/IV |  |  |  |  | 36550 | 36100 | 36790 | 35820 |  |
| H 8499-8991 (31350;1120) | 37050 | 34540 | 39280 | 33950 | 36840 | 36260 | 37300 | 35970 | 2% |
| GrN-6661 (30650;560) | 35550 | 34500 | 36220 | 34190 | 36760 | 36200 | 37070 | 35890 | 14% |
| H 8500-8992 (30600;1700) | 37430 | 33170 | 40150 | 31680 | 36850 | 36260 | 37340 | 35960 | 3% |
| H 4054-3210 (30162;1340) | 36270 | 33110 | 38620 | 31700 | 36840 | 36260 | 37290 | 35950 | 3% |
| H 8497-8930 (27200;400) | 31650 | 31030 | 32100 | 30420 | 36860 | 36270 | 37370 | 35960 | 100% |
| H 8498-8950 (25900;260) | 30680 | 29930 | 30910 | 29660 | 36850 | 36260 | 37360 | 35960 | 100% |
| H 4055-3209 (23020;400) | 27740 | 26980 | 28000 | 26380 | 36860 | 36260 | 37340 | 35970 | 100% |
| H 4056-3208 (31900;1100) | 38020 | 35150 | 39530 | 34440 | 36850 | 36270 | 37330 | 35980 | 1% |
| PL0001337A (35810;710) | 41540 | 40250 | 42020 | 39680 | 36880 | 36270 | 37440 | 35980 | 100% |
| KIA 8970 (33080;320) | 38280 | 37060 | 39050 | 36850 | 37080 | 36380 | 37550 | 36090 | 12% |
| KIA 8969 (32500;260) | 37100 | 36460 | 37500 | 36260 | 36870 | 36370 | 37200 | 36150 | 2% |
| PL0001338A (32400;1700) | 39260 | 35380 | 41500 | 34240 | 36870 | 36270 | 37380 | 35990 | 2% |
| KIA 8968 (31790;240) | 36400 | 35870 | 36670 | 35520 | 36670 | 36210 | 36910 | 36030 | 2% |
| V Aurignacian |  |  |  |  |  |  |  |  |  |
| Transition VI/V |  |  |  |  | 37270 | 36450 | 37930 | 36110 |  |
| KIA 19541 (31310;240) | 35980 | 35440 | 36200 | 35240 | 38680 | 36370 | 42020 | 35990 | 93% |
| VI Middle Palaeolithic |  |  |  |  |  |  |  |  |  |
| Start VI |  |  |  |  | 41160 | 36200 | 55010 | 36080 |  |

**Table S7.** Calibrated dates and Bayesian Modelled results of all the ^14^C ages obtained at Vogelherd. Posterior outlier probabilities are also shown (coloured red when dates do not fit well with the stratigraphy). ^14^C dates are calibrated with IntCal20^63^ in OxCal 4.4^62^.

| **Abri Blanchard (^14^C dates and stratigraphic information published in^80^)** | **Un-modelled (cal BP)** | | | | **Modelled (cal BP)** | | | | **Posterior**  **outlier**  **probability** |
| --- | --- | --- | --- | --- | --- | --- | --- | --- | --- |
| Indices Amodel 99.4 Aoverall 100.4 | **from** | **to** | **from** | **to** | **from** | **to** | **from** | **to** |  |
|  | **68.3%** | | **95.4%** | | **68.3%** | | **95.4%** | |  |
| End Early Aurignacian |  |  |  |  | 39070 | 36330 | 39240 | 30880 |  |
| OxA-X-2669-55 (33960;360) | 39570 | 38470 | 39830 | 37660 | 39440 | 38330 | 39690 | 37620 | 4% |
| OxA-X-2669-54 (33420;350) | 38900 | 37640 | 39260 | 37150 | 39130 | 37790 | 39340 | 37330 | 4% |
| Aurignacian Phase |  |  |  |  |  |  |  |  |  |
| Start Early Aurignacian |  |  |  |  | 40970 | 38120 | 46430 | 37810 |  |

**Table S8.** Calibrated dates and Bayesian Modelled results of all the ^14^C ages obtained at Abri Blanchard. Posterior outlier probabilities are also shown (coloured red when dates do not fit well with the stratigraphy). ^14^C dates are calibrated with IntCal20^63^ in OxCal 4.4^62^.

| **Castanet Northern sector (^14^C dates and strati-graphic information published in^80^)** | **Un-modelled (cal BP)** | | | | **Modelled (cal BP)** | | | | **Posterior**  **outlier**  **probability** |
| --- | --- | --- | --- | --- | --- | --- | --- | --- | --- |
| Indices Amodel 143.3 Aoverall 144.9 | **from** | **to** | **from** | **to** | **from** | **to** | **from** | **to** |  |
|  | **68.3%** | | **95.4%** | | **68.3%** | | **95.4%** | |  |
| Boundary End 1 |  |  |  |  | 36650 | 36050 | 36910 | 35560 |  |
| OxA-21645 (32000;450) | 36930 | 35930 | 37480 | 35390 | 36810 | 36300 | 37090 | 36030 | 4% |
| OxA-21644 (32350;450) | 37220 | 36200 | 38340 | 35730 | 36830 | 36340 | 37170 | 36120 | 4% |
| OxA-21643 (32200;450) | 37080 | 36100 | 38050 | 35460 | 36820 | 36320 | 37140 | 36090 | 4% |
| OxA-21642 (32500;450) | 37430 | 36310 | 38650 | 36020 | 36850 | 36350 | 37220 | 36160 | 4% |
| OxA-21641 (31950;450) | 36860 | 35850 | 37400 | 35360 | 36800 | 36290 | 37090 | 36020 | 4% |
| OxA-21640 (31900;450) | 36770 | 35760 | 37320 | 35320 | 36800 | 36290 | 37080 | 35990 | 4% |
| OxA-21639 (32900;500) | 38320 | 36700 | 39110 | 36350 | 36880 | 36360 | 37330 | 36190 | 5% |
| Phase 1 |  |  |  |  |  |  |  |  |  |
| Boundary Start 1 |  |  |  |  | 37150 | 36480 | 37780 | 36310 |  |

| **Castanet Southern sector (^14^C dates and stratigraphic information published in^78^)** | **Un-modelled (cal BP)** | | | | **Modelled (cal BP)** | | | | **Posterior**  **outlier**  **probability** |
| --- | --- | --- | --- | --- | --- | --- | --- | --- | --- |
| Indices Amodel 67 Aoverall 69.9 | **from** | **to** | **from** | **to** | **from** | **to** | **from** | **to** |  |
|  | **68.3%** | | **95.4%** | | **68.3%** | | **95.4%** | |  |
| Boundary End 1 |  |  |  |  | 36860 | 36040 | 37100 | 35570 |  |
| GifA-99180 (32950;520) | 38450 | 36790 | 39170 | 36360 | 37460 | 36690 | 38100 | 36340 | 4% |
| GifA-99179 (32310;520) | 37360 | 36120 | 38540 | 35540 | 37240 | 36500 | 37700 | 36110 | 4% |
| GifA-99165 (31430;390) | 36160 | 35420 | 36590 | 34900 | 37010 | 36230 | 37290 | 35820 | 10% |
| GifA-99166 (34320;520) | 40290 | 38930 | 40640 | 37750 | 37850 | 36910 | 38580 | 36530 | 20% |
| GifA-97312 (32460;420) | 37300 | 36290 | 38440 | 36010 | 37230 | 36550 | 37640 | 36220 | 4% |
| GifA-97313 (32750;460) | 38010 | 36420 | 38940 | 36250 | 37380 | 36650 | 37940 | 36300 | 4% |
| OxA-21566 (32550;600) | 38010 | 36180 | 39130 | 35900 | 37330 | 36560 | 37950 | 36170 | 4% |
| OxA-21564 (32950;500) | 38430 | 36800 | 39150 | 36390 | 37460 | 36690 | 38100 | 36360 | 4% |
| OxA-21563 (32600;450) | 37560 | 36360 | 38760 | 36120 | 37310 | 36600 | 37800 | 36240 | 4% |
| OxA-21562 (32550;450) | 37500 | 36340 | 38710 | 36070 | 37280 | 36570 | 37760 | 36220 | 4% |
| OxA-21561 (32050;450) | 36970 | 35980 | 37540 | 35420 | 37110 | 36410 | 37490 | 36070 | 4% |
| OxA-21560 (32800;450) | 38040 | 36520 | 38960 | 36300 | 37410 | 36670 | 37980 | 36330 | 4% |
| OxA-21559 (33250;500) | 38850 | 37260 | 39380 | 36670 | 37590 | 36780 | 38240 | 36470 | 4% |
| OxA-21558 (32350;450) | 37220 | 36200 | 38340 | 35730 | 37210 | 36520 | 37620 | 36170 | 4% |
| Phase 1 |  |  |  |  |  |  |  |  |  |
| Boundary Start 1 |  |  |  |  | 38070 | 37020 | 38930 | 36740 |  |

**Table S9.** Calibrated dates and Bayesian Modelled results of all the ^14^C ages obtained at Abri de Castanet North and South. Posterior outlier probabilities are also shown (coloured red when dates do not fit well with the stratigraphy). ^14^C dates are calibrated with IntCal20^63^ in OxCal 4.4^62^.

| **La Souquette (^14^C dates and stratigraphic information published in^82^)** | **Un-modelled (cal BP)** | | | | **Modelled (cal BP)** | | | | **Posterior**  **outlier**  **probability** |
| --- | --- | --- | --- | --- | --- | --- | --- | --- | --- |
| Indices Amodel 106.5 Aoverall 109 | **from** | **to** | **from** | **to** | **from** | **to** | **from** | **to** |  |
|  | **68.3%** | | **95.4%** | | **68.3%** | | **95.4%** | |  |
| Boundary End Layer 11 |  |  |  |  | 36930 | 35790 | 37450 | 33840 |  |
| OxA-32198 (32150;450) | 37040 | 36070 | 37710 | 35430 | 37060 | 36290 | 37640 | 35900 | 4% |
| OxA-32198 (32400;500) | 37410 | 36200 | 38660 | 35720 | 37170 | 36340 | 37970 | 36010 | 4% |
| OxA-X-2627-47 (32400;550) | 37530 | 36160 | 38840 | 35680 | 37210 | 36340 | 38100 | 35980 | 4% |
| GifA-09456 (33710;1000) | 39720 | 37230 | 40910 | 36350 | 37530 | 36370 | 39020 | 36100 | 5% |
| Phase 1 |  |  |  |  |  |  |  |  |  |
| Boundary Start Layer 11 |  |  |  |  | 38170 | 36510 | 40750 | 36290 |  |

**Table S10.** Calibrated dates and Bayesian Modelled results of all the ^14^C ages obtained at Abri de la Souquette. Posterior outlier probabilities are also shown (coloured red when dates do not fit well with the stratigraphy). ^14^C dates are calibrated with IntCal20^63^ in OxCal 4.4^62^.

| **Tuto de Camalhot (^14^C dates and stratigraphic information published in^5,85^)** | **Un-modelled (cal BP)** | | | | **Modelled (cal BP)** | | | | **Posterior**  **outlier**  **probability** |
| --- | --- | --- | --- | --- | --- | --- | --- | --- | --- |
| Indices Amodel 93.1 Aoverall 92.7 | **from** | **to** | **from** | **to** | **from** | **to** | **from** | **to** |  |
|  | **68.3%** | | **95.4%** | | **68.3%** | | **95.4%** | |  |
| End Gravettian |  |  |  |  | 26820 | 24640 | 27680 | 22080 |  |
| Gif.-2941 (24200;600) | 29000 | 27800 | 29800 | 27360 | 28730 | 27730 | 29450 | 27260 | 4% |
| GRA14938 (24220;160) | 28680 | 28190 | 28770 | 27910 | 28630 | 28110 | 28750 | 27860 | 4% |
| GRA14939 (23380;150) | 27720 | 27420 | 27800 | 27310 | 27720 | 27420 | 27800 | 27310 | 4% |
| Gif 2942 (21500;400) | 26250 | 25300 | 26900 | 24950 | 26920 | 25560 | 27740 | 25020 | 9% |
| Gravettian |  |  |  |  |  |  |  |  |  |
| Transition Aurignacian/Gravettian |  |  |  |  | 30100 | 28120 | 34580 | 27880 |  |
| Gif.-99093 (35140;660) | 40930 | 39680 | 41610 | 39140 | 40810 | 39510 | 41660 | 38610 | 5% |
| Gif.-99674 (32180;570) | 37330 | 35950 | 38510 | 35360 | 37400 | 35940 | 38600 | 35350 | 5% |
| Aurignacian |  |  |  |  |  |  |  |  |  |
| Start Aurignacian |  |  |  |  | 58260 | 39520 | 58270 | 39110 |  |

**Table S11.** Calibrated dates and Bayesian Modelled results of all the ^14^C ages obtained at Tuto de Camalhot. Posterior outlier probabilities are also shown (coloured red when dates do not fit well with the stratigraphy). ^14^C dates are calibrated with IntCal20^63^ in OxCal 4.4^62^.

| **Sungir (^14^C dates and stratigraphic information published in^88^)** | **Un-modelled (cal BP)** | | | | **Modelled (cal BP)** | | | | **Posterior**  **outlier**  **probability** |
| --- | --- | --- | --- | --- | --- | --- | --- | --- | --- |
| Indices Amodel 95.4 Aoverall 97 | **from** | **to** | **from** | **to** | **from** | **to** | **from** | **to** |  |
|  | **68.3%** | | **95.4%** | | **68.3%** | | **95.4%** | |  |
| End Sungir 1-4 |  |  |  |  | 34390 | 32790 | 34540 | 29410 |  |
| OxA-X-2462-52 Sungir 4 (29820;280) | 34570 | 34100 | 34850 | 33720 | 34510 | 34090 | 34720 | 33770 | 4% |
| OxA-X-2464-12 Sungir 1 (28890;430) | 34020 | 32340 | 34280 | 32020 | 34400 | 33510 | 34580 | 32400 | 5% |
| Double Burial (30050;389) | 34910 | 34160 | 35340 | 33840 | 34650 | 34080 | 35150 | 33790 | 4% |
| Start Sungir 1-4 |  |  |  |  | 35350 | 34110 | 38680 | 33830 |  |

**Table S12.** Calibrated dates and Bayesian Modelled results of all the ^14^C ages obtained at Sungir. Posterior outlier probabilities are also shown (coloured red when dates do not fit well with the stratigraphy). ^14^C dates are calibrated with IntCal20^63^ in OxCal 4.4^62^.

| **Yana (^14^C dates and stratigraphic information published in^89-92^)** | **Un-modelled (BP)** | | | | **Modelled (BP)** | | | | **Posterior**  **outlier**  **probability** |
| --- | --- | --- | --- | --- | --- | --- | --- | --- | --- |
| Indices Amodel 101.9 Aoverall 101.7 | **from** | **to** | **from** | **to** | **from** | **to** | **from** | **to** |  |
|  | **68.3%** | | **95.4%** | | **68.3%** | | **95.4%** | |  |
| **Yana TUMS 1** |  | |  | |  | |  | |  |
| End TUMS1 14-13m |  |  |  |  | 10210 | 7330 | 10290 | 400 |  |
| LE-6447 (8960;80) | 10230 | 9910 | 10250 | 9770 | 10230 | 9920 | 10250 | 9770 | 4% |
| Phase 14-13m |  |  |  |  |  |  |  |  |  |
| Transition 10-9m/14-13m |  |  |  |  | 21800 | 9960 | 21890 | 9950 |  |
| LE-6445 (18100;340) | 22370 | 21470 | 22760 | 21000 | 22380 | 21480 | 22840 | 21030 | 5% |
| LE-6446 (22400;300) | 27080 | 26400 | 27250 | 26030 | 27080 | 26390 | 27250 | 26010 | 5% |
| Phase 10-09m |  |  |  |  |  |  |  |  |  |
| Transition 8-7m/10-9m |  |  |  |  | 31190 | 29430 | 31380 | 27430 |  |
| Beta-173067 (27300;270) | 31540 | 31130 | 31800 | 31030 | 31440 | 31080 | 31740 | 30930 | 5% |
| LE-6443 (26500;600) | 31140 | 30150 | 31840 | 29350 | 31260 | 30430 | 31660 | 29980 | 4% |
| LE-6444 (25900;750) | 30950 | 29350 | 31600 | 28730 | 31270 | 30220 | 31630 | 29330 | 5% |
| Phase 08-07m |  |  |  |  |  |  |  |  |  |
| Start TUMS1 8-7m |  |  |  |  | 32020 | 31110 | 34160 | 30830 |  |
| **Yana Northen Area (NP)** |  |  |  |  |  |  |  |  |  |
| End Northen Area (NP) 15-4m |  |  |  |  | 12860 | 10280 | 13230 | 5550 |  |
| Beta-223406 (11950;70) | 14020 | 13750 | 14040 | 13600 | 14020 | 13610 | 14040 | 13600 | 4% |
| LE-7615 (10590;300) | 12820 | 11960 | 13110 | 11400 | 12920 | 12100 | 13300 | 11600 | 5% |
| Phase 15-14m |  |  |  |  |  |  |  |  |  |
| Transition 14-13m/15-14m |  |  |  |  | 17010 | 13840 | 17030 | 13810 |  |
| Beta-243115 (14010;80) | 17260 | 16910 | 17350 | 16780 | 17250 | 16900 | 17350 | 16760 | 4% |
| Phase 14-13m |  |  |  |  |  |  |  |  |  |
| Transition 13-12m/14-13m |  |  |  |  | 19960 | 16990 | 21650 | 16960 |  |
| Beta-243116 (17970;100) | 22070 | 21730 | 22120 | 21440 | 22080 | 21730 | 22130 | 21440 | 4% |
| Phase 13-12m |  |  |  |  |  |  |  |  |  |
| Transition 9-8m/13-12m |  |  |  |  | 30980 | 30320 | 31160 | 29310 |  |
| Beta-191322 (28570;300) | 33240 | 32190 | 33750 | 31890 | 32650 | 31920 | 33240 | 31740 | 5% |
| Beta-191326 (28500;200) | 33080 | 32220 | 33370 | 31940 | 32640 | 32000 | 33130 | 31850 | 5% |
| Beta-191328 (27820;190) | 31960 | 31510 | 32720 | 31220 | 31960 | 31510 | 32190 | 31240 | 4% |
| Beta-191332 (27510;180) | 31610 | 31250 | 31780 | 31160 | 31610 | 31250 | 31790 | 31150 | 4% |
| Beta-223413 (27250;230) | 31480 | 31110 | 31700 | 31040 | 31480 | 31110 | 31710 | 31040 | 4% |
| Beta-191321 (27140;180) | 31310 | 31070 | 31590 | 31020 | 31320 | 31070 | 31600 | 31020 | 4% |
| Beta-191331 (26450;160) | 30960 | 30430 | 31060 | 30320 | 31060 | 30710 | 31120 | 30380 | 6% |
| Phase 09-08m |  |  |  |  |  |  |  |  |  |
| Transition 8-7m/9-8m |  |  |  |  | 33230 | 32240 | 33950 | 32050 |  |
| Beta-191330 (29610;230) | 34400 | 33960 | 34560 | 33670 | 34390 | 33950 | 34570 | 33640 | 4% |
| Phase 08-07m |  |  |  |  |  |  |  |  |  |
| Transition 3-2m/8-7m |  |  |  |  | 39610 | 34120 | 40220 | 33970 |  |
| Beta-204875 (34820;620) | 40630 | 39400 | 41310 | 38570 | 40580 | 39300 | 41250 | 38220 | 5% |
| Phase 03-02m |  |  |  |  |  |  |  |  |  |
| Start Northen Area (NP) 3-2m |  |  |  |  | 43720 | 39290 | 53090 | 38260 |  |
| **Yana B** |  |  |  |  |  |  |  |  |  |
| End Yana B 9-8m |  |  |  |  | 31980 | 31290 | 32200 | 29980 |  |
| Beta-250633 (28250;200) | 32850 | 31920 | 33060 | 31760 | 32250 | 31790 | 32820 | 31700 | 4% |
| Beta-250635 (28210;200) | 32840 | 31850 | 33030 | 31730 | 32220 | 31780 | 32800 | 31690 | 4% |
| Beta-250637 (28060;210) | 32760 | 31680 | 32950 | 31570 | 32140 | 31740 | 32750 | 31600 | 4% |
| Beta-250634 (27670;210) | 31770 | 31330 | 32010 | 31180 | 32020 | 31580 | 32190 | 31250 | 5% |
| Phase 09-08m |  |  |  |  |  |  |  |  |  |
| Start Yana B 9-8m |  |  |  |  | 32800 | 31870 | 34050 | 31750 |  |

**Table S13.** Calibrated dates and Bayesian Modelled results of all the ^14^C ages obtained at Yana. Posterior outlier probabilities are also shown (colored red when dates do not fit well with the stratigraphy). ^14^C dates are calibrated with IntCal20^63^ in OxCal 4.4^62^.

| **Abri Blanchard** | **Modelled (cal BP)** | | | |
| --- | --- | --- | --- | --- |
| *Indices: Amodel 99.4, Aoverall 100.4%* | **from** | **to** | **from** | **to** |
|  | **68.3%** | | **95.4%** | |
| End Early Aurignacian | 39070 | 36330 | 39240 | 30880 |
| **Early Aurignacian** | 39800 | 37360 | 43000 | 34190 |
| Start Early Aurignacian | 40970 | 38120 | 46430 | 37810 |
| **Abri de Castanet North** | **Modelled (cal BP)** | | | |
| *Indices: Amodel 143.3, Aoverall 144.9%* | **from** | **to** | **from** | **to** |
|  | **68.3%** | | **95.4%** | |
| End 1 | 36650 | 36050 | 36910 | 35560 |
| **Early Aurignacian** | 36880 | 36300 | 37360 | 35930 |
| Start 1 | 37150 | 36480 | 37780 | 36310 |
| **Abri de Castanet South** | **Modelled (cal BP)** | | | |
| *Indices: Amodel 67, Aoverall 69.9%* | **from** | **to** | **from** | **to** |
|  | **68.3%** | | **95.4%** | |
| End 1 | 36860 | 36040 | 37100 | 35570 |
| **Early Aurignacian** | 37430 | 36520 | 38190 | 36000 |
| Start 1 | 38070 | 37020 | 38930 | 36740 |
| **Abri de la Souquette** | **Modelled (cal BP)** | | | |
| *Indices: Amodel 106.5, Aoverall 109%* | **from** | **to** | **from** | **to** |
|  | **68.3%** | | **95.4%** | |
| End Layer 11 | 36930 | 35790 | 37450 | 33840 |
| **Aurignacian** | 37480 | 36240 | 39450 | 35020 |
| Start Layer 11 | 38170 | 36510 | 40750 | 36290 |
| **Geiβenklösterle** | **Modelled (cal BP)** | | | |
| *Indices: Amodel 43.9, Aoverall 46.1%* | **from** | **to** | **from** | **to** |
|  | **68.3%** | | **95.4%** | |
| Transition Upper Aurignacian/Gravettian | 39310 | 37750 | 39690 | 36990 |
| **Upper Aurignacian** | 40280 | 38800 | 40980 | 37790 |
| Transition III Early or Lower Aurignacian/II Upper Aurignacian | 41000 | 39910 | 41630 | 39540 |
|  |  |  |  |  |
| Transition III Early or Lower Aurignacian/II Upper Aurignacian | 41000 | 39910 | 41630 | 39540 |
| **Early or Lower Aurignacian** | 42170 | 40930 | 42500 | 40170 |
| Transition SterileIIIc/III Early or Lower Aurignacian | 42610 | 42070 | 42890 | 41700 |
|  |  |  |  |  |
| Transition IV/IIIc Sterile | 43170 | 42370 | 43960 | 42040 |
| **Middle Palaolithic** | 49130 | 43060 | 53780 | 42460 |
| Start VII | 55010 | 48330 | 58580 | 46090 |
| **Sungir** | **Modelled (cal BP)** | | | |
| *Indices: Amodel 95.4, Aoverall 97%* | **from** | **to** | **from** | **to** |
|  | **68.3%** | | **95.4%** | |
| End Sungir 1-4 | 34390 | 32790 | 34540 | 29410 |
| Sungir 1-4 | 34810 | 33500 | 36830 | 31190 |
| Start Sungir 1-4 | 35350 | 34110 | 38680 | 33830 |
|  |  |  |  |  |
| **Tuto de Camalhot** | **Modelled (cal BP)** | | | |
| *Indices: Amodel 93, Aoverall 92.7%* | **from** | **to** | **from** | **to** |
|  | **68.3%** | | **95.4%** | |
| End Gravettian | 26820 | 24640 | 27680 | 22080 |
| **Gravettian** | 28860 | 25920 | 31940 | 23640 |
| Transition Aurignacian/Gravettian | 30100 | 28120 | 34580 | 27880 |
|  |  |  |  |  |
| Transition Aurignacian/Gravettian | 30100 | 28120 | 34580 | 27880 |
| **Aurignacian** | 40790 | 30830 | 47720 | 28510 |
| Start Aurignacian | 58260 | 39520 | 58270 | 39110 |
| **Vogelherd** | **Modelled (cal BP)** | | | |
| *Indices: Amodel 0.2, Aoverall 0.4%* | **from** | **to** | **from** | **to** |
|  | **68.3%** | | **95.4%** | |
| End III | 30270 | 28710 | 30640 | 26470 |
| **Aurignacian** | 36280 | 30320 | 37180 | 28890 |
| Transition VI/V | 37270 | 36450 | 37930 | 36110 |
|  |  |  |  |  |
| Transition VI/V | 37270 | 36450 | 37930 | 36110 |
| **Middle Palaolithic** | 38970 | 36360 | 45450 | 35940 |
| Start VI | 41160 | 36200 | 55010 | 36080 |
| **Yana sites** | **Modelled (cal BP)** | | | |
| *Indices: Amodel 101.9, Aoverall 101.8%* | **from** | **to** | **from** | **to** |
| TUMS 1 | **68.3%** | | **95.4%** | |
| Transition 8-7m/10-9m | 31190 | 29430 | 31380 | 27430 |
| **Yana TUMS 1** | 31620 | 30300 | 32720 | 28530 |
| Start TUMS1 8-7m | 32020 | 31110 | 34160 | 30830 |
| Northern Area (NP) |  |  |  |  |
| Transition 9-8m/13-12m | 30980 | 30320 | 31160 | 29310 |
| **Yana Cultural Layer 9-8m** | 32400 | 30820 | 33330 | 30170 |
| Start Northen Area (NP) 3-2m | 33230 | 32240 | 33950 | 32050 |
| Yana B |  |  |  |  |
| End Yana B 9-8m | 31980 | 31290 | 32200 | 29980 |
| **Yana B 9-8m** | 32320 | 31590 | 33320 | 30840 |
| Start Yana B 9-8m | 32800 | 31870 | 34050 | 31750 |

**Table S14:** Boundaries and duration of the phases from the sites mentioned in the text (Stajnia is not considered) produced using the ‘date’ command in OxCal^62^. In red are the ranges used in Figure 3b in the main text.

| **Nr** | **Working technology** | **Raw material** | **Tool** | **Work effectiveness** | **Use-wear description** |
| --- | --- | --- | --- | --- | --- |
| 1 | Scraping | Fresh humerus (domestic cattle) | Flake edge | Tool edges were fully worn after 1-2 minutes of scraping. Bone surface hard to work | Varied linear traces, their width, depth, distribution and cross sections changed due to damages on the working edge of the flint tool. Their cross sections are generally V-shaped |
| 2 | Scraping | Fresh humerus (domestic cattle) | Blade edge | See above | See above |
| 3 | Scraping | Fresh humerus (domestic cattle) | Burin edge | Tool edge was fully worn after 4-5 minutes of scraping. Bone surface hard to work | The linear traces are more homogeneous than in the case when the bone was worked with flakes and blades, they are also more shallow |
| 4 | Scraping | Cooked humerus (domestic cattle) | Flake edge | Tool edges were fully worn after 1-2 minutes of scraping. Bone surface easier to work in comparison to fresh bones | Traces are analogous to those observed on fresh bones worked with flakes and blades but they are deeper and more apparent |
| 5 | Scraping | Cooked humerus (domestic cattle) | Blade edge | See above | See above |
| 6 | Scraping | Cooked humerus (domestic cattle) | Burin edge | Tool edges were fully worn after 8-12 minutes of scraping. Bone surface easier to work in comparison to fresh bones | Traces are analogous to those observed on fresh bones worked with flakes and blades but they are deeper and more apparent |
| 7 | Scraping | Cementum fragment of a mammoth tusk | Flake edge | In the initial phase the top layer of the cementum is very hard to remove, the working edge is fully worn after ca. 1 minute | Some places the surface is flaked off and crumbled away, the cross sections of the linear traces are vague, apparent fossilisation, differences between the colour of the traces and the colour of the surface are visible |
| 8 | Scraping | Cementum fragment of a mammoth tusk | Blade edge | See above | See above |
| 9 | Scraping | Cementum fragment of a mammoth tusk | Burin edge | In the initial phase the top layer of the cementum is very hard to remove, the working edge is fully worn after ca. 1-2 minutes | The linear traces are vague, more shallow than the scraping traces, some places the surface is crumbled away and flaked off, differences between the colour of the traces and the color of the surface are visible |
| 10 | Boring | Fresh humerus (domestic cattle) | Borer tip | The dots are made with multiple rotations of the tool but they are shallow because of the hardness of the bone, the time within which the tool becomes fully worn cannot be predicted, in a few cases it was possible to make 5 to 12 morphologically similar dots | Shallow double-level dots – the shape of the first level resembles straight dots and than on one of the sides a much shallower, flattened surface is located, the distal edge of which diverges radially in planum similar to oval, initially of a regular shape and concave cross section, with time and use of the tip they become more shallow and irregular |
| 11 | Boring | Fresh humerus (domestic cattle) | Flake tip (unretouched) | The dots are made with multiple rotations of the tool but they are shallow because of the hardness of the bone, the time within which the tool becomes fully worn cannot be predicted, in a few cases it was possible to make 2 to 4 morphologically similar dots | Shallow dots similar to oval in planum with frayed edges |
| 12 | Boring | Fresh humerus (domestic cattle) | Blade tip (unretouched) | See above | See above |
| 13 | Boring | Fresh humerus (domestic cattle) | Burin tip | The dots are made with a single rotation of the tool but they are shallow because of the hardness of the bone, the time within which the tool becomes fully worn cannot be predicted, in a few cases it was possible to make 5 to 12 morphologically similar dots | Shallow dots of homogeneous shape due to crumbling away of the tip of the tool, initially they resemble a circle, concave at one side, shaped in such a way that two small arms with sharp or rounded ends are visible, after the tip had crumbled away, the dots made were double-concave in plan, and concave with irregular bottoms and edges of the opening |
| 14 | Boring | Cooked humerus (domestic cattle) | Borer tip | The dots are made with multiple rotations of the tool, the number of repetitions which resulted in making similar dots was 20 to 50 | Shallow double-level dots – the shape of the first level resembles straight dots and than on one of the sides a much shallower, flattened surface is located, the distal edge of which diverges radially, the dots are deeper than in case of the fresh bone |
| 15 | Boring | Cooked humerus (domestic cattle) | Flake tip (unretouched) | The dots are made with multiple rotations of the tool but they are shallow because of the hardness of the bone, the time within which the tool becomes fully worn cannot be predicted, in a few cases it was possible to make 2 to 4 morphologically similar dots | Shallow dots s, in planum similar to ovals, with frayed edges |
| 16 | Boring | Fresh humerus (domestic cattle) | Blade tip (unretouched) | See above | See above |
| 17 | Boring | Cooked humerus (domestic cattle) | Burin tip | The dots are very easily made with a single rotation of the tool but they are shallow because of the hardness of the bone, the time within which the flint tool becomes fully worn cannot be predicted, in some cases it was possible to make 30 to 40 dots, and other times the tip crumbled and broke already after 4-8 uses | dots resemble a circle in plan, concave at one side, shaped in such a way that two small arms with sharp or rounded ends are visible |
| 18 | Boring | Cementum fragment of a mammoth tusk | Borer tip | Initially the tip of the tool is very hard to press into the surface of the tooth; the tooth crumbles and cracks in the area where the opening is made. The pressure made the cementum fragments fall apart in a few cases. | Due to the fossilisation and mineralisation of the tooth technological traces are hard to identify but for the concavity made by boring |
| 19 | Boring | Cementum fragment of a mammoth tusk | Flake tip (unretouched) | See above | See above |
| 20 | Boring | Cementum fragment of a mammoth tusk | Burin tip | See above | See above |
| 21 | Indirect percussion | Fresh humerus (domestic cattle) | Borer tip | The tip of the tool crumbles away after 5-12 strikes | Shallow dents are made, shapes are similar to circles with concave bottoms |
| 22 | Indirect percussion | Fresh humerus (domestic cattle) | Flake tip (unretouched) | The tip of the tool crumbles away after 1-3 strikes | The shapes of the dots are irregular, sharp on the edges, shallow |
| 23 | Indirect percussion | Fresh humerus (domestic cattle) | Burin tip | See above | See above |
| 24 | Indirect percussion | Cooked humerus (domestic cattle) | Borer tip | The tip of the tool crumbles less than when working on a fresh bone | The shapes of the dots are irregular, close to round and oval, sharp on the edges, slightly deeper than in the case of the worked fresh bone |
| 25 | Indirect percussion | Cooked humerus (domestic cattle) | Blade tip (unretouched) | The tip of the tool crumbles away after 1-3 strikes | The shapes of the dots are irregular, sharp on the edges, slightly deeper than in the case of the worked fresh bone |
| 26 | Indirect percussion | Cooked humerus (domestic cattle) | Burin tip | See above | See above |
| 27 | Indirect percussion | Cementum fragment of a mammoth tusk | Borer tip | Each time the cementum fell apart into a couple of pieces | Some points on the edges of the split cementum fragments have crumbled away |
| 28 | Indirect percussion | Cementum fragment of a mammoth tusk | Flake point (unretouched) | See above | See above |
| 29 | Indirect percussion | Cementum fragment of a mammoth tusk | Blade tip (unretouched) | See above | See above |
| 30 | Indirect percussion | Cementum fragment of a mammoth tusk | Burin tip | See above | See above |

**Table S15:** Description of the conducted experiments. Nr – number of the experiment.

**CQL model codes**

The sites are in alphabetic order

**Abri Blanchard**

**^14^C dates published in^78^**

**Bayesian Model from^80^**

Plot()

{

Outlier_Model("General",T(5),U(0,4),"t");

Sequence()

{

Boundary("Start Early Aurignacian");

Phase("1")

{

R_Date("OxA-X-2669-54", 33420, 350)

{

Outlier(0.05);

};

R_Date("OxA-X-2669-55", 33960, 360)

{

Outlier(0.05);

};

};

Boundary("End Early Aurignacian");

};

Sequence()

{

Boundary("=Start Early Aurignacian");

Date("Early Aurignacian");

Boundary("=End Early Aurignacian");

};

};

**Abri de Castanet North**

**^14^C dates published in^81^**

**Bayesian Model from^80^**

Plot()

{

Outlier_Model("General",T(5),U(0,4),"t");

Sequence()

{

Boundary("Start 1");

Phase("1")

{

R_Date("OxA-21639", 32900, 500)

{

Outlier(0.05);

};

R_Date("OxA-21640", 31900, 450)

{

Outlier(0.05);

};

R_Date("OxA-21641*", 31950, 450)

{

Outlier(0.05);

};

R_Date("OxA-21642*", 32500, 450)

{

Outlier(0.05);

};

R_Date("OxA-21643", 32200, 450)

{

Outlier(0.05);

};

R_Date("OxA-21644", 32350, 450)

{

Outlier(0.05);

};

R_Date("OxA-21645", 32000, 450)

{

Outlier(0.05);

};

};

Boundary("End 1");

};

Sequence()

{

Boundary("=Start 1");

Date("Early Aurignacian");

Boundary("=End 1");

};

};

**Abri de Castanet South**

**^14^C dates published in^78^**

**Bayesian Model from^80^**

Plot()

{

Outlier_Model("General",T(5),U(0,4),"t");

Sequence()

{

Boundary("Start 1");

Phase("1")

{

R_Date("OxA-21558", 32350, 450)

{

Outlier(0.05);

};

R_Date("OxA-21559", 33250, 500)

{

Outlier(0.05);

};

R_Date("OxA-21560", 32800, 450)

{

Outlier(0.05);

};

R_Date("OxA-21561", 32050, 450)

{

Outlier(0.05);

};

R_Date("OxA-21562", 32550, 450)

{

Outlier(0.05);

};

R_Date("OxA-21563", 32600, 450)

{

Outlier(0.05);

};

R_Date("OxA-21564", 32950, 500)

{

Outlier(0.05);

};

R_Date("OxA-21566", 32550, 600)

{

Outlier(0.05);

};

R_Date("GifA-97313", 32750, 460)

{

Outlier(0.05);

};

R_Date("GifA-97312", 32460, 420)

{

Outlier(0.05);

};

R_Date("GifA-99166", 34320, 520)

{

Outlier(0.05);

};

R_Date("GifA-99165", 31430, 390)

{

Outlier(0.05);

};

R_Date("GifA-99179", 32310, 520)

{

Outlier(0.05);

};

R_Date("GifA-99180", 32950, 520)

{

Outlier(0.05);

};

};

Boundary("End 1");

};

Sequence()

{

Boundary("=Start 1");

Date("Early Aurignacian");

Boundary("=End 1");

};

};

**Abri de la Souquette**

**^14^C dates published in^82^**

**Bayesian Model from this study**

Plot()

{

Outlier_Model("General",T(5),U(0,4),"t");

Sequence()

{

Boundary("Start 1");

Phase("1")

{

R_Date("GifA-09456", 33710, 1000)

{

Outlier(0.05);

};

R_Date("OxA-X-2627-47", 32400, 550)

{

Outlier(0.05);

};

R_Date("OxA-32198-bone 201", 32400, 500)

{

Outlier(0.05);

};

R_Date("OxA-32198-bone 68", 32150, 450)

{

Outlier(0.05);

};

};

Boundary("End 1");

};

Sequence()

{

Boundary("=Start 1");

Date("Aurignacian");

Boundary("=End 1");

};

};

**Geiβenklösterle**

**^14^C dates and Bayesian Model from^69,70^**

Plot()

{

Outlier_Model("General",T(5),U(0,4),"t");

Sequence()

{

Boundary("Start VII");

Phase("VII-Middle Palaeolithic")

{

R_Date("OxA-21741", 48600, 3200)

{

Outlier(0.05);

};

};

Boundary("Transition VII/VI");

Phase("VI")

{

};

Boundary("Transition VI/V");

Phase("V")

{

};

Boundary("Transition V/IV");

Phase("IV Middle Palaeolithic")

{

R_Date("OxA-21720", 35500, 650)

{

Outlier(0.05);

};

Age("ESR mean", N( 42700, 1300))

{

color="red";

Outlier(0.05);

};

};

Boundary("Transition IV/IIIc Sterile");

Phase("IIIc Sterile")

{

R_Date("OxA-21657", 39400, 1100)

{

Outlier(0.05);

};

R_Date("OxA-21658", 38300, 900)

{

Outlier(0.05);

};

};

Boundary("Transition SterileIIIc/III Early or Lower Aurignacian");

Phase("III Early or Lower Aurignacian")

{

R_Date("OxA-21743 IIIb", 36100, 700)

{

Outlier(0.05);

};

R_Date("OxA-21723 IIIb", 37800, 900)

{

Outlier(0.05);

};

R_Date("OxA-21721 IIIb", 37300, 800)

{

Outlier(0.05);

};

R_Date("OxA-21745 IIIa", 36650, 750)

{

Outlier(0.05);

};

R_Date("OxA-21746 IIIa", 36850, 800)

{

Outlier(0.05);

};

R_Date("OxA-21725 III", 37400, 800)

{

Outlier(0.05);

};

R_Date("OxA-21726 IId", 34200, 550)

{

Outlier(0.05);

};

R_Date("OxA-21659 III", 35050, 600)

{

Outlier(0.05);

};

R_Date("OxA-21744 III", 36850, 750)

{

Outlier(0.05);

};

R_Date("OxA-21722 III", 38900, 1000)

{

Outlier(0.05);

};

Age("TL mean", N( 40200, 1500))

{

color="red";

Outlier(0.05);

};

};

Boundary("Transition III Early or Lower Aurignacian/II Upper Aurignacian");

Phase("II Upper Aurignacian")

{

R_Date("OxA-21738 IIb", 34900, 600)

{

Outlier(0.05);

};

R_Date("OxA-21742 IIb", 34800, 600)

{

Outlier(0.05);

};

R_Date("OxA-21727 IIb", 34100, 550)

{

Outlier(0.05);

};

R_Date("OxA-21724 IIb", 33950, 550)

{

Outlier(0.05);

};

R_Date("OxA-21737 IIa", 35700, 650)

{

Outlier(0.05);

};

R_Date("OxA-21656 IIa", 33000, 500)

{

Outlier(0.05);

};

};

Boundary("Transition Upper Aurignacian/Gravettian");

Phase("I Gravettian")

{

R_Date("OxA-21661", 32900, 450)

{

Outlier(0.05);

};

R_Date("OxA-21739", 28600, 290)

{

Outlier(0.05);

};

R_Date("OxA-21660", 27960, 290)

{

Outlier(0.05);

};

R_Date("OxA-21740", 26420, 230)

{

Outlier(0.05);

};

};

Boundary("End I Gravettian");

};

Sequence()

{

Boundary("=Start VII");

Date("Middle palaolithic");

Boundary("=Transition IV/IIIc Sterile");

};

Sequence()

{

Boundary("=Transition SterileIIIc/III Early or Lower Aurignacian");

Date("Early or Lower Aurignacian");

Boundary("=Transition III Early or Lower Aurignacian/II Upper Aurignacian");

};

Sequence()

{

Boundary("=Transition III Early or Lower Aurignacian/II Upper Aurignacian");

Date("Upper Aurignacian");

Boundary("=Transition Upper Aurignacian/Gravettian");

};

};

**Stajnia**

**^14^C dates and Bayesian Model from^4^ and this study**

Plot()

{

Outlier_Model("General",T(5),U(0,4),"t");

Sequence("Stajnia Cave")

{

Boundary("Start E");

Phase("Level E")

{

};

Boundary("Transition E/D3");

Phase("Level D3")

{

};

Boundary("Transition D3/D2");

Phase("Level D2")

{

Age("TL ave", N(52900,1900))

{

Outlier(0.05);

};

};

Boundary("Transition D2/D1");

Phase("Level D1")

{

R_Date("MAMS-19853", 45300, 1410)

{

Outlier(0.05);

};

R_Date("MAMS-19857", 45020, 1380)

{

Outlier(0.05);

};

R_Date("OxA-24944", 44600, 2100)

{

Outlier(0.05);

};

R_Date("MAMS-19879", 44590, 690)

{

Outlier(0.05);

};

R_Combine("Awl")

{

color="red";

Outlier(0.05);

R_Date(" MAMS-35152", 37360, 330);

R_Date(" ETH-99042.1.1", 37903, 267);

};

R_Combine("Pendant")

{

color="red";

Outlier(0.05);

R_Date(" MAMS-35153 ", 36600, 300);

R_Date(" ETH-99043.1.1", 36563, 229);

};

};

Boundary("Transition D1/C19");

Phase("Level C19")

{

R_Date("MAMS-19864", 37750, 310)

{

Outlier(0.05);

};

R_Date("MAMS-19851", 36080, 460)

{

Outlier(0.05);

};

R_Date("MAMS-19849", 33450, 350)

{

Outlier(0.05);

};

};

Boundary("Transition C19/18");

Phase("Level C18")

{

R_Date("MAMS-19870", 40400, 420)

{

Outlier(0.05);

};

R_Date("GdA-3894", 21900, 90)

{

Outlier(0.05);

};

R_Date("Poz-61719", 20930, 140)

{

Outlier(0.05);

};

};

Boundary("Transition C18/B");

Phase("Level B")

{

R_Date("Poz-28891", 13500, 50)

{

Outlier(0.05);

};

};

Boundary("End layer B");

};

};

**Sungir**

**^14^C dates published in^88^**

**Bayesian Model from^88^**

Plot()

{

Outlier_Model("General",T(5),U(0,4),"t");

Sequence()

{

Boundary("Start Sungir 1-4");

Phase("1")

{

R_Combine("Double Burial")

{

Outlier(0.05);

R_Date("OxA-X-2395-6 Sungir 2", 30100, 550);

R_Date("OxA-X-2395-7 Sungir 3", 30000, 550);

};

R_Date("OxA-X-2464-12 Sungir 1", 28890, 430)

{

Outlier(0.05);

};

R_Date("OxA-X-2462-52 Sungir 4", 29820, 280)

{

Outlier(0.05);

};

};

Boundary("End Sungir 1-4");

};

Sequence()

{

Boundary("=Start Sungir 1-4");

Date("Sungir 1-4");

Boundary("=End Sungir 1-4");

};

};

**Tuto de Camalhot**

**^14^C dates published in^5,85^**

**Bayesian Model from this study**

Plot()

{

Outlier_Model("General",T(5),U(0,4),"t");

Sequence()

{

Boundary("Start Aurignacian");

Phase("Aurignacian")

{

R_Date("Gif.-99674", 32180, 570)

{

Outlier(0.05);

};

R_Date("Gif.-99093", 35140, 660)

{

Outlier(0.05);

};

};

Boundary("Transition Aurignacian/Gravettian");

Phase("Gravettian")

{

R_Date("Gif 2942", 21500, 400)

{

Outlier(0.05);

};

R_Date("GRA14939", 23380, 150)

{

Outlier(0.05);

};

R_Date("GRA14938", 24220, 160)

{

Outlier(0.05);

};

R_Date("Gif.-2941", 24200, 600)

{

Outlier(0.05);

};

};

Boundary("End Gravettian");

};

Sequence()

{

Boundary("=Start Aurignacian");

Date("Aurignacian");

Boundary("=Transition Aurignacian/Gravettian");

};

Sequence()

{

Boundary("=Transition Aurignacian/Gravettian");

Date("Gravettian");

Boundary("=End Gravettian");

};

};

**Vogelherd**

**^14^C dates published in^75,76^**

**Bayesian Model from this study**

Plot()

{

Outlier_Model("General",T(5),U(0,4),"t");

Sequence()

{

Boundary("Start VI");

Phase("VI Middle Palaeolithic")

{

R_Date("KIA 19541", 31310, 240)

{

color="red";

Outlier(0.05);

};

};

Boundary("Transition VI/V");

Phase("V Aurignacian")

{

R_Date("KIA 8968", 31790, 240)

{

color="red";

Outlier(0.05);

};

R_Date("PL0001338A", 32400, 1700)

{

color="red";

Outlier(0.05);

};

R_Date("KIA 8969", 32500, 260)

{

color="red";

Outlier(0.05);

};

R_Date("KIA 8970", 33080, 320)

{

color="red";

Outlier(0.05);

};

R_Date("PL0001337A", 35810, 710)

{

color="red";

Outlier(0.05);

};

R_Date("H 4056-3208", 31900, 1100)

{

color="purple";

Outlier(0.05);

};

R_Date("H 4055-3209", 23020, 400)

{

color="purple";

Outlier(0.05);

};

R_Date("H 8498-8950", 25900, 260)

{

color="purple";

Outlier(0.05);

};

R_Date("H 8497-8930", 27200, 400)

{

color="purple";

Outlier(0.05);

};

R_Date("H 4054-3210", 30162, 1340)

{

color="purple";

Outlier(0.05);

};

R_Date("H 8500-8992", 30600, 1700)

{

color="purple";

Outlier(0.05);

};

R_Date("GrN-6661", 30650, 560)

{

color="purple";

Outlier(0.05);

};

R_Date("H 8499-8991", 31350, 1120)

{

color="purple";

Outlier(0.05);

};

};

Boundary("Transition V/IV");

Phase("IV Aurignacian")

{

R_Date("KIA 8957", 26160, 150)

{

color="red";

Outlier(0.05);

};

R_Date("OxA-18456", 32030, 280)

{

Outlier(0.05);

};

R_Date("PL0003139A", 32180, 960)

{

color="red";

Outlier(0.05);

};

R_Date("PL0001342A", 34100, 1100)

{

color="red";

Outlier(0.05);

};

R_Date("GrN-6662", 27630, 830)

{

color="purple";

Outlier(0.05);

};

R_Date("GrN-6583", 23860, 190)

{

color="purple";

Outlier(0.05);

};

R_Date("H 4053-3211", 30730, 750)

{

color="purple";

Outlier(0.05);

};

};

Boundary("Transition IV/III");

Phase("III Aurignacian")

{

R_Date("KIA 19542", 29620, 210)

{

color="red";

Outlier(0.05);

};

R_Date("OxA-10196", 25780, 250)

{

color="red";

Outlier(0.05);

};

R_Date("OxA-10198", 26110, 310)

{

color="red";

Outlier(0.05);

};

R_Date("OxA-10195", 31680, 310)

{

color="red";

Outlier(0.05);

};

R_Date("OxA-10197", 39700, 650)

{

color="red";

Outlier(0.05);

};

};

Boundary("End III");

};

Sequence()

{

Boundary("=Start VI");

Date("Middle palaolithic");

Boundary("=Transition VI/V");

};

Sequence()

{

Boundary("=Transition VI/V");

Date("Aurignacian");

Boundary("=End III");

};

};

**Yana**

**^14^C dates published in^89-92^**

**Bayesian Model from this study**

Plot()

{

Outlier_Model("General",T(5),U(0,4),"t");

Sequence("Yana B")

{

Boundary("Start Yana B 9-8m");

Phase("9-8")

{

R_Date("Beta-250634", 27670, 210)

{

Outlier(0.05);

};

R_Date("Beta-250637", 28060, 210)

{

Outlier(0.05);

};

R_Date("Beta-250635", 28210, 200)

{

Outlier(0.05);

};

R_Date("Beta-250633", 28250, 200)

{

Outlier(0.05);

};

};

Boundary("End Yana B 9-8m");

};

Sequence("Yana Northen Area (NP)")

{

Boundary("Start Northen Area (NP) 3-2m");

Phase("3-2")

{

R_Date("Beta-204875", 34820, 620)

{

Outlier(0.05);

};

};

Boundary("Transition 3-2m/8-7m");

Phase("8-7")

{

R_Date("Beta-191330", 29610, 230)

{

Outlier(0.05);

};

};

Boundary("Transition 8-7m/9-8m");

Phase("9-8")

{

R_Date("Beta-191331", 26450, 160)

{

Outlier(0.05);

};

R_Date("Beta-191321", 27140, 180)

{

Outlier(0.05);

};

R_Date("Beta-223413", 27250, 230)

{

Outlier(0.05);

};

R_Date("Beta-191332", 27510, 180)

{

Outlier(0.05);

};

R_Date("Beta-191328", 27820, 190)

{

Outlier(0.05);

};

R_Date("Beta-191326", 28500, 200)

{

Outlier(0.05);

};

R_Date("Beta-191322", 28570, 300)

{

Outlier(0.05);

};

};

Boundary("Transition 9-8m/13-12m");

Phase("13-12")

{

R_Date("Beta-243116", 17970, 100)

{

Outlier(0.05);

};

};

Boundary("Transition 13-12m/14-13m");

Phase("14-13")

{

R_Date("Beta-243115", 14010, 80)

{

Outlier(0.05);

};

};

Boundary("Transition 14-13m/15-14m");

Phase("15-14")

{

R_Date("LE-7615", 10590, 300)

{

Outlier(0.05);

};

R_Date("Beta-223406", 11950, 70)

{

Outlier(0.05);

};

};

Boundary("End Northen Area (NP) 15-4m");

};

Sequence("Yana TUMS1")

{

Boundary("Start TUMS1 8-7m");

Phase("8-7")

{

R_Date("LE-6444", 25900, 750)

{

Outlier(0.05);

};

R_Date("LE-6443", 26500, 600)

{

Outlier(0.05);

};

R_Date("Beta-173067", 27300, 270)

{

Outlier(0.05);

};

};

Boundary("Transition 8-7m/10-9m");

Phase("10-9")

{

R_Date("LE-6446", 22400, 300)

{

Outlier(0.05);

};

R_Date("LE-6445", 18100, 340)

{

Outlier(0.05);

};

};

Boundary("Transition 10-9m/14-13m");

Phase("14-13")

{

R_Date("LE-6447", 8960, 80)

{

Outlier(0.05);

};

};

Boundary("End TUMS1 14-13m");

};

Sequence("Yana B")

{

Boundary("=Start Yana B 9-8m");

Date("Yana B 9-8");

Boundary("=End Yana B 9-8m");

};

Sequence("Northen Area (NP)")

{

Boundary("=Transition 8-7m/9-8m");

Date("Yana Cultural Layer 9-8m");

Boundary("=Transition 9-8m/13-12m");

};

Sequence("TUMS 1")

{

Boundary("=Start TUMS1 8-7m");

Date("Yana B 9-8m");

Boundary("=Transition 8-7m/10-9m");

};

};

SI References

1 Żarski, M. *et al.* Stratigraphy and palaeoenvironment of Stajnia Cave (southern Poland) with regard to habitation of the site by Neanderthals. *Geological Quarterly* **61**, 350-369, doi: 310.7306/gq. 1355 (2017).

2 Erdtman, G. The acetolysis method-a revised description. *Sven Bot Tidskr* **54**, 516-564 (1960).

3 Noryśkiewicz, A., Filbrandt-Czaja, A., Noryśkiewicz, M. & D. Nalepka, D. in *Late Glacial and Holocene History of vegetation in Poland based on isopollen maps* (eds M. Ralska-Jasiewiczowa *et al.*) 305–308 (W. Szafer Institute of Botany, Polish Academy of Sciences, 2004).

4 Picin, A. *et al.* New perspectives on Neanderthal dispersal and turnover from Stajnia Cave (Poland). *Scientific Reports* **10**, 14778, doi:10.1038/s41598-020-71504-x (2020).

5 Bon, F. *L'Aurignacien entre mer et océan: réflexion sur l'unité des phases anciennes de l'Aurignacien dans le Sud de la France*. Vol. 29 (Société préhistorique française, 2002).

6 Chiotti, L. *Les industries lithiques des niveaux aurignaciens de l'abri Pataud, Dordogne, France. Les foullies de Hallam L. Movius Jr.*, (BAR International Series 1392, 2005).

7 Teyssandier, N. Revolution or evolution: the emergence of the Upper Paleolithic in Europe. *World Archaeology* **40**, 493-519, doi:10.1080/00438240802452676 (2008).

8 Sitlivy, V., Zieba, A. & Sobczyk, K. *Middle and Early Upper Palaeolithic of the Krakow Region Ksiecia Jozefa*. (Musées royaux d'art et d'histoire, 2009).

9 Sachse-Kozłowska, E. & Kozlowski, S. K. *Piekary près de Cracovie (Pologne): complexe des sites paléolithiques*. (Polska Akad. Umiejętności, 2004).

10 Sitlivy, V., Zieba, A. & Sobczyk, K. *Middle and Early Upper Palaeolithic of the Krakow Region Piekary IIa*. (Musées royaux d'art et d'histoire, 2008).

11 Teyssandier, N. & Liolios, D. in *The Chronology of the Aurignacian and of the Transitional Technocomplexes: Dating, Stratigraphies, Cultural Implications.* (eds J. Zilhão & Francesco D'Errico) 179-196 (Trabalhos de Arqueologia, vol. 33, Instituto Português de Arqueologia, 2003).

12 Karlin, C. in *Foullies de Pincevent. Essai d'analyse ethnographique d'un habitat Magdalénian. VII Supplément à Gallia Préhistoire* Vol. 18 (eds A. Leroi-Gourhan & Michel N Brézillon) 263-278 (CNRS, 1972).

13 Pelegrin, J. in *L’Europe Centrale et Septentrionale au Tardiglaciaire. Confrontation des modèles régionaux de peuplement. Actes de la Table-ronde internationale de Nemours 14, 15, 16 mai 1997. Mémoires du Musée de Préhistoire d’Ile-de-France n°7* Vol. 7 (eds B. Valentin, P. Bodu, & M. Christensen) 73-86 (A.P.R.A.I.F, 2000).

14 Wiśniewski, A., Połtowicz-Bobak, M., Bobak, D., Jary, Z. & Moska, P. The Epigravettian and the Magdalenian in Poland: New chronological data and an old problem. *Geochronometria* **44**, 16, doi:https://doi.org/10.1515/geochr-2015-0052 (2017).

15 Pyżewicz, K., Migal, W. & Grużdź, W. in *Lateglacial and Postglacial Pioneers in Northern Europe* Vol. BAR International Series 2599 (eds Felix Riede & M. Tallaavaara) 67-78 (Archeopress, 2014).

16 Sachse-Kozłowska, E. in *Aurignacien et Gravettien en Europe II: Cracovie-Nitra 1980* 172-182 (ERAUL Etudes et Recherches Archéologiques de l'Université de Liège, 1982).

17 Schild, R. *A Late Magdalenian Winter Hunting Camp in Southern Poland*. (Polish Academy of Sciences, 2014).

18 Wiśniewski, A. & Wilczyński, J. in *The Past Societies 1. Polish Lands from the first evidence of human presence to the early Middles Ages* Vol. 98-110 (eds P. Urbańczyk & J. Kabaciński) (Institute of Archaeology and Ethnology, Polish Academy of Sciences, 2017).

19 Kozłowski, J. K. & Kozłowski, S. K. *Le paléolithique en Pologne*. (Editions Jérôme Millon, 1996).

20 Masojć, M. & Bronowicki, J. The Chełm Massif area–An Aurignacian settlement enclave in the South-Western Poland. *Przegląd Archeologiczny* **51**, 49-76 (2003).

21 Sachse-Kozłowska, M. Polish Aurignacian assemblages. *Folia Quaternaria*, 37-69 (1978).

22 Chmielewski, W. *Civilisation de Jerzmanowice*. (IHKM PAN, 1961).

23 Kozłowski, J. K. Châtelperronien, Uluzzien et quoi plus à l'Est? *Anthropologie* **38**, 249-259 (2000).

24 Połtowicz-Bobak, M., Bobak, D., Badura, J., Wacnik, A. & Cywa, K. in *Le Paléolithique supérieur ancien de l’Europe du Nord-Ouest* (eds P. Bodu *et al.*) 485-496 (Société préhistorique française, 2013).

25 Valde-Nowak, P. & Nadachowski, A. Micoquian assemblage and environmental conditions for the Neanderthals in Obłazowa Cave, Western Carpathians, Poland. *Quatern. Int.* **326–327**, 146-156, doi:http://dx.doi.org/10.1016/j.quaint.2013.08.057 (2014).

26 Stefański, D. in *Multa per gentes et multa per saecula* (eds Pawel Valde-Nowak, K. Sobczyk, M. Nowak, & J. Źrałka) 63-70 (2018).

27 Kozłowski, J. K. Le paléolithique supérieur en Pologne. *L'Anthropologie (Paris)* **87**, 49-82 (1983).

28 Demidenko, Y. E., Škrdla, P. & Nejman, L. Aurignacian in Moravia. New geochronological, lithic and settlement data. *Památky archeologické* **108**, 5-38 (2017).

29 Sitlivy, V. in *Paleoanthropology of the Balkans and Anatolia: Human Evolution and its Context* (eds Katerina Harvati & Mirjana Roksandic) 229-265 (Springer Netherlands, 2016).

30 Alex, B., Valde-Nowak, P., Regev, L. & Boaretto, E. Late Middle Paleolithic of Southern Poland: Radiocarbon dates from Ciemna and Obłazowa Caves. *Journal of Archaeological Science: Reports* **11**, 370-380, doi:https://doi.org/10.1016/j.jasrep.2016.12.012 (2017).

31 Bobak, D., Płonka, T., Połtowicz-Bobak, M. & Wiśniewski, A. New chronological data for Weichselian sites from Poland and their implications for Palaeolithic. *Quatern. Int.* **296**, 23-36, doi:https://doi.org/10.1016/j.quaint.2012.12.001 (2013).

32 Krajcarz, M. T., Krajcarz, M., Ginter, B., Goslar, T. & Wojtal, P. Towards a Chronology of the Jerzmanowician—a New Series of Radiocarbon Dates from Nietoperzowa Cave (Poland). *Archaeometry* **60**, 383-401, doi:10.1111/arcm.12311 (2018).

33 Kot, M. *et al.* Chronostratigraphy of Jerzmanowician. New data from Koziarnia Cave, Poland. *Journal of Archaeological Science: Reports* **38**, 103014, doi:https://doi.org/10.1016/j.jasrep.2021.103014 (2021).

34 Davies, W., White, D., Lewis, M. & Stringer, C. Evaluating the transitional mosaic: frameworks of change from Neanderthals to Homo sapiens in eastern Europe. *Quaternary Sci. Rev.* **118**, 211-242, doi:https://doi.org/10.1016/j.quascirev.2014.12.003 (2015).

35 Marks, L., Gałązka, D. & Woronko, B. Climate, environment and stratigraphy of the last Pleistocene glacial stage in Poland. *Quatern. Int.* **420**, 259-271, doi:https://doi.org/10.1016/j.quaint.2015.07.047 (2016).

36 Wohlfarth, B. A review of Early Weichselian climate (MIS 5d-a) in Europe. *Technical report/Svensk kärnbränslehantering AB* **44** (2013).

37 Marks, L. *et al.* Late Pleistocene climate of Poland in the mid-European context. *Quatern. Int.* **504**, 24-39, doi:https://doi.org/10.1016/j.quaint.2018.01.024 (2019).

38 Picin, A. & Cascalheira, J. in *Short-Term Occupations in Paleolithic Archaeology: Definition and Interpretation* (eds João Cascalheira & Andrea Picin) 1-15 (Springer International Publishing, 2020).

39 Picin, A. *et al.* Neanderthal mobile toolkit in short-term occupations at Teixoneres Cave (Moia, Spain). *Journal of Archaeological Science: Reports* **29**, 102165, doi:https://doi.org/10.1016/j.jasrep.2019.102165 (2020).

40 Neruda, P. *Middle Palaeolithic in Moravian Caves*. 249 (Muni Press, 2011).

41 Picin, A. Short-term occupations at the lakeshore: a technological reassessment of the open–air site Königsaue (Germany). *Quartär*, 7-32, doi:10.7485/QU63_1 (2016).

42 Valde-Nowak, P. *et al.* Late Middle Palaeolithic occupations in Ciemna Cave, southern Poland. *Journal of Field Archaeology* **41**, 193-210, doi:10.1080/00934690.2015.1101942 (2016).

43 Valde-Nowak, P. & Cieśla, M. in *Short-Term Occupations in Paleolithic Archaeology: Definition and Interpretation* (eds João Cascalheira & Andrea Picin) 105-120 (Springer, 2020).

44 Wiśniewski, A. *et al.* Looking for provisioning places of shaped tools of the late Neanderthals: A study of a Micoquian open-air site, Pietraszyn 49a (southwestern Poland). *Comptes Rendus Palevol* **18**, 367-389, doi:https://doi.org/10.1016/j.crpv.2019.01.003 (2019).

45 Valde-Nowak, P., Nadachowski, A. & Wolsan, M. Upper Palaeolithic boomerang made of a mammoth tusk in south Poland. *Nature* **329**, 436-438 (1987).

46 Kozłowski, J. K. & Sobczyk, K. The Upper Paleolithic site Krakow e Spadzista street C2. Excavations 1980. *Prace Archeologiczne* **42**, 7-68 (1987).

47 Wilczyński, J., Wojtal, P., Sobieraj, D. & Sobczyk, K. Kraków Spadzista trench C2: New research and interpretations of Gravettian settlement. *Quatern. Int.* **359-360**, 96-113, doi:https://doi.org/10.1016/j.quaint.2014.08.025 (2015).

48 Cyrek, K. *et al.* Excavation in the Deszczowa Cave (Kroczyckie Rocks, Czêstochowa Upland, Central Poland). *Folia Quaternaria* **71**, 5-84 (2000).

49 Kozłowski, L. *Starsza epoka kamienna w Polsce (paleolit). Prace Komisji Archeologicznej 1*. Vol. 1 1-53 (Poznanskie Towarzystwo Przyjaciol Nauk, 1922).

50 Zawisza, J. La caverne de Mammoth en Pologne. *Mèmoire de la Societè d'Anthropologie de Paris* **2**, 439-447 (1878).

51 Allsworth-Jones, P. *The Szeletian: And the Transition from Middle to Upper Palaeolithic in Central Europe*. (Oxford University Press, USA, 1986).

52 Flas, D. in *Encyclopedia of Global Archaeology* (ed Claire Smith) 5499-5514 (Springer New York, 2014).

53 Kozłowski, J. K. in *Towards a definition of the Aurignacian.* Vol. 45 (eds O Bar-Yosef & J Zilhão) 21-34 (Instituto Português de Arqueologia, 2002).

54 Burns, D. A. & Ciurczak, E. W. *Handbook of near-infrared analysis*. (CRC press, 2007).

55 Sponheimer, M. *et al.* Saving Old Bones: a non-destructive method for bone collagen prescreening. *Scientific Reports* **9**, 13928, doi:10.1038/s41598-019-50443-2 (2019).

56 Fewlass, H. *et al.* Direct radiocarbon dates of mid Upper Palaeolithic human remains from Dolní Věstonice II and Pavlov I, Czech Republic. *Journal of Archaeological Science: Reports* **27**, 102000, doi:https://doi.org/10.1016/j.jasrep.2019.102000 (2019).

57 Faris, F. *et al.* Non-invasive in vivo near-infrared optical measurement of the penetration depth in the neonatal head. *Clinical Physics and Physiological Measurement* **12**, 353-358, doi:10.1088/0143-0815/12/4/005 (1991).

58 Afara, I. O. *et al.* Characterizing human subchondral bone properties using near-infrared (NIR) spectroscopy. *Scientific Reports* **8**, 9733, doi:10.1038/s41598-018-27786-3 (2018).

59 Turner-Walker, G. H. *The characterisation of fossil bone*, Durham University, (1993).

60 Turner-Walker, G. & Jans, M. Reconstructing taphonomic histories using histological analysis. *Palaeogeography, Palaeoclimatology, Palaeoecology* **266**, 227-235, doi:https://doi.org/10.1016/j.palaeo.2008.03.024 (2008).

61 Suarez, C. A. & Kohn, M. J. Caught in the act: A case study on microscopic scale physicochemical effects of fossilization on stable isotopic composition of bone. *Geochimica et Cosmochimica Acta* **268**, 277-295, doi:https://doi.org/10.1016/j.gca.2019.10.008 (2020).

62 Bronk Ramsey, C. Dealing with Outliers and Offsets in Radiocarbon Dating. *Radiocarbon* **51**, 1023-1045, doi:10.1017/S0033822200034093 (2009).

63 Reimer, P. J. *et al.* The INTCAL20 northern hemisphere radiocarbon age calibration curve (0–55 cal kBP). *Radiocarbon* **62**, 1-33, doi:10.1017/RDC.2020.41 (2020).

64 Bolus, M. in *Human Origin Sites and the World Heritage Convention in Eurasia II* Vol. 2 (ed Nuria Sanz) 32-49 (UNESCO, 2015).

65 Miller, C. E. *A tale of two Swabian caves. Geoarchaeological investigations at Hohle Fels and Geißenklösterle*. (Kerns Verlag, 2015).

66 Conard, N. J. Palaeolithic ivory sculptures from southwestern Germany and the origins of figurative art. *Nature* **426**, 830-832 (2003).

67 Conard, N. J., Malina, M. & Munzel, S. C. New flutes document the earliest musical tradition in southwestern Germany. *Nature* **460**, 737-740 (2009).

68 Wolf, S. & Heckel, C. Ivory Ornaments of the Aurignacian in Western Europe: Case studies from France and Germany. *L'Anthropologie* **122**, 348-373, doi:https://doi.org/10.1016/j.anthro.2017.12.003 (2018).

69 Higham, T. *et al.* Τesting models for the beginnings of the Aurignacian and the advent of figurative art and music: The radiocarbon chronology of Geißenklösterle. *J Hum. Evol.* **62**, 664-676, doi:10.1016/j.jhevol.2012.03.003 (2012).

70 Higham, T. *et al.* The timing and spatiotemporal patterning of Neanderthal disappearance. *Nature* **512**, 306-309, doi:10.1038/nature13621 (2014).

71 Discamps, E., Gravina, B. & Teyssandier, N. In the eye of the beholder: contextual issues for Bayesian modelling at the Middle-to-Upper Palaeolithic transition. *World Archaeology* **47**, 601-621, doi:10.1080/00438243.2015.1065759 (2015).

72 Higham, T. F. G. & Heep, G. S. Reply to: ‘In the eye of the beholder: contextual issues for Bayesian modelling at the Middle-to-Upper Palaeolithic transition’, by Discamps, Gravina and Teyssandier (2015). *World Archaeology* **51**, 126-133, doi:10.1080/00438243.2017.1329026 (2019).

73 Discamps, E., Gravina, B. & Teyssandier, N. Comments on Higham and Heep (2017): ‘Reply to: “In the eye of the beholder: contextual issues for Bayesian modelling at the middle-to-upper Palaeolithic transition”, by Discamps, Gravina and Teyssandier (2015)’. *World Archaeology* **51**, 134-139, doi:10.1080/00438243.2018.1509724 (2019).

74 Conard, N. J., Niven, L. B., Mueller, K. & Stuart, A. J. The chronostratigraphy of the Upper Paleolithic deposits at Vogelherd. *Mitteilungen der Gesellschaft für Urgeschichte* **12**, 73-86 (2003).

75 Conard, N. J. & Bolus, M. Radiocarbon dating the appearance of modern humans and timing of cultural innovations in Europe: new results and new challenges. *J Hum. Evol.* **44**, 331-371, doi:http://dx.doi.org/10.1016/S0047-2484(02)00202-6 (2003).

76 Conard, N. J. & Bolus, M. Radiocarbon dating the late Middle Paleolithic and the Aurignacian of the Swabian Jura. *J Hum. Evol.* **55**, 886-897 (2008).

77 Tartar, É., White, R., Chiotti, L., Cretin, C. & Mensan, R. Which Aurignacians were at Abri Blanchard (Sergeac, Dordogne, France)? Data from bone and antler artifacts in American collections and from new field operations. *PALEO. Revue d'archéologie préhistorique*, 309-331 (2014).

78 Bourrillon, R. *et al.* A new Aurignacian engraving from Abri Blanchard, France: Implications for understanding Aurignacian graphic expression in Western and Central Europe. *Quatern. Int.* **491**, 46-64, doi:https://doi.org/10.1016/j.quaint.2016.09.063 (2018).

79 White, R. in *The human revolution: behavioural and biological perspectives on the origins of modern humans* (eds C. Stringer & P. Mellars) 366-390 (Edinburgh University Press, 1989).

80 Talamo, S. *et al.* The new 14C chronology for the Palaeolithic site of La Ferrassie, France: the disappearance of Neanderthals and the arrival of Homo sapiens in France. *Journal of Quaternary Science* **35**, 961-973, doi:https://doi.org/10.1002/jqs.3236 (2020).

81 White, R. *et al.* Context and dating of Aurignacian vulvar representations from Abri Castanet, France. *Proceedings of the National Academy of Sciences* **109**, 8450-8455, doi:10.1073/pnas.1119663109 (2012).

82 O'Hara, J., White, R., Garrett, Z., Higham, T. & Roussot, A. The Aurignacian Site of the Abri de la Souquette (commune de Sergeac, Dordogne): A History of Archeology. *Palethnologie. Archéologie et sciences humaines*, 99-118 (2015).

83 O’Hara, J. F., White, R., Garrett, Z. S., Higham, T. & Roussot, A. The Aurignacian Site of the Abri de la Souquette (commune de Sergeac, Dordogne): A History of Archeology. *Palethnologie. Archéologie et sciences humaines* (2015).

84 Vezian, J. Les gisements de la grotte de Saint-Jean-de-Verges (Ariège). *Gallia préhistoire* **9**, 93-130 (1966).

85 Foucher, P. *et al.* De nouvelles dates 14C pour le gravettien des Pyrénées. *Bulletin de la Société préhistorique Ariège-Pyrénées* **56**, 35-44 (2002).

86 Vasilyev, S. V. & Gerasimova, M. in *Le Sungirien* (eds S.V. Vasilyev, A. Sinitsyn, & M Otte) 47-60 (ERAUL 147, 2017).

87 Zhitenev, V. in *Le Sungirien* (eds S.V. Vasilyev, A. Sinitsyn, & M. Otte) 73-84 (ERAUL 147, 2017).

88 Nalawade-Chavan, S., McCullagh, J. & Hedges, R. New Hydroxyproline Radiocarbon Dates from Sungir, Russia, Confirm Early Mid Upper Palaeolithic Burials in Eurasia. *PLOS ONE* **9**, e76896, doi:10.1371/journal.pone.0076896 (2014).

89 Pitulko, V. V., Pavlova, E. Y., Nikolskiy, P. A. & Ivanova, V. V. The oldest art of the Eurasian Arctic: personal ornaments and symbolic objects from Yana RHS, Arctic Siberia. *Antiquity* **86**, 642-659, doi:10.1017/S0003598X00047827 (2012).

90 Basilyan, A. E., Anisimov, M. A., Nikolskiy, P. A. & Pitulko, V. V. Wooly mammoth mass accumulation next to the Paleolithic Yana RHS site, Arctic Siberia: its geology, age, and relation to past human activity. *J. Archaeol. Sci.* **38**, 2461-2474, doi:https://doi.org/10.1016/j.jas.2011.05.017 (2011).

91 Pitulko, V. V. *et al.* The Yana RHS Site: Humans in the Arctic Before the Last Glacial Maximum. *Science* **303**, 52-56, doi:10.1126/science.1085219 (2004).

92 Pitulko, V. V., Pavlova, E. Y. & Nikolskiy, P. A. Mammoth ivory technologies in the Upper Palaeolithic: a case study based on the materials from Yana RHS, Northern Yana-Indighirka lowland, Arctic Siberia. *World Archaeology* **47**, 333-389, doi:10.1080/00438243.2015.1030508 (2015).

93 Locke, M. Structure of ivory. *Journal of Morphology* **269**, 423-450, doi:https://doi.org/10.1002/jmor.10585 (2008).

94 Su, X. W. & Cui, F. Z. Hierarchical structure of ivory: from nanometer to centimeter. *Materials Science and Engineering: C* **7**, 19-29, doi:https://doi.org/10.1016/S0928-4931(98)00067-8 (1999).
